# Supplementary material for: ‘Function First’: how to promote physical activity and physical function in people with long-term conditions managed in primary care? A study combining realist and co-design methods
Source: BMJ Open. 2021 Jul 27;11(7):e046751. doi: 10.1136/bmjopen-2020-046751 (PMC8317101; doi:10.1136/bmjopen-2020-046751)
Supplement: Supplementary data [file bmjopen-2020-046751supp001.pdf]

Supplementary figure 1: Visual storyboard project summary

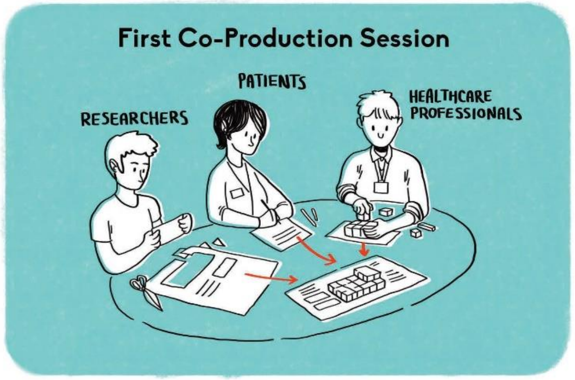

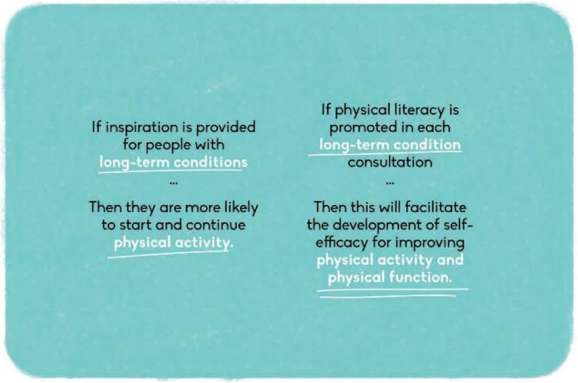

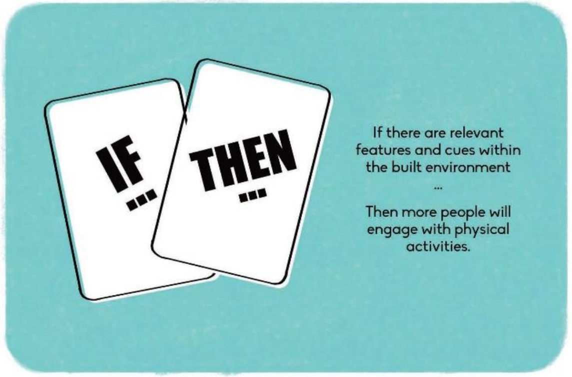

BMJ Publishing Group Limited (BMJ) disclaims all liability and responsibility arising from any reliance placed on this supplemental material which has been supplied by the author(s)

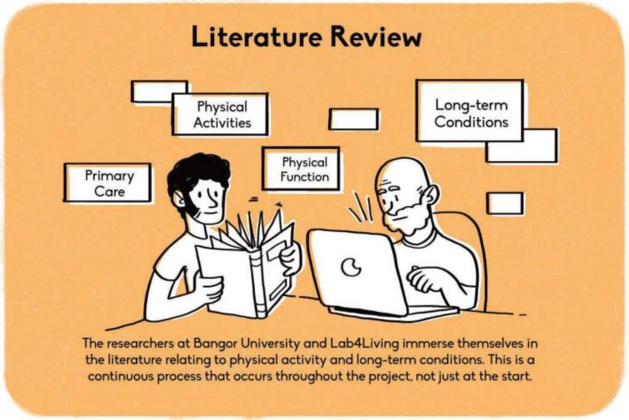

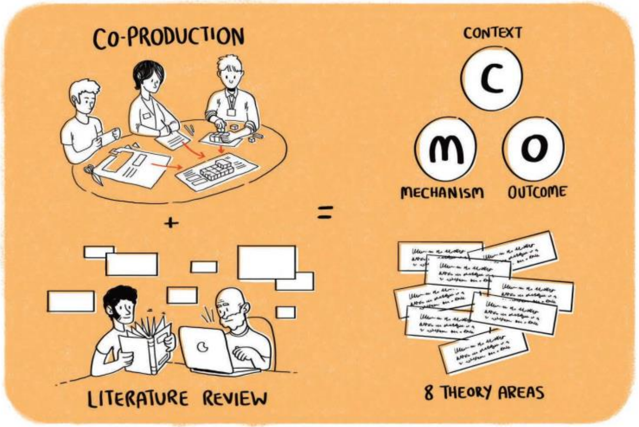

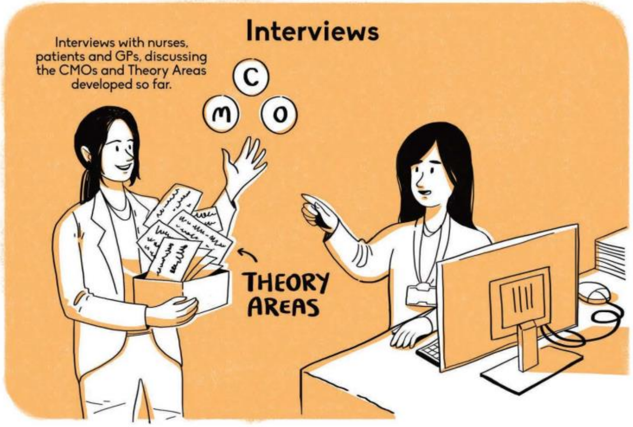

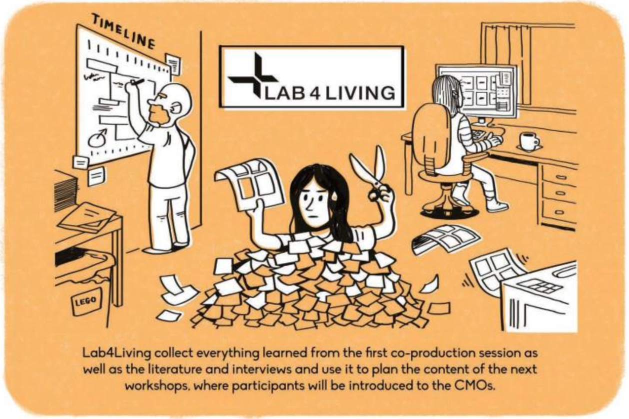

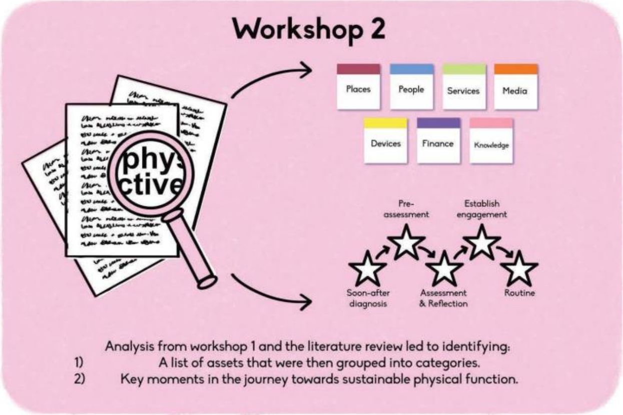
Workshop 2
Places
People
Services
Media
Devices
Finance
Knowledge
Pre-assessment
Establish engagement
Soon-after diagnosis
Assessment & Reflection
Routine
Analysis from workshop 1 and the literature review led to identifying:
1) A list of assets that were then grouped into categories.
2) Key moments in the journey towards sustainable physical function.

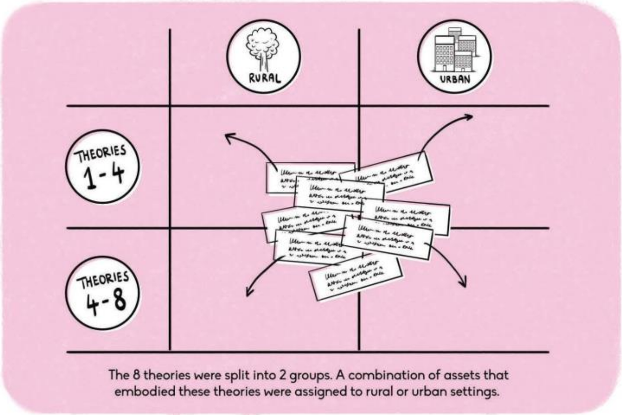
RURAL
URBAN
THEORIES 1-4
THEORIES 4-8
The 8 theories were split into 2 groups. A combination of assets that embodied these theories were assigned to rural or urban settings.

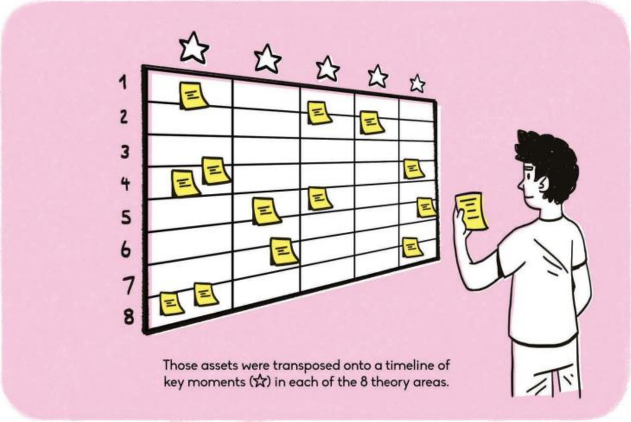
1
2
3
4
5
6
7
8
Those assets were transposed onto a timeline of key moments (☆) in each of the 8 theory areas.

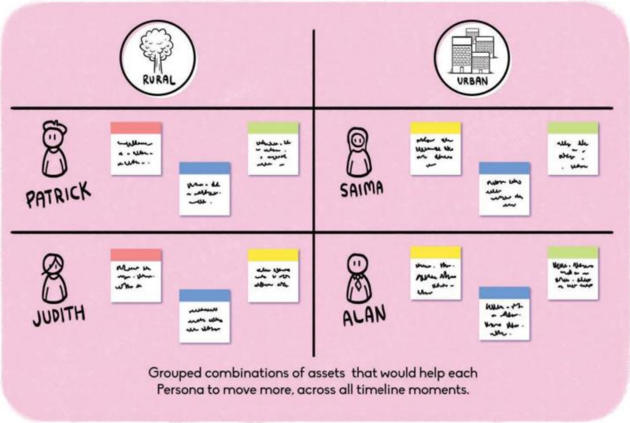
RURAL
URBAN
PATRICK
SAIMA
JUDITH
ALAN
Grouped combinations of assets that would help each Persona to move more, across all timeline moments.

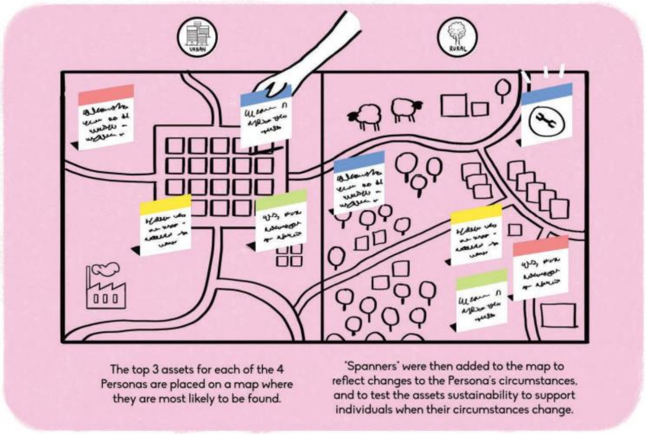
The top 3 assets for each of the 4 Personas are placed on a map where they are most likely to be found.
'Spanners' were then added to the map to reflect changes to the Persona's circumstances, and to test the assets sustainability to support individuals when their circumstances change.

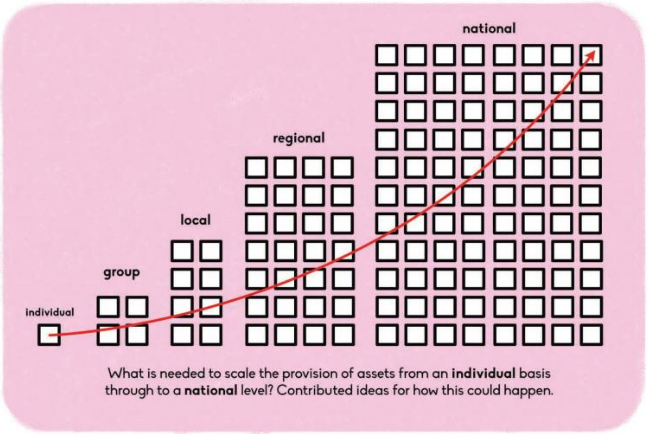
national
regional
local
group
individual
What is needed to scale the provision of assets from an **individual** basis through to a **national** level? Contributed ideas for how this could happen.

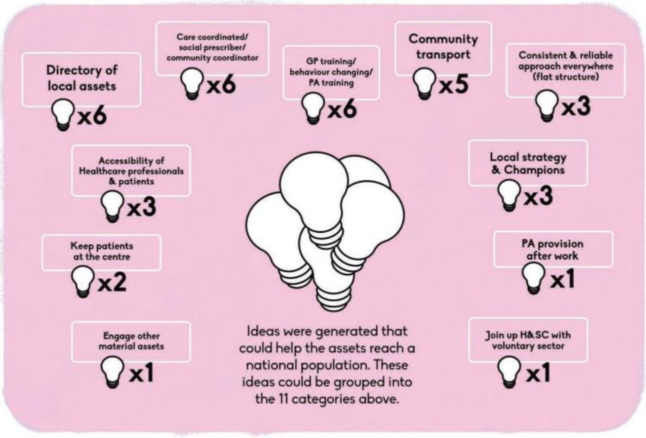
Directory of local assets x6
Care coordinated/ social prescriber/ community coordinator x6
GP training/ behaviour changing/ PA training x6
Community transport x5
Consistent & reliable approach everywhere (flat structure) x3
Accessibility of Healthcare professionals & patients x3
Local strategy & Champions x3
Keep patients at the centre x2
PA provision after work x1
Engage other material assets x1
Join up H&SC with voluntary sector x1
Ideas were generated that could help the assets reach a national population. These ideas could be grouped into the 11 categories above.

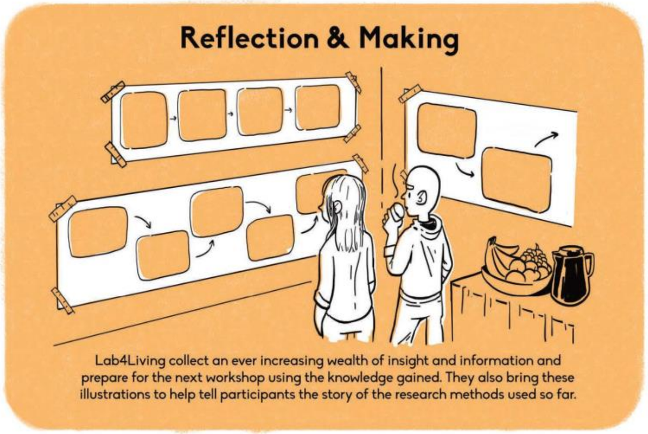
Reflection & Making
Lab4Living collect an ever increasing wealth of insight and information and prepare for the next workshop using the knowledge gained. They also bring these illustrations to help tell participants the story of the research methods used so far.

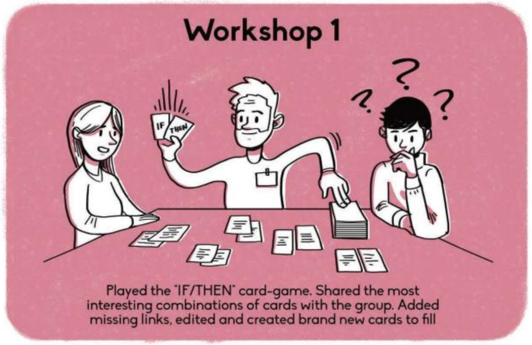

Key Findings:

- Appropriate delivery and accessible language at the first contact is vital.
- Change the cards from "will" to "may". There is no "one size fits all."
- Social function can be linked to physical function.

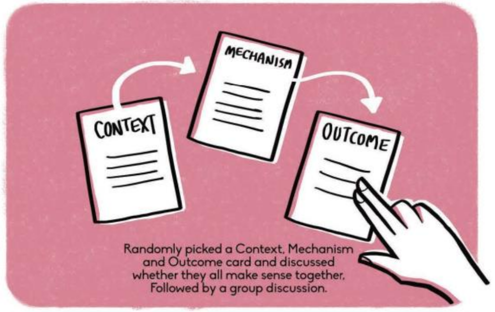

Key Findings:

- Family and personal relationships affect readiness to engage in both positive and negative ways.
- We need to ensure people don't just agree in the GP appointment without taking action afterwards.
- GPs and nurses need to be supported in developing realistic goals shared between both professional and patient.
- Focus on what is possible, rather than focussing on what has been lost.

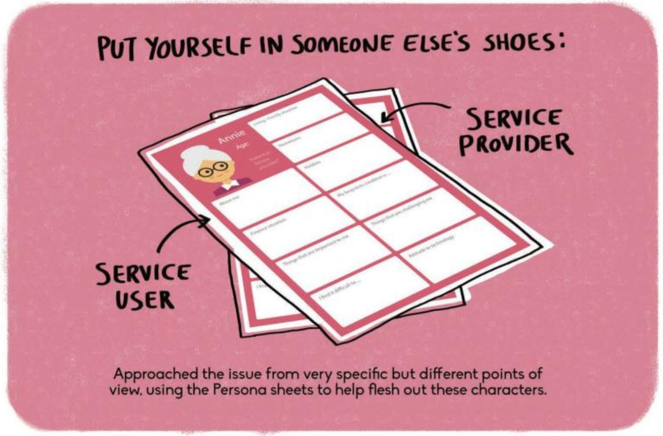

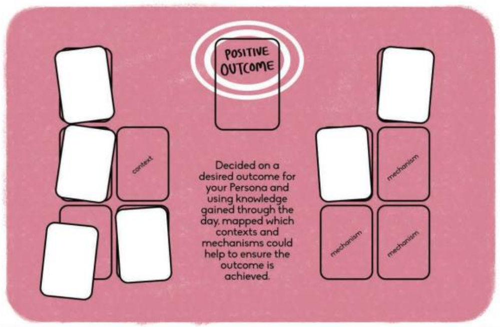

Key Findings:

- People juggle multiple societal roles, and sometimes don't prioritise their own needs.
- Interventions need to take into account personal preferences of the individual and need to be meaningful in a number of different ways for the person to carry on doing it.
- Once there is readiness to change, the need to ask for help is important. Readiness is only the first step in the process.
- Volunteering can be a reciprocal activity. It can address loneliness and provide purpose at the end of your work-life.
- Develop a community/society where anyone can go out (be active) and feel safe.
- What can people offer each other in the community? Do they have access to peer relationships and networks?

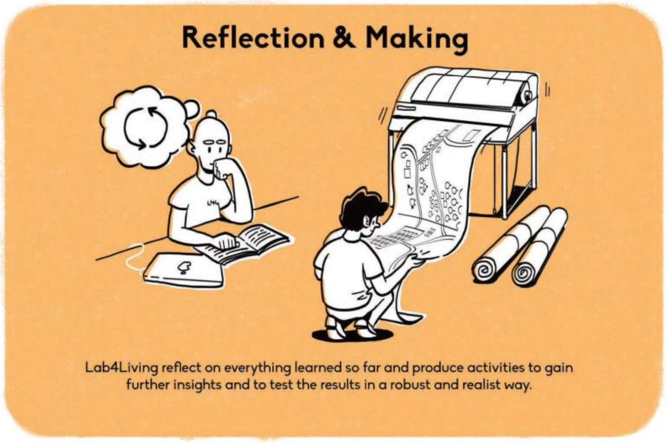

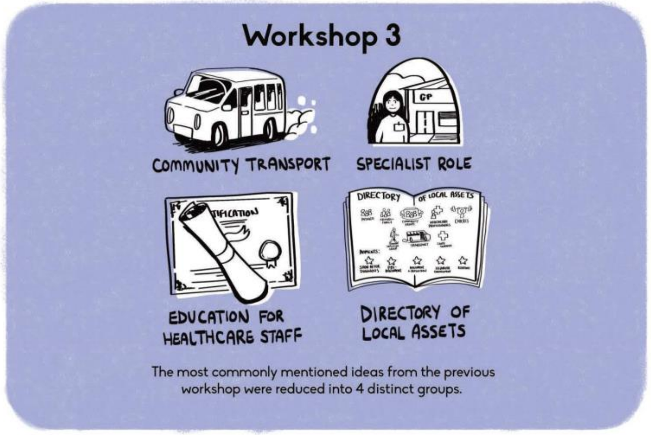
Workshop 3
COMMUNITY TRANSPORT
SPECIALIST ROLE
EDUCATION FOR HEALTHCARE STAFF
DIRECTORY OF LOCAL ASSETS
The most commonly mentioned ideas from the previous workshop were reduced into 4 distinct groups.

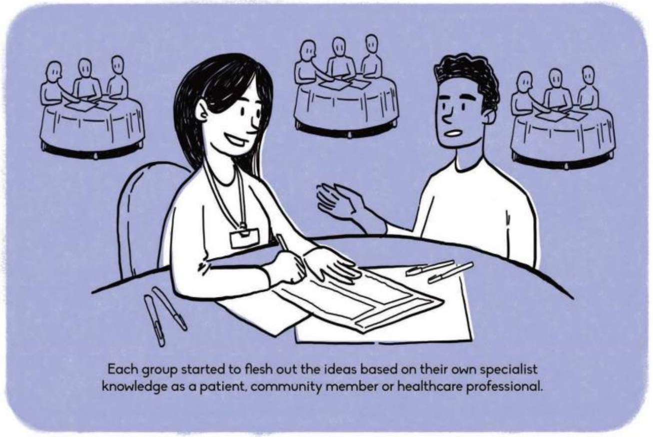
Each group started to flesh out the ideas based on their own specialist knowledge as a patient, community member or healthcare professional.

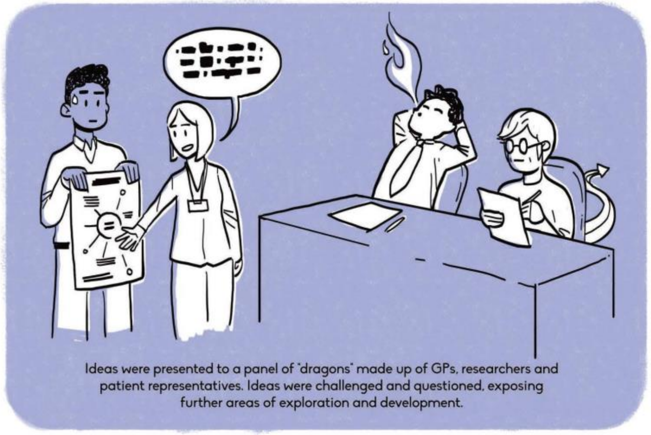
Ideas were presented to a panel of 'dragons' made up of GPs, researchers and patient representatives. Ideas were challenged and questioned, exposing further areas of exploration and development.

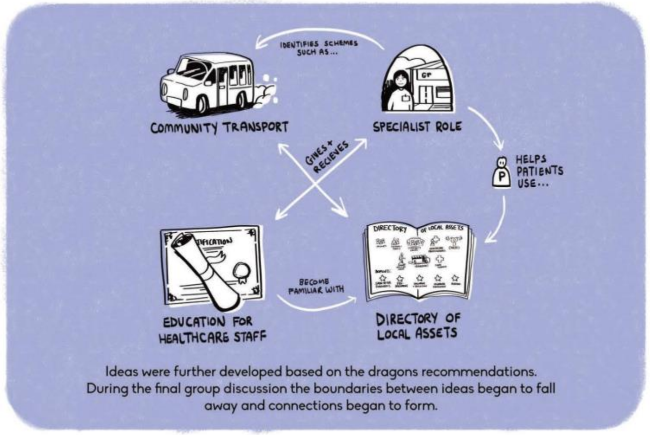
IDENTIFIES SCHEMES SUCH AS...
COMMUNITY TRANSPORT
SPECIALIST ROLE
GIVES + RECIEVES
HELPS PATIENTS USE...
BECOME FAMILIAR WITH
EDUCATION FOR HEALTHCARE STAFF
DIRECTORY OF LOCAL ASSETS
Ideas were further developed based on the dragons recommendations. During the final group discussion the boundaries between ideas began to fall away and connections began to form.

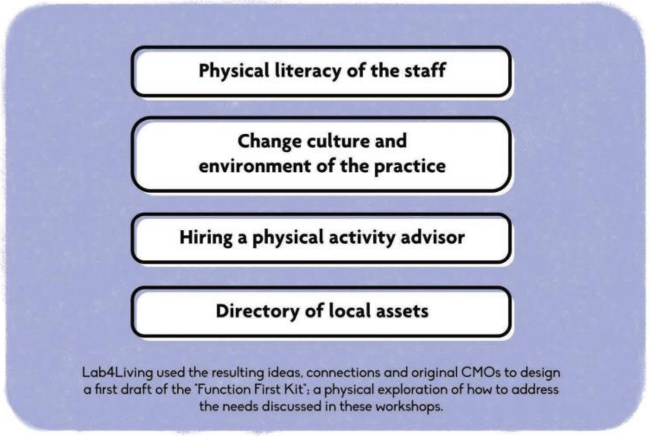
Physical literacy of the staff
Change culture and environment of the practice
Hiring a physical activity advisor
Directory of local assets
Lab4Living used the resulting ideas, connections and original CMOs to design a first draft of the 'Function First Kit'; a physical exploration of how to address the needs discussed in these workshops.

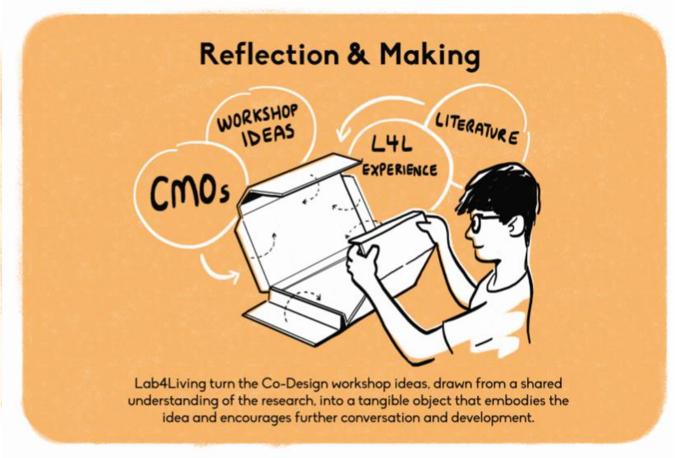
Reflection & Making
WORKSHOP IDEAS
LITERATURE
L4L EXPERIENCE
CMOs
Lab4Living turn the Co-Design workshop ideas, drawn from a shared understanding of the research, into a tangible object that embodies the idea and encourages further conversation and development.

Knowledge Mobilisation Session

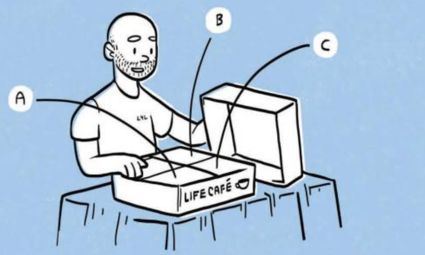

The session started with a walkthrough of previous interventions designed by Lab4Living including Life Cafe to facilitate conversations around end of life and P.E.A.R.L which provides structured reflection which fits into the busy days of healthcare professionals.

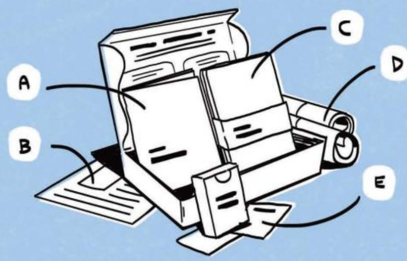

With an understanding of what is possible in terms of physical interventions, Lab4Living presented the Function First box in it's initial form, talking through each part, and how it links back to the co-production which has taken place so far.

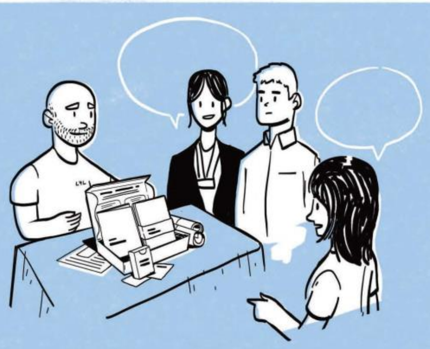

After an introduction to the intervention, a group discussion took place to further analyse, improve and add to the materials designed so far.

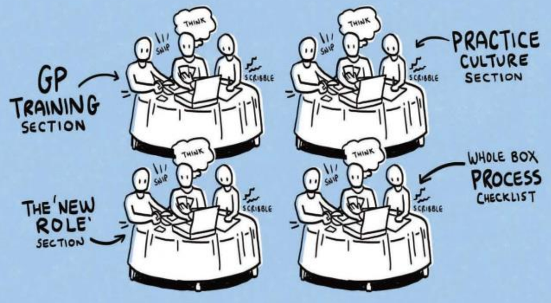

The workshop attendants split into smaller groups and each took a different area of focus. Alongside the designers at Lab4Living they created new content and resources.

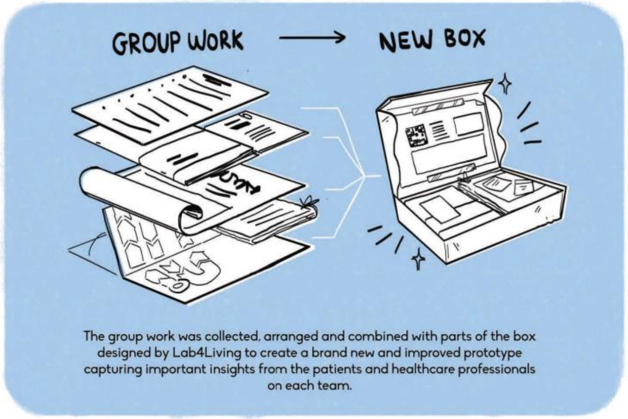

The group work was collected, arranged and combined with parts of the box designed by Lab4Living to create a brand new and improved prototype capturing important insights from the patients and healthcare professionals on each team.

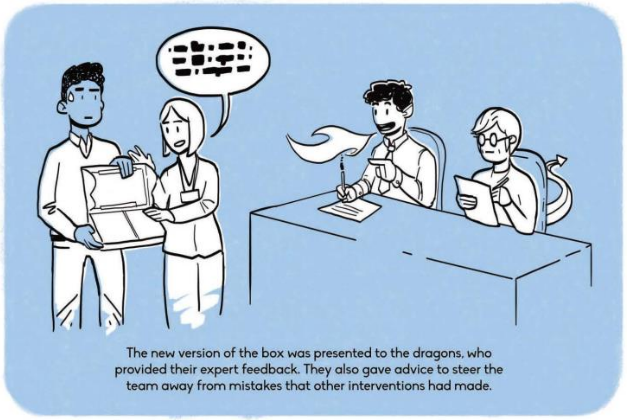
The new version of the box was presented to the dragons, who provided their expert feedback. They also gave advice to steer the team away from mistakes that other interventions had made.

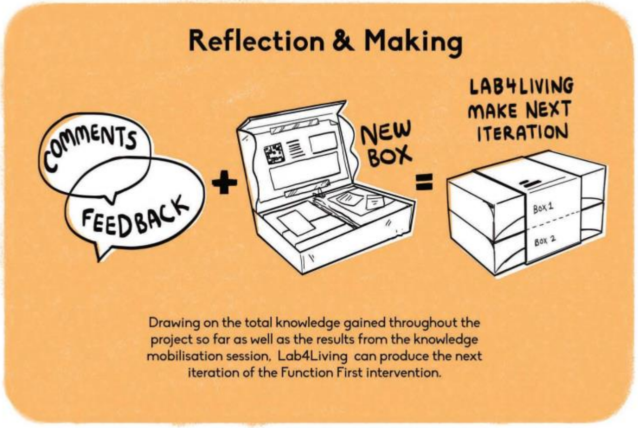

Drawing on the total knowledge gained throughout the project so far as well as the results from the knowledge mobilisation session, Lab4Living can produce the next iteration of the Function First intervention.
