# Supplementary material for: ‘Function First’: how to promote physical activity and physical function in people with long-term conditions managed in primary care? A study combining realist and co-design methods
Source: BMJ Open. 2021 Jul 27;11(7):e046751. doi: 10.1136/bmjopen-2020-046751 (PMC8317101; doi:10.1136/bmjopen-2020-046751)
Supplement: Supplementary data [file bmjopen-2020-046751supp003.pdf]

Supplementary table 1: Stakeholder contribution to the theory-building workshops and telephone interviews, and cumulative contribution of individual stakeholders to the co-design and knowledge mobilisation workshops. * = also took part in an individual interview (n = 1 public contributor, n = 1 representative from relevant third sector organisation, who also had a long-term condition, n = 1 GP, n = 1 researcher). ** = also took part in co-design workshops 2 and 3 and the knowledge mobilisation workshop (n = 1). *** = also took part in a co-design workshop 3 and the knowledge mobilisation workshop. [#] = member of research team. Co-Chief Investigators BL (health services researcher) and NW (academic GP) contributed as stakeholders to all study workshops.

**Theory-building workshops and stakeholder interviews**					**Co-design and knowledge mobilisation**			
**Stakeholder representation**	Theory-building workshop 1 (N = 10)	Theory-building workshop 2 (N = 13)	Telephone interview (N = 10)	**Stakeholder representation**	Co-design workshop 1 (N = 9)	Co-design workshop 2 (N = 14)	Co-design workshop 3 (N = 11)	Knowledge mobilisation workshop (N = 12)
**Public contributor, long-term condition**	n = 5*	n = 6**	n = 3	**Public contributor 1, long-term condition**	✓	✓	✓	✓
**GP**	n = 1	n = 1*	n = 3	**Public contributor 2, long-term condition**	✓	✓	-	✓
**Leisure centre manager**	n = 1	-	-	**Public contributor 3, long-term condition**	✓	-	-	-
**Sport and outdoor recreation division of local council**	n = 1	-	-	**Public contributor 4, long-term condition**	-	✓	✓	✓

**Health and social care public representation group**	n = 1	-	-		**Public contributor 5, long-term condition#**	-	✓	✓	✓
**Practice manager**	-	n = 1	-		**Public contributor 6, long-term condition**	-	✓	-	-
**Researcher, social care**	-	n = 1	-		**Public contributor 7, long-term condition**	-	✓	-	-
**Occupational therapist**	-	n = 1	-		**General Practitioner**	-	✓	✓	✓
**Third sector organisation**	n = 1*	n = 1	-		**Practice nurse**	-	✓	-	-
**Physiotherapist**	-	n = 1	-		**Practice manager**	-	✓	-	-
**Engagement officer**	-	n = 1	-		**Physician associate**	✓	-	✓	✓
**Primary care practice nurse**	-	-	n = 2		**Physiotherapist**	-	✓	-	-
**Primary care healthcare assistant**	-	-	n = 1		**Primary care cluster lead, coordinator of voluntary services**	✓	✓	✓	-
**Researcher, pedagogy**	-	-	n = 1***		**Exercise referral scheme co-ordinator**	✓	✓	✓	-

	**Life coach**	-	✓	-	-
	**Researcher, pedagogy***	-	-	✓	✓
	**Researcher, physical activity promotion**	-	-	-	✓
	**Researcher, clinical exercise physiology#**	-	✓	-	-
	**Researcher, medical sociology#**	-	-	✓	✓
	**Researcher, health psychology#**	✓	-	✓	✓
	**Researcher, nursing, rehabilitation and implementation#**	✓	-	-	-
	**Researcher, sports physiology#**	✓	-	✓	✓
	**Researcher, information science#**	-	-	-	✓
				**5 'Dragon's'/independent advisors included representation from:**	**4 'Dragon's'/independent advisors included representation from**

					• **2 public contributors with long-term conditions**<br>• **General practice**<br>• **Public Health**<br>• **Physical activity promotion research**	**organisations and professional bodies relevant to:**<br>• **General practice**<br>• **Public health**<br>• **Nursing**<br>• **Physiotherapy**
