# Supplementary material for: ‘Function First’: how to promote physical activity and physical function in people with long-term conditions managed in primary care? A study combining realist and co-design methods
Source: BMJ Open. 2021 Jul 27;11(7):e046751. doi: 10.1136/bmjopen-2020-046751 (PMC8317101; doi:10.1136/bmjopen-2020-046751)

Supplementary figure 2: Example models built by participants in the theory-building workshops to reflect on and describe their interpretation of: A) what physical function meant to them, B) how they maintained physical function and C) an example of a 'shared landscape'.

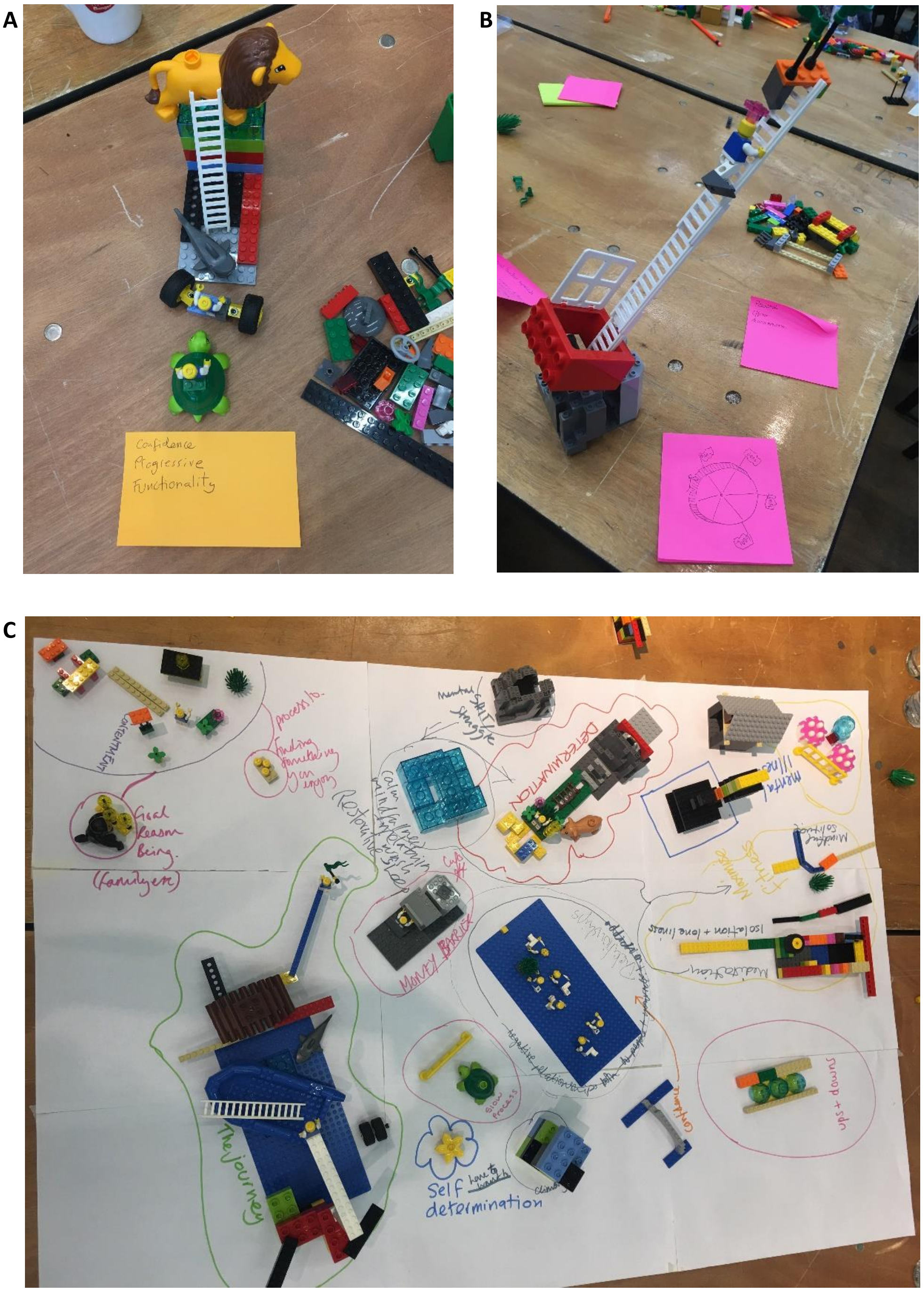

Supplement: Supplementary data [file bmjopen-2020-046751supp002.pdf]
