# Supplementary material for: ‘Function First’: how to promote physical activity and physical function in people with long-term conditions managed in primary care? A study combining realist and co-design methods
Source: BMJ Open. 2021 Jul 27;11(7):e046751. doi: 10.1136/bmjopen-2020-046751 (PMC8317101; doi:10.1136/bmjopen-2020-046751)
Supplement: Supplementary data [file bmjopen-2020-046751supp004.pdf]

Supplementary file 4: If…Then statements

**Overarching theory areas**
**Physical literacy:** the motivation, confidence, physical competence, knowledge and understanding to value and take responsibility for engagement in physical activity for life (Jones et al, 2018)
**International Classification of Functioning:** The International Classification of Functioning, Disability and Health, known more commonly as ICF, is the World Health Organisation's framework for measuring health and disability at both individual and population levels (World Health Organisation, 2019).
**If…then statements**
**Level: Local engagement outside of practice**
If people have access to a variety of physical activity opportunities, then they will be more likely to pursue opportunities to be more physically active
If there are sufficient places on exercise referral schemes and physiotherapy clinics, then people will receive timely interventions to improve physical function
**Level: Whole practice**
If there is a culture of physical literacy in the practice, then patients are more likely to receive interventions that promote physical activity
If a range of referral options are available according to patients' functional status (pyramid model), then more patients will receive appropriate interventions to enhance their physical function
If there are sufficient staff working in the practice who are trained to promote physical literacy, then more patients will receive timely physical activity interventions
If staff are trained in a variety of techniques to encourage physical activity (goal-setting, coaching, motivational interviewing), then they are more likely to promote physical literacy
If the practice has good links with outside agencies/social networks providing a wide range of physical activities in the local area, then more people will engage with physical activities
If there are relevant features and cues within the built environment, then physical activity and physical function will improve
**Level: Health professional consultations with people with long-term conditions**
If physical literacy is promoted during routine primary care management of long-term conditions, then physical activity will increase and physical functioning will improve
If consultations with people with long-term conditions consistently address physical activity and physical function, then people will be more likely to prioritise this element of management
If physical literacy is promoted in each long-term condition consultation, then physical literacy will be integrated into routine care

If self-efficacy and goal-setting are promoted as part of routine care, then physical activity interventions will be more meaningful to patients and uptake and adherence will increase.
If pacing is used in physical activity promotion, incorporating periods of rest and recuperation, then patients with low energy levels and low fitness levels are more likely to persist with physical activity interventions
**Level: Individual patients**
Physical
If physical activity advice is tailored to patients' own priorities for functioning, then they are more likely to start and continue physical activities
If a functional approach is adopted to the management of long-term conditions, then interventions will be more relevant and tailored towards functional limitations that are meaningful to the individual
Psychological
If being physically active is linked to the freedom, well-being and joy associated with having physical function, then people with long-term conditions will better identify with the reasoning for being physically active and be more likely to continue.
If enjoyment is emphasised as an important element of physical activity, then people will be more motivated to pursue physical activity opportunities and therefore maintain physical function.
If people have built an engrained physical activity 'identity' through previous experiences, then they will be more likely to be motivated to pursue physical activity opportunities
If people with long-term conditions are supported to develop contingency plans for unpredictable circumstances, then they will be more likely to be able to persist with physical activity.
If people have self-efficacy for maintaining and improving physical activity and physical function, then they will be more likely to lead a physically active lifestyle.
If anxiety about exercising is reduced (e.g. fatigue, soreness, shortness of breath, tachycardia) through educational interventions about the normal physiological effect of exercise, then people are more likely to start and continue being physically active.
If people have recently been diagnosed with a long-term condition or experienced a life event, then a window of opportunity where people are most receptive can be utilised to promote physical activity in order to maintain and improve physical function.
If low mood reduces the motivation to exercise, then improving mood will increase physical activity participation
If inspiration (indirect and direct) is provided for people with long-term conditions, then this will facilitate the development of self-efficacy for improving physical activity and physical function
If people develop a sense of agency relating to physical activity and physical function, then they will be more likely to pursue physical activity opportunities

Social
If people feel supported by family or friends, then they are more likely to start and continue physical activity
If people perform physical activity or exercise in groups, then they are more likely to continue
If people with long-term conditions are labelled/stereotyped less, then perceived and actual restrictions will reduce, encouraging improvements in physical activity and physical function
