# Supplementary material for: ‘Function First’: how to promote physical activity and physical function in people with long-term conditions managed in primary care? A study combining realist and co-design methods
Source: BMJ Open. 2021 Jul 27;11(7):e046751. doi: 10.1136/bmjopen-2020-046751 (PMC8317101; doi:10.1136/bmjopen-2020-046751)
Supplement: Supplementary data [file bmjopen-2020-046751supp005.pdf]

Supplementary table 3: Initial inclusion/exclusion criteria

**Screening tool: Function First**	
**Primary research question:**<br>**What is the role of primary care in reducing the decline in physical function and physical activity for people with long-term conditions: what works, for whom and in what circumstances?**<br>**Secondary research objectives:**<br>**a)** To identify and produce a taxonomy of physical activity interventions that aim to reduce functional decline in people with long-term conditions managed in primary care.<br>**b)** To uncover the complexity associated with the range of physical activity interventions in primary care, and how they directly or indirectly affect the physical functioning of people with long-term conditions.<br>**c)** To identify the mechanisms through which interventions bring about functional improvements in people with long-term conditions, and the circumstances associated with how the interventions are organised and operate within different primary care contexts.<br>**d)** To understand the potential impacts of these interventions across primary care and other settings, such as secondary healthcare and social care, paying attention to the conditions which influence how they operate.	
**Questions – key elements**	
**Population (P) and Conditions**	**Include:**<br>**People with long-term conditions** or **people who are likely to have a long-term condition** (e.g. frail or 'pre-frail/at risk of frailty', reduced mobility, living in care home).<br>'Long-term conditions or chronic diseases are conditions for which there is currently no cure, and which are managed with drugs and other treatment, for example: diabetes, chronic obstructive pulmonary disease, arthritis, hypertension' (DOH/Kings Fund) – including cancer, addiction/substance abuse, mental health conditions, heart conditions, chronic pain, stroke, obesity, learning disabilities, multiple sclerosis.<br>**OR**<br>**People promoting physical activity to people with long-term conditions**<br>GPs, nurse practitioners, practice nurses, physician associates, physio/occupational therapists, multi-disciplinary teams, other primary care staff, exercise physiologists, exercise professionals, exercise instructors, coaches, people providing exercise opportunities in the community, professionals providing rehabilitation interventions (eg cardiac or pulmonary rehabilitation).
	**Exclude:** Healthy but sedentary people; injured/acutely unwell people (e.g. injury, mechanical problems, fracture or sepsis); pre-conditions (e.g. pre-diabetes, osteopenia); pregnancy; menopause; children and adolescents aged <18 years; animal studies.
**Interventions (I)**	**Include:** **Physical activity interventions** designed to improve physical function and physical activity.
	**Exclude:** Physiotherapy, physical therapy, mechanical/manual therapy without any physical activity/exercise intervention. Interventions with limited transferability to NHS primary care, pharmacological agents, technical, high-cost equipment, short-term rehabilitation following injury, fracture, sepsis, post-operative; breathing exercises.
**Outcomes (O)**	**Include:** Any outcomes targeting improving physical activity or physical function (including psychological and social outcomes).
	**Exclude:** Physiological measurements (e.g. lung function, cardiovascular function, physiology laboratory), or biochemical markers (e.g. blood measures of disease activity/severity) or therapy-specific (e.g. motor control, range of motion/biomechanical, cognitive outcomes only).
**Settings (S)**	**Include:** Studies in primary care, general medical practice, community settings, care homes, intermediate care including reablement (recovering from hospital stay), community or outpatient cardiac or pulmonary rehabilitation).
	**Exclude:** Physiology laboratory, hospital in-patient (e.g. exercise during dialysis or chemotherapy).
**Study type**	**Include:** Any study design, including reviews, qualitative papers about barriers and facilitators and especially theory-rich papers with potential mechanisms, process evaluations, etc.
	**Exclude:** N/A (to screen later for theoretical richness)
