# Supplementary material for: ‘Function First’: how to promote physical activity and physical function in people with long-term conditions managed in primary care? A study combining realist and co-design methods
Source: BMJ Open. 2021 Jul 27;11(7):e046751. doi: 10.1136/bmjopen-2020-046751 (PMC8317101; doi:10.1136/bmjopen-2020-046751)
Supplement: Supplementary data [file bmjopen-2020-046751supp006.pdf]

Supplementary table 4: List of included papers

Author and year	Title
Allen 2012[1]	Patient and provider interventions for managing osteoarthritis in primary care: protocols for two randomized controlled trials
Allen 2017 [2]	Patient, provider, and combined interventions for managing osteoarthritis in primary care: A cluster randomized trial
Andryukhin 2010 [3]	The impact of a nurse-led care programme on events and physical and psychosocial parameters in patients with heart failure with preserved ejection fraction: A randomized clinical trial in primary care in Russia
Arden 2017 [4]	Evaluation of a rolling rehabilitation programme for patients with non-specific low back pain in primary care: an observational cohort study
Åsenlöf 2005 [5]	Individually tailored treatment targeting activity, motor behavior, and cognition reduces pain-related disability: A randomized controlled trial in patients with musculoskeletal pain
Åsenlöf 2009 [6]	Long-term follow-up of tailored behavioural treatment and exercise based physical therapy in persistent musculoskeletal pain: A randomized controlled trial in primary care
Avery 2016 [7]	Systematic development of a theory-informed multifaceted behavioural intervention to increase physical activity of adults with type 2 diabetes in routine primary care: Movement as Medicine for Type 2 Diabetes
Barrett 2017 [8]	Feasibility of a physical activity pathway for Irish primary care physiotherapy services
Bearne 2011 [9]	Feasibility of an exercise-based rehabilitation programme for chronic hip pain
Bierman 2001 [10]	Functional status, the sixth vital sign
Bickerdike 2017 [11]	Social prescribing: less rhetoric and more reality. A systematic review of the evidence
Bird 2019 [12]	General practice referral of 'at risk'populations to community leisure services: applying the RE-AIM framework to evaluate the impact of a community-based physical activity programme for inactive adults with long-term conditions
Bjerk 2017 [13]	A falls prevention programme to improve quality of life, physical function and falls efficacy in older people receiving home help services: Study protocol for a randomised controlled trial
Bjerre 2019 [14]	Community-based football in men with prostate cancer: 1-year follow-up on a pragmatic, multicentre randomised controlled trial
Boehler 2011 [15]	The cost of changing physical activity behaviour: evidence from a" physical activity pathway" in the primary care setting
Bossen 2013 [16]	Effectiveness of a web-based physical activity intervention in patients with knee and/or hip osteoarthritis: randomized controlled trial
Brannan 2019 [17]	Moving healthcare professionals–a whole system approach to embed physical activity in clinical practice
Bull 1995 [18]	Beliefs and behaviour of general practitioners regarding promotion of physical activity

Bull 2008 [19]	Evaluation of the Physical Activity Care Pathway London Feasibility Pilot– Final Technical Report
Bull and Milton 2010 [20]	A process evaluation of a" physical activity pathway" in the primary care setting.
Campbell 2015 [21]	A systematic review and economic evaluation of exercise referral schemes in primary care: a short report
Chaplin 2015 [22]	The evaluation of an interactive web-based Pulmonary Rehabilitation programme: protocol for the WEB SPACE for COPD feasibility study
Chatterjee 2017 [23]	GPs' knowledge, use, and confidence in national physical activity and health guidelines and tools: a questionnaire-based survey of general practice in England
Chong 2014 [24]	Physical activity program preferences and perspectives of older adults with and without cognitive impairment
Comer 2013 [25]	A Home Exercise Programme Is No More Beneficial than Advice and Education for People with Neurogenic Claudication: Results from a Randomised Controlled Trial
Coombes 2015 [26]	"Exercise is medicine": Curbing the burden of chronic disease and physical inactivity
Copeland 2019 [27]	Evaluation of the Public Health England and Sport England Funded Physical Activity Clinical Advice Pad Pilot
Coulter 2016 [28]	Personalised care planning for adults with chronic or long-term health conditions
Craike 2019 [29]	General practitioner referrals to exercise physiologists during routine practice: A prospective study
Croteau 2006 [30]	Physical activity advice in the primary care setting: results of a population study in New Zealand.
Dacey 2014 [31]	Physical activity counseling in medical school education: a systematic review
Daniellson 2016 [32]	Crawling Out of the Cocoon: Patients' Experiences of a Physical Therapy Exercise Intervention in the Treatment of Major Depression.
Dejonghe 2020 [33]	Health coaching for promoting physical activity in low back pain patients: a secondary analysis on the usage and acceptance
Devi 2014 [34]	A web-based program improves physical activity outcomes in a primary care angina population: Randomized controlled trial
Din 2015 [35]	Health professionals' perspectives on exercise referral from a process evaluation of the National Exercise Referral Scheme in Wales
Dunlop and Murray 2013 [36]	Major limitations in knowledge of physical activity guidelines among UK medical students revealed: implications for the undergraduate medical curriculum
Eakin 2008 [37]	The Logan Healthy Living Program: A cluster randomized trial of a telephone-delivered physical activity and dietary behavior intervention for primary care patients with type 2 diabetes or hypertension from a socially disadvantaged community - Rationale, design and recruitment
Eakin 2010a [38]	Living Well with Diabetes: a randomized controlled trial of a telephone-delivered intervention for maintenance of weight loss, physical activity and glycaemic control in adults with type 2 diabetes.

Eakin 2010b [39]	Maintenance of physical activity and dietary change following a telephone-delivered intervention
Ewald 2018 [40]	Physical activity coaching by Australian Exercise Physiologists is cost effective for patients referred from general practice
Fife-Schaw 2014 [41]	Comparing exercise interventions to increase persistence with physical exercise and sporting activity among people with hypertension or high normal blood pressure: Study protocol for a randomised controlled trial
Forsyth 2009 [42]	Dietitians and exercise physiologists in primary care: Lifestyle interventions for patients with depression and/or anxiety
Gamboa Moreno 2013 [43]	Impact of a self-care education programme on patients with type 2 diabetes in primary care in the Basque Country
Gamboa Moreno 2016 [44]	A Pilot Study to Assess the Feasibility of the Spanish Diabetes Self-Management Program in the Basque Country
Goode 2012 [45]	Telephone-delivered interventions for physical activity and dietary behavior change: an updated systematic review
Grant 2014 [46]	Exercise as a vital sign: A quasi-experimental analysis of a health system intervention to collect patient-reported exercise levels
Healey 2018 [47]	The feasibility and acceptability of a physical activity intervention for older people with chronic musculoskeletal pain: The iPOPP pilot trial protocol
Hinrichs 2011a [48]	General practitioner advice on physical activity: analyses in a cohort of older primary health care patients (getABI)
Hinrichs 2011b [49]	Effects of an exercise programme for chronically ill and mobility-restricted elderly with structured support by the general practitioner's practice (HOMEfit) - study protocol of a randomised controlled trial
Hinrichs 2016 [50]	Home-Based Exercise Supported by General Practitioner Practices: Ineffective in a Sample of Chronically Ill, Mobility-Limited Older Adults (the HOMEfit Randomized Controlled Trial)
Holden 2012 [51]	Role of exercise for knee pain: What do older adults in the community think?
Hurley 2018 [52]	Exercise interventions and patient beliefs for people with hip, knee or hip and knee osteoarthritis: A mixed methods review
Husk 2019 [53]	What approaches to social prescribing work, for whom, and in what circumstances? A realist review
James 2017 [54]	Referral for Expert Physical Activity Counseling: A Pragmatic RCT
Jansink 2010 [55]	Primary care nurses struggle with lifestyle counseling in diabetes care: a qualitative analysis
Jones 2018 [56]	Development of a physical literacy model for older adults–a consensus process by the collaborative working group on physical literacy for older Canadians
Jorgensen 2012 [57]	How do general practitioners in Denmark promote physical activity?
Kosteli 2017 [58]	Barriers and enablers of physical activity engagement for patients with COPD in primary care
Lamming 2017 [59]	What do we know about brief interventions for physical activity that could be delivered in primary care consultations? A systematic review of reviews

Leemrijse 2015 [60]	Collaboration of general practitioners and exercise providers in promotion of physical activity a written survey among general practitioners
Leenaars 2016 [61]	The role of the care sport connector in the Netherlands
Leijon 2008 [62]	Physical activity referrals in Swedish primary health care - Prescriber and patient characteristics, reasons for prescriptions, and prescribed activities
Lindeman 2020 [63]	The extent to which family physicians record their patients' exercise in medical records: a scoping review
Lion 2019 [64]	Physical activity promotion in primary care: a Utopian quest?
Lobelo 2009 [65]	Physical activity habits of doctors and medical students influence their counselling practices
Lohmann 2010 [66]	Fitness consultations in routine care of patients with type 2 diabetes in general practice: An 18-month non-randomised intervention study
Loughren 2014 [67]	'Let's Get Moving'Physical Activity Care Pathway (Gloucestershire) Post-Programme Evaluation Report.
Martin-Borras 2018 [68]	A new model of exercise referral scheme in primary care: is the effect on adherence to physical activity sustainable in the long term? A 15-month randomised controlled trial
McDonough 2013 [69]	Pedometer-driven walking for chronic low back pain: A feasibility randomized controlled trial
McKay 2001 [70]	The Diabetes Network Internet-Based Physical Activity Intervention A randomized pilot study.
Melillio 2000 [71]	Perceptions of nurse practitioners regarding their role in physical activity and exercise prescription for older adults
Moore 2013 [72]	Mixed-method process evaluation of the welsh national exercise referral scheme
Morgan 2015 [73]	Physical ACtivity facilitation for Elders (PACE): Study protocol for a randomised controlled trial
Morishita 2014 [74]	Primary care physicians' own exercise habits influence exercise counseling for patients with chronic kidney disease: A cross-sectional study
Faculty of Sport and Exercise Medicine UK 2018 [75]	'Moving Medicine'
Muellmann 2018 [76]	Effectiveness of eHealth interventions for the promotion of physical activity in older adults: A systematic review
Murphy 2012 [77]	An exploratory cluster randomised trial of a university halls of residence based social norms intervention in Wales, UK
NHS leading change [78]	Introducing group consultations for adults with Type 2 diabetes
NICE 2015 [79]	Dementia, disability and frailty in later life – mid-life approaches to delay or prevent onset.
NICE 2013 [80]	Physical activity: brief advice for adults in primary care
NICE 2014 [81]	Behaviour change: individual approaches
NICE 2014 [82]	Physical activity: exercise referral schemes.
NICE 2019 [83]	Making Every Contact Count: How NICE resources can support local priorities

Omura 2018 [84]	Primary care providers' physical activity counseling and referral practices and barriers for cardiovascular disease prevention
Parish 2006 [85]	Examination of the constructs of the Transtheoretical model in patients with heart failure: a focus on physical activity readiness.
Parkrun practice [86]	parkrun UK teams up with RCGP to 'prescribe' active lifestyles to patients and practice staff
Persson 2013 [87]	Physical activity on prescription (PAP) from the general practitioner's perspective–a qualitative study
Pescheny 2018 [88]	Facilitators and barriers of implementing and delivering social prescribing services: a systematic review
Prochaska 2000 [89]	PACE Interactive Communication Technology for Behavior Change in Clinical Settings
Department of Health 2012 [90]	Let's Get Moving commissioning guidance
Quirk and Haarke 2019 [91]	How can we get more people with long-term health conditions involved in parkrun? A qualitative study evaluating parkrun's PROVE project
Rhodes 2020 [92]	Increasing physical activity by four legs rather than two: systematic review of dog-facilitated physical activity interventions
Royal College of General Practitioners [93]	Active Practice' Website
Rushforth 2016 [94]	Barriers to effective management of type 2 diabetes in primary care: qualitative systematic review
Savill 2015 [95]	Is general practice engaged with physical activity promotion?
Schofield 2005 [96]	Trust levels of physical activity information sources: a population study
Shaw 2012 [97]	Exercise for overweight or obesity
Short 2016 [98]	Physical activity recommendations from general practitioners in Australia. Results from a national survey
Smith 2016 [99]	Interventions for improving outcomes in patients with multimorbidity in primary care and community settings
Smith 2019 [100]	Social prescribing programmes to prevent or delay frailty in community-dwelling older adults
Stone 2015 [101]	Painful choices: a qualitative exploration of facilitators and barriers to active lifestyles among adults with osteoarthritis
Sturgiss 2016 [102]	Increasing general practitioners' confidence and self-efficacy in managing obesity: a mixed methods study
UK Chief Medical Officers 2011 [103]	Start Active, Stay Active: A report on physical activity from the four home countries' Chief Medical Officers (now updated, see below)
UK Chief Medical Officers 2019 [104]	Physical activity guidelines: UK Chief Medical Officers' report
Physical Activity Guidelines for Americans [105]	Physical Activity Guidelines for Americans
Van der Wulp 2012 [106]	Effectiveness of peer-led self-management coaching for patients recently diagnosed with Type 2 diabetes mellitus in primary care: A randomized controlled trial

Val Slujis 2005 [107]	Effect of a tailored physical activity intervention delivered in general practice settings: results of a randomized controlled trial
Van Slujis 2005 [108]	The positive effect on determinants of physical activity of a tailored, general practice-based physical activity intervention
Vanroy 2017 [109]	Short- and long-term effects of a need-supportive physical activity intervention among patients with type 2 diabetes mellitus: A randomized controlled pilot trial
Verwey 2014 [110]	A pilot study of a tool to stimulate physical activity in patients with COPD or type 2 diabetes in primary care
Verwey 2014 [111]	A monitoring and feedback tool embedded in a counselling protocol to increase physical activity of patients with COPD or type 2 diabetes in primary care: Study protocol of a three-arm cluster randomised controlled trial
Verwey 2016 [112]	Upgrading physical activity counselling in primary care in the Netherlands, Oxford University Press.
Verwey 2016 [113]	Process evaluation of physical activity counselling with and without the use of mobile technology: A mixed methods study
Walsh 1999 [114]	Exercise Counseling by Primary Care Physicians in the Era of Managed Care.
Ward 2015 [115]	A Survey of Physical Activity in MedicalCurricula: A report of the HEPA in Health Care SettingsHEPA Europe Working Group
Weiler 2012 [116]	Physical activity education in the undergraduate curricula of all UK medical schools. Are tomorrow's doctors equipped to follow clinical guidelines?
Weinstock 2011 [117]	Lessened decline in physical activity and impairment of older adults with diabetes with telemedicine and pedometer use: results from the IDEATel study." Age and Ageing 40(1): 98-105.
Wheeler 2019 [118]	Primary care knowledge and beliefs about physical activity and health: a survey of primary healthcare team members
Wilcox 2010 [119]	Adoption and Implementation of Physical Activity and Dietary Counseling by Community Health Center Providers and Nurses.
Williams 2020 [120]	Translating a walking intervention for health professional delivery within primary care: A mixed-methods treatment fidelity assessment.
Wormald and Ingle 2004 [121]	Hull and East Riding Primary Care Trusts, Hull. 2 Lecturer in Exercise Physiology.

Full reference list

1. Allen, K.D., et al., *Patient, provider, and combined interventions for managing osteoarthritis in primary care: A cluster randomized trial.* Annals of Internal Medicine, 2017. **166**(6): p. 401-411.
2. Allen, K.D., et al., *Patient and provider interventions for managing osteoarthritis in primary care: protocols for two randomized controlled trials*, 2012.
3. Andryukhin, A., et al., *The impact of a nurse-led care programme on events and physical and psychosocial parameters in patients with heart failure with preserved ejection fraction: A randomized clinical trial in primary care in Russia.* European Journal of General Practice, 2010. **16**(4): p. 205-214.

4. Arden, K., F. Fatoye, and G. Yeowell, *Evaluation of a rolling rehabilitation programme for patients with non-specific low back pain in primary care: an observational cohort study.* Journal of Evaluation in Clinical Practice, 2017. **23**(2): p. 272-278.
5. Åsenlöf, P., E. Denison, and P. Lindberg, *Individually tailored treatment targeting activity, motor behavior, and cognition reduces pain-related disability: A randomized controlled trial in patients with musculoskeletal pain.* Journal of Pain, 2005. **6**(9): p. 588-603.
6. Åsenlöf, P., E. Denison, and P. Lindberg, *Long-term follow-up of tailored behavioural treatment and exercise based physical therapy in persistent musculoskeletal pain: A randomized controlled trial in primary care.* European Journal of Pain, 2009. **13**(10): p. 1080-1088.
7. Avery, L., et al., *Systematic development of a theory-informed multifaceted behavioural intervention to increase physical activity of adults with type 2 diabetes in routine primary care: Movement as Medicine for Type 2 Diabetes.* Implement Sci, 2016. **11**(1).
8. Barrett, E.M., J. Hussey, and C.D. Darker, *Feasibility of a physical activity pathway for Irish primary care physiotherapy services.* Physiotherapy, 2017. **103**(1): p. 106-112.
9. Bearne, L.M., et al., *Feasibility of an exercise-based rehabilitation programme for chronic hip pain.* Musculoskeletal Care, 2011. **9**(3): p. 160-168.
10. Bierman, A.S., *Functional status, the sixth vital sign.* Journal of General Internal Medicine, 2001(16): p. 785-786.
11. Bickerdike, L., et al., *Social prescribing: less rhetoric and more reality. A systematic review of the evidence.* BMJ open, 2017. **7**(4): p. e013384.
12. Bird, E.L., M.S.Y. Biddle, and J.E. Powell, *General practice referral of 'at risk'populations to community leisure services: applying the RE-AIM framework to evaluate the impact of a community-based physical activity programme for inactive adults with long-term conditions.* BMC public health, 2019. **19**(1): p. 1308.
13. Bjerk, M., et al., *A falls prevention programme to improve quality of life, physical function and falls efficacy in older people receiving home help services: Study protocol for a randomised controlled trial.* BMC Health Services Research, 2017. **17**(1).
14. Bjerre, E.D., et al., *Community-based football in men with prostate cancer: 1-year follow-up on a pragmatic, multicentre randomised controlled trial.* PLoS medicine, 2019. **16**(10).
15. Boehler, C., et al., *The cost of changing physical activity behaviour: evidence from a "physical activity pathway" in the primary care setting.* BMC Public Health, 2011. **11**(1): p. 370-370.
16. Bossen, D., et al., *Effectiveness of a web-based physical activity intervention in patients with knee and/or hip osteoarthritis: randomized controlled trial.* Journal of medical Internet research, 2013. **15**(11).
17. Brannan, M., et al., *Moving healthcare professionals–a whole system approach to embed physical activity in clinical practice.* BMC Med Educ, 2019. **19**(1): p. 84.
18. Bull, F.C.L., et al., *Beliefs and behaviour of general practitioners regarding promotion of physical activity.* Australian Journal of Public Health, 1995. **19**(3): p. 300-304.
19. Bull, F., K. Milton, and C. Boehler, *Evaluation of the Physical Activity Care Pathway London Feasibility Pilot–Final Technical Report*, 2008: https://assets.publishing.service.gov.uk/government/uploads/system/uploads/attachment_data/file/192041/Evaluation_of_the_Physical_Activity_Care_Pathway_London_Feasibility_Pilot_-_Report.pdf.
20. Bull, F.C. and K.E. Milton, *A process evaluation of a" physical activity pathway" in the primary care setting.* BMC Public Health, 2010. **10**(1): p. 463.
21. Campbell, F., et al., *A systematic review and economic evaluation of exercise referral schemes in primary care: a short report.* Health Technol Assess, 2015. **19**(60).
22. Chaplin, E., et al., *The evaluation of an interactive web-based Pulmonary Rehabilitation programme: protocol for the WEB SPACE for COPD feasibility study.* BMJ open, 2015. **5**(8): p. e008055.

23. Chatterjee, R., et al., *GPs' knowledge, use, and confidence in national physical activity and health guidelines and tools: a questionnaire-based survey of general practice in England.* Br J Gen Pract, 2017. **67**(663): p. e668-e675.
24. Chong, T.W.H., et al., *Physical activity program preferences and perspectives of older adults with and without cognitive impairment.* Asia-Pacific Psychiatry, 2014. **6**(2): p. 179-190.
25. Comer, C., et al., *A Home Exercise Programme Is No More Beneficial than Advice and Education for People with Neurogenic Claudication: Results from a Randomised Controlled Trial.* PLoS ONE, 2013. **8**(9).
26. Coombes, J.S., et al., *"Exercise is medicine": Curbing the burden of chronic disease and physical inactivity.* Asia-Pacific Journal of Public Health, 2015. **27**(2): p. NP600-NP605.
27. Copeland, R., et al., *Evaluation of the Public Health England and Sport England Funded Physical Activity Clinical Advice Pad Pilot*, 2019: Sheffield: National Centre for Sport and Exercise Medicine.
28. Coulter, A., et al., *Personalised care planning for adults with chronic or long-term health conditions.* Cochrane Database Syst Rev, 2015. **3**(3).
29. Craike, M., et al., *General practitioner referrals to exercise physiologists during routine practice: A prospective study.* Journal of science and medicine in sport, 2019. **22**(4): p. 478-483.
30. Croteau, K., G. Schofield, and G. McLean, *Physical activity advice in the primary care setting: results of a population study in New Zealand.* Australian and New Zealand journal of public health, 2006. **30**(3): p. 262-267.
31. Dacey, M.L., et al., *Physical activity counseling in medical school education: a systematic review.* Medical education online, 2014. **19**(1): p. 24325.
32. Danielsson, L., B. Kihlbom, and S. Rosberg, *"Crawling Out of the Cocoon": Patients' Experiences of a Physical Therapy Exercise Intervention in the Treatment of Major Depression*, 2016.
33. Dejonghe, L.A.L., et al., *Health coaching for promoting physical activity in low back pain patients: a secondary analysis on the usage and acceptance.* BMC Sports Science, Medicine and Rehabilitation, 2020. **12**(1): p. 2.
34. Devi, R., J. Powell, and S. Singh, *A web-based program improves physical activity outcomes in a primary care angina population: Randomized controlled trial.* Journal of Medical Internet Research, 2014. **16**(9).
35. Din, N.U., et al., *Health professionals' perspectives on exercise referral from a process evaluation of the National Exercise Referral Scheme in Wales.* Health Education J 2015. **74**(6): p. 743-757.
36. Dunlop, M. and A.D. Murray, *Major limitations in knowledge of physical activity guidelines among UK medical students revealed: implications for the undergraduate medical curriculum.* Br J Sports Med, 2013. **47**(11): p. 718-720.
37. Eakin, E.G., et al., *The Logan Healthy Living Program: A cluster randomized trial of a telephone-delivered physical activity and dietary behavior intervention for primary care patients with type 2 diabetes or hypertension from a socially disadvantaged community - Rationale, design and recruitment.* Contemporary Clinical Trials, 2008. **29**(3): p. 439-454.
38. Eakin, E.G., et al., *Living Well with Diabetes: a randomized controlled trial of a telephone-delivered intervention for maintenance of weight loss, physical activity and glycaemic control in adults with type 2 diabetes*, 2010.
39. Eakin, E., et al., *Maintenance of physical activity and dietary change following a telephone-delivered intervention.* Health Psychology, 2010. **29**(6): p. 566-573.
40. Ewald, B., et al., *Physical activity coaching by Australian Exercise Physiologists is cost effective for patients referred from general practice.* Aust N Z J Public Health, 2018. **42**(1): p. 12-15.

41. Fife-Schaw, C., et al., *Comparing exercise interventions to increase persistence with physical exercise and sporting activity among people with hypertension or high normal blood pressure: Study protocol for a randomised controlled trial.* Trials, 2014. **15**(1).
42. Forsyth, A., F.P. Deane, and P. Williams, *Dietitians and exercise physiologists in primary care: Lifestyle interventions for patients with depression and/or anxiety.* Journal of Allied Health, 2009.
43. Gamboa Moreno, E., et al., *Impact of a self-care education programme on patients with type 2 diabetes in primary care in the Basque Country.* BMC Public Health, 2013. **13**(1).
44. Gamboa Moreno, E., et al., *A Pilot Study to Assess the Feasibility of the Spanish Diabetes Self-Management Program in the Basque Country.* Journal of diabetes research, 2016. **2016**: p. 9145673-9145673.
45. Goode, A.D., M.M. Reeves, and E.G. Eakin, *Telephone-delivered interventions for physical activity and dietary behavior change: an updated systematic review.* Am J Prev Med, 2012. **42**(1): p. 81-88.
46. Grant, R.W., et al., *Exercise as a vital sign: A quasi-experimental analysis of a health system intervention to collect patient-reported exercise levels.* Journal of General Internal Medicine, 2014. **29**(2): p. 341-348.
47. Healey, E.L., et al., *The feasibility and acceptability of a physical activity intervention for older people with chronic musculoskeletal pain: The iPOPP pilot trial protocol.* Musculoskeletal Care, 2018. **16**(1): p. 118-132.
48. Hinrichs, T., et al., *General practitioner advice on physical activity: analyses in a cohort of older primary health care patients (getABI).* BMC family practice, 2011. **12**(1): p. 26.
49. Hinrichs, T., et al., *Effects of an exercise programme for chronically ill and mobility-restricted elderly with structured support by the general practitioner's practice (HOMEfit) - study protocol of a randomised controlled trial.* Trials, 2011. **12**.
50. Hinrichs, T., et al., *Home-Based Exercise Supported by General Practitioner Practices: Ineffective in a Sample of Chronically Ill, Mobility-Limited Older Adults (the HOMEfit Randomized Controlled Trial).* Journal of the American Geriatrics Society, 2016. **64**(11): p. 2270-2279.
51. Holden, M.A., et al., *Role of exercise for knee pain: What do older adults in the community think?* Arthritis Care and Research, 2012. **64**(10): p. 1554-1564.
52. Hurley, M., et al., *Exercise interventions and patient beliefs for people with hip, knee or hip and knee osteoarthritis: A mixed methods review*, 2018, John Wiley and Sons Ltd.
53. Husk, K., et al., *What approaches to social prescribing work, for whom, and in what circumstances? A realist review.* Health & social care in the community., 2019.
54. James, E.L., et al., *Referral for Expert Physical Activity Counseling: A Pragmatic RCT.* American Journal of Preventive Medicine 2017. **53**(4): p. 490–499.
55. Jansink, R., et al., *Primary care nurses struggle with lifestyle counseling in diabetes care: a qualitative analysis.* BMC family practice, 2010. **11**(1): p. 41.
56. Jones, G.R., et al., *Development of a physical literacy model for older adults–a consensus process by the collaborative working group on physical literacy for older Canadians.* 2018.
57. Jorgensen, T.K., M. Nordentoft, and J. Krogh, *How do general practitioners in Denmark promote physical activity?* Scandinavian Journal of Primary Health Care, 2012. **30**(3): p. 141-146.
58. Kosteli, M.C., et al., *Barriers and enablers of physical activity engagement for patients with COPD in primary care.* International Journal of COPD, 2017. **12**: p. 1019-1031.
59. Lamming, L., et al., *What do we know about brief interventions for physical activity that could be delivered in primary care consultations? A systematic review of reviews.* Prev Med, 2017. **99**: p. 152-163.

60. Leemrijse, C.J., et al., *Collaboration of general practitioners and exercise providers in promotion of physical activity a written survey among general practitioners.* BMC Family Practice, 2015. **16**(1): p. 1-9.
61. Leenaars, K.E., et al., *The role of the care sport connector in the Netherlands.* Health Promotion International, 2016.
62. Leijon, M.E., et al., *Physical activity referrals in Swedish primary health care - Prescriber and patient characteristics, reasons for prescriptions, and prescribed activities.* BMC Health Services Research, 2008. **8**.
63. Lindeman, C., et al., *The extent to which family physicians record their patients' exercise in medical records: a scoping review.* BMJ open, 2020. **10**(2).
64. Lion, A., et al., *Physical activity promotion in primary care: a Utopian quest?* Health promotion international, 2019. **34**(4): p. 877-886.
65. Lobelo, F., J. Duperly, and E. Frank, *Physical activity habits of doctors and medical students influence their counselling practices.* Br J Sports Med online, 2009. **43**(2): p. 89-92.
66. Lohmann, H., V. Siersma, and N.F. Olivarius, *Fitness consultations in routine care of patients with type 2 diabetes in general practice: An 18-month non-randomised intervention study.* BMC Family Practice, 2010. **11**.
67. Loughren, E.A., C. Baker, and D. Crone, *'Let's Get Moving'Physical Activity Care Pathway (Gloucestershire) Post-Programme Evaluation Report.*, 2014: http://eprints.glos.ac.uk/2378/.
68. Martín-Borràs, C., et al., *A new model of exercise referral scheme in primary care: is the effect on adherence to physical activity sustainable in the long term? A 15-month randomised controlled trial.* BMJ Open, 2018. **8**(3): p. e017211-e017211.
69. McDonough, S.M., et al., *Pedometer-driven walking for chronic low back pain: A feasibility randomized controlled trial.* Clinical Journal of Pain, 2013. **29**(11): p. 972-981.
70. McKay, H.G., et al., *The Diabetes Network Internet-Based Physical Activity Intervention A randomized pilot study*, 2001.
71. Melillo, K.D., et al., *Perceptions of nurse practitioners regarding their role in physical activity and exercise prescription for older adults.* Clinical excellence for nurse practitioners : the international journal of NPACE, 2000. **4**(2): p. 108-116.
72. Moore, G.F., et al., *Mixed-method process evaluation of the welsh national exercise referral scheme.* Health Education 2013.
73. Morgan, G.S., et al., *Physical ACtivity facilitation for Elders (PACE): Study protocol for a randomised controlled trial.* Trials, 2015. **16**(1).
74. Morishita, Y., et al., *Primary care physicians' own exercise habits influence exercise counseling for patients with chronic kidney disease: A cross-sectional study.* BMC Nephrology, 2014. **15**(1).
75. Faculty of Sport and Exercise Medicine. *Moving Medicine*. 2018.
76. Muellmann, S., et al., *Effectiveness of eHealth interventions for the promotion of physical activity in older adults: A systematic review.* Prev Med, 2018. **108**: p. 93-110.
77. Murphy, S., et al., *An exploratory cluster randomised trial of a university halls of residence based social norms intervention in Wales, UK.* BMC Public Health, 2012. **12**(186): p. 1471-2458.
78. NHS Leading Change Adding Value Team. *Introducing group consultations for adults with Type 2 diabetes*. 2019 2020].
79. National Institute for Clinical Excellence, *Dementia, disability and frailty in later life – mid-life approaches to delay or prevent onset*, 2015.
80. National Institute for Clinical Excellence, *Physical activity: brief advice for adults in primary care.* London: Nice public health guidance 2013: p. 44.
81. National Institute for Clinical Excellence, *Behaviour change: individual approaches*, 2014.
82. National Institute for Clinical Excellence, *Physical activity: exercise referral schemes*, 2014.

83. National Institute for Clinical Excellence. *Making Every Contact Count: How NICE resources can support local priorities*. [cited 2019; Available from: https://stpsupport.nice.org.uk/mecc/index.html.
84. Omura, J.D., et al., *Primary care providers' physical activity counseling and referral practices and barriers for cardiovascular disease prevention.* Preventive Medicine, 2018. **108**: p. 115-122.
85. Rena Parish, T. and B.B.S. Tracie Rena Parish, *Examination of the constructs of the Transtheoretical model in patients with heart failure: a focus on physical activity readiness*, 2006.
86. RCGP and Parkrun. *parkrun UK teams up with RCGP to 'prescribe' active lifestyles to patients and practice staff* 21st June 2018 [cited 2019.
87. Persson, G., et al., *Physical activity on prescription (PAP) from the general practitioner's perspective–a qualitative study.* BMC family practice, 2013. **14**(1): p. 128.
88. Pescheny, J.V., Y. Pappas, and G. Randhawa, *Facilitators and barriers of implementing and delivering social prescribing services: a systematic review.* BMC health services research, 2018. **18**(1): p. 86.
89. Prochaska, J.J., et al., *PACE Interactive Communication Technology for Behavior Change in Clinical Settings*, 2000. p. 127-131.
90. Department of Health. *Resources for commissioning Let's Get Moving interventions* 2012 05/04/2020].
91. Quirk, H. and S. Haake, *How can we get more people with long-term health conditions involved in parkrun? A qualitative study evaluating parkrun's PROVE project.* BMC Sports Sci Med Rehabil, 2019. **11**(1): p. 22.
92. Rhodes, R.E., et al., *Increasing physical activity by four legs rather than two: systematic review of dog-facilitated physical activity interventions.* British Journal of Sports Medicine, 2020.
93. Royal College of General Practitioners. *RCGP Active Practice Charter*. 2019 [cited 2019; Available from: https://r1.dotdigital-pages.com/p/49LX-5IR/active-practice-charter.
94. Rushforth, B., et al., *Barriers to effective management of type 2 diabetes in primary care: qualitative systematic review.* Br J Gen Pract, 2016. **66**(643): p. e114-e127.
95. Savill, B., A. Murray, and R. Weiler, *Is general practice engaged with physical activity promotion?* Br J Gen Pract, 2015. **65**(638): p. 484-485.
96. Schofield, G., K. Croteau, and G. McLean, *Trust levels of physical activity information sources: a population study.* Health Promotion Journal of Australia, 2005. **16**(3): p. 221-224.
97. Shaw, R., et al., *Pre-exercise screening and health coaching in CHD secondary prevention: A qualitative study of the patient experience.* Health Education Research, 2012. **27**(3): p. 424-436.
98. Short, C.E., et al., *Physical activity recommendations from general practitioners in Australia. Results from a national survey.* Australian and New Zealand Journal of Public Health, 2016. **40**(1): p. 83-90.
99. Smith, S.M., et al., *Interventions for improving outcomes in patients with multimorbidity in primary care and community settings.* Cochrane Database of Systematic Reviews, 2016(3).
100. Smith, T.O., et al., *Social prescribing programmes to prevent or delay frailty in community-dwelling older adults.* Geriatrics, 2019. **4**(4): p. 65.
101. Stone, R.C. and J. Baker, *Painful choices: a qualitative exploration of facilitators and barriers to active lifestyles among adults with osteoarthritis.* Journal of Applied Gerontology, 2015. **36**(9): p. 1091-1116.
102. Sturgiss, E., et al., *Increasing general practitioners' confidence and self-efficacy in managing obesity: a mixed methods study* BMJ open 2017. **7**(1): p. e014314.
103. Department of Health, P.A., Health Improvement and Protection, *Start Active, Stay Active: A report on physical activity from the four home countries' Chief Medical Officers (Now*

updated), 2011: https://assets.publishing.service.gov.uk/government/uploads/system/uploads/attachment_data/file/830943/withdrawn_dh_128210.pdf.
104. UK CMOs, *Physical activity guidelines: UK chief medical officers' report*, 2019: https://www.gov.uk/government/publications/physical-activity-guidelines-uk-chief-medical-officers-report.
105. US Department of Health and Human Services, *Physical Activity Guidelines for Americans, 2nd edition*, 2018: Washington, DC.
106. Van der Wulp, I., et al., *Effectiveness of peer-led self-management coaching for patients recently diagnosed with Type 2 diabetes mellitus in primary care: A randomized controlled trial.* Diabetic Medicine, 2012. **29**(10).
107. van Sluijs, E.M.F., et al., *Effect of a tailored physical activity intervention delivered in general practice settings: results of a randomized controlled trial.* American Journal of Public Health, 2005. **95**(10): p. 1825-1831.
108. Van Sluijs, E.M.F., et al., *The positive effect on determinants of physical activity of a tailored, general practice-based physical activity intervention.* Health education research, 2005. **20**(3): p. 345-356.
109. Vanroy, J., et al., *Short- and long-term effects of a need-supportive physical activity intervention among patients with type 2 diabetes mellitus: A randomized controlled pilot trial.* PLoS ONE, 2017. **12**(4).
110. Verwey, R., et al., *A pilot study of a tool to stimulate physical activity in patients with COPD or type 2 diabetes in primary care.* Journal of Telemedicine and Telecare, 2014. **20**(1): p. 29-34.
111. Verwey, R., et al., *A monitoring and feedback tool embedded in a counselling protocol to increase physical activity of patients with COPD or type 2 diabetes in primary care: Study protocol of a three-arm cluster randomised controlled trial.* BMC Family Practice, 2014. **15**(1).
112. Verwey, R.D.S.e., et al. *Upgrading physical activity counselling in primary care in the Netherlands*. Oxford University Press.
113. Verwey, R., et al., *Process evaluation of physical activity counselling with and without the use of mobile technology: A mixed methods study.* International Journal of Nursing Studies, 2016. **53**: p. 3-16.
114. Walsh, J.M.E., et al., *Exercise Counseling by Primary Care Physicians in the Era of Managed Care*, 1999.
115. Ward, M., *A Survey of Physical Activity in MedicalCurricula: A report of the HEPA in Health Care Settings*, 2015: HEPA Europe Working Group.
116. Weiler, R., et al., *Physical activity education in the undergraduate curricula of all UK medical schools. Are tomorrow's doctors equipped to follow clinical guidelines?* British journal of sports medicine, 2012. **46**(14): p. 1024-1026.
117. Weinstock, R.S., et al., *Lessened decline in physical activity and impairment of older adults with diabetes with telemedicine and pedometer use: results from the IDEATel study.* Age and Ageing, 2011. **40**(1): p. 98-105.
118. Wheeler, P.C., et al., *Primary care knowledge and beliefs about physical activity and health: a survey of primary healthcare team members.* BJGP open, 2017. **1**(2).
119. Wilcox, S., et al., *Adoption and Implementation of Physical Activity and Dietary Counseling by Community Health Center Providers and Nurses*, 2004.
120. Williams, S.L., et al., *Translating a walking intervention for health professional delivery within primary care: A mixed-methods treatment fidelity assessment.* Br J Health Psychol, 2019. **25**(1): p. 17-38.
121. Wormald, H. and L. Ingle, *GP exercise referral schemes: Improving the patient's experience.* Education Journal,, 2004. **63**(4): p. 362-373
