# Supplementary material for: ‘Function First’: how to promote physical activity and physical function in people with long-term conditions managed in primary care? A study combining realist and co-design methods
Source: BMJ Open. 2021 Jul 27;11(7):e046751. doi: 10.1136/bmjopen-2020-046751 (PMC8317101; doi:10.1136/bmjopen-2020-046751)
Supplement: Supplementary data [file bmjopen-2020-046751supp007.pdf]

Supplementary table 5: Taxonomy of primary care physical activity interventions for people with long-term conditions

**Type of intervention**	**Description**	**Example papers**
Brief interventions	Brief verbal advice, discussion, negotiation or encouragement, with or without written or other support or follow-up. It can vary from basic advice to a more extended, individually focused discussion	**Lamming (2017) – systematic review of reviews [1]**<br>Brief Interventions can increase self-reported physical activity in the short term, but there is insufficient evidence about their long-term impact, their impact on objectively measured physical activity, and about the factors that influence their effectiveness, feasibility and acceptability.
Telephone interventions	Physical activity interventions delivered predominantly over the telephone	**Goode (2012) – systematic review [2]**<br>Findings provide continuing strong evidence supporting the efficacy of telephone-delivered interventions to promote physical activity.
Online/'eHealth' interventions	The use of information and communication technologies for health"(WHO, 2015).	**Muellmann (2018) – systematic review [3]**<br>'eHealth' interventions can effectively promote PA in older adults aged 55 years and above in the short-term, while evidence regarding long-term effects and the added benefit of eHealth compared to non-eHealth intervention components is still lacking.
Exercise referral schemes	The practice of referring a person from primary care to a qualified exercise professional who uses relevant medical information about the person to develop a tailored programme of physical activity usually lasting from 10 to 12 weeks.	**Campbell (2015) – systematic review [4]**<br>Compared with usual care, exercise referral schemes had a small effect in increasing the proportion of individuals achieving 90–150 min of at least moderate intensity activity per week.
Community 'navigators'	Appropriately trained individuals who can guide patients towards local physical activity opportunities.	**Leenaars 2017 – Care Sport Connectors (CSCs)[5]**<br>Introduced in 2012 in the Netherlands and seem to hold the promise of improving collaboration between the primary care and the physical activity sector, especially because the roles that CSCs perceive themselves as having seem to be directed at eliminating barriers in this connection.
Referral to exercise specialists (e.g. exercise physiologists)	Referral to a qualified exercise physiologist from primary care (implemented in Australia)	**Ewald 2018 – Randomised controlled trial to establish cost-effectiveness [6]**<br>Referral to an exercise physiologist increased physical activity, which persisted at 9 month follow-up. Coaching achieved a modest increase in activity equivalent to 10 minutes walking per day, at a cost of AUD$245

		(approx. £150) per person. Face-to-face and telephone counselling were both effective. Note: a change in quality of life not observed so could not estimate utility.
Intervention delivery by existing primary care staff	When primary care professionals (e.g. practice nurses, healthcare assistants) deliver physical activity interventions	**Williams et al (2019) – A mixed-methods treatment fidelity assessment [7]**<br>Two practice nurses and six health care assistants delivered a theory-based walking intervention to 63 patients in their own practices. High levels of fidelity of delivery were demonstrated. However, patient-, provider-, and component-level factors impacted on treatment delivery and receipt.
Physical activity 'pathways'	'Let's Get Moving'; a UK-based attempt at embedding physical activity promotion into routine primary care, recommended by Public Health England for commissioning at a local level by Primary Care Trusts within NHS England	**Bull 2008, 2010 – Feasibility pilot study and process evaluation [8, 9]**<br>• Recruitment low, particularly in practices recruiting opportunistically vs. disease register<br>• Multiple promotion strategies needed<br>• Active patients did not move through 'exit' pathway as intended<br>• Professionals modified intervention to meet time available<br>• Needs better integration with existing referral pathways (e.g. ERS)<br>• Inventory of local opportunities would be helpful<br>**Boehler 2011 – 'the costs of changing physical activity behaviour' [10]**<br>Disease register screening is more costly than opportunistic patient recruitment (£53 vs £191). However, additional costs come with a higher completion rate and better outcomes in terms of behavioural change in patients completing the care pathway.<br>**Department of Health commissioning guidance (2012) –** includes resources and protocols for implementation [11]<br>**Loughren, 2014 – 'Let's Get Moving' Physical Activity Care Pathway (Gloucestershire) [12]**<br>Factors determining implementation success:<br>• Time required to deliver lifestyle counselling<br>• Integration/competition with existing ERS<br>• On-going support for deliverers in coordination<br>**Barrett 2017 – Feasibility in primary care physiotherapy [13]**<br>Concluded to be a clinically feasible resource to primary care physiotherapists with some

		modifications and with the support of additional resources (i.e. professional training and integration with existing schemes)
Practice-wide initiatives	Active Practice Charter promoted by the Royal College of General Practitioners	**Active Practice Charter (2019)** [14]<br>The Royal College of GPs and Sport England have launched the Active Practice Charter to inspire and celebrate GP practices that are taking steps to increase activity and reduce sedentary behaviour in their patients and staff.
Community initiatives adopted by primary care	*Parkrun* practice	**Quirk and Haarke (2019) [15]** - *Parkrun* launched a project called PROVE in 2016 to engage people living with long-term health conditions in England. Over the 3 year project, *Parkrun* appointed volunteer outreach ambassadors with a specialist interest in the health condition they represented whose role was to ensure *parkrun* was welcoming, supportive and inclusive. A qualitative evaluation showed that PROVE was regarded by the ambassadors (patients, carers, professionals) as important for ensuring that people with long-term health conditions can engage in physical activity and volunteering in a safe and supportive environment.
A whole system approach to embed physical activity in clinical practice	Moving healthcare professionals programme (MHPP)	**Brannan et al (2019) [16] –** The MHPP model is a partnership between Public Health England and Sport England. It has delivered face-to-face training to 17,105 healthcare professionals, embedded materials in almost three quarters of medical schools in England and overseen > 95,000 e-learning modules over two and half years. The programme aims to bring about improvements in knowledge, skills and practice. Individual elements of the model are being evaluated and further evaluation is planned to assess patient impact.
	Moving Medicine	**'Moving Medicine' [17]** was created to aid healthcare workers in integrating conversations about physical activity during routine clinical care. Moving medicine offers this online support for all patients of all ages and conditions. It also offers advice depending on the amount of time available with a patient and adjusts the conversations to these needs. Moving Medicine has the option of completing an 'online physical activity training course, which is accredited with 16 CPD points from the Faculty of Sport and Exercise Medicine (UK).

Multi-faceted interventions	Interventions involving a combination of approaches	**Avery et al (2016) [18] –** 'Movement as Medicine for Type 2 diabetes' is a multifaceted intervention informed by the theory of planned behaviour and social cognitive theory and consisted of 15 behaviour change techniques. It includes an accredited online training programme for healthcare professionals who deliver a behavioural intervention for adults with type 2 diabetes. This. Intervention intensity and duration were informed by a systematic review and stakeholder work. Usability testing resolved technical problems with using the online training intervention on practice IT systems. An open pilot study of the intervention identified mechanisms to enhance intervention implementation during routine diabetes consultations.
**Campaigns**	Age UK Resources – 'We Are Undefeatable'	The '**We Are Undefeatable' [19]** campaign was developed by 15 leading health and social care charities. Its main aim is to support and encourage ways for individuals with a wide range of health conditions, to remain active even during times where this can be challenging.

Reference list

1. Lamming, L., et al., *What do we know about brief interventions for physical activity that could be delivered in primary care consultations? A systematic review of reviews.* Prev Med, 2017. **99**: p. 152-163.
2. Goode, A.D., M.M. Reeves, and E.G. Eakin, *Telephone-delivered interventions for physical activity and dietary behavior change: an updated systematic review.* Am J Prev Med, 2012. **42**(1): p. 81-88.
3. Muellmann, S., et al., *Effectiveness of eHealth interventions for the promotion of physical activity in older adults: A systematic review.* Prev Med, 2018. **108**: p. 93-110.
4. Campbell, F., et al., *A systematic review and economic evaluation of exercise referral schemes in primary care: a short report.* Health Technol Assess, 2015. **19**(60).
5. Leenaars, K.E., et al., *Exploring the impact of the care sport connector in the Netherlands.* BMC public health, 2017. **17**(1): p. 813.
6. Ewald, B., et al., *Physical activity coaching by Australian Exercise Physiologists is cost effective for patients referred from general practice.* Aust N Z J Public Health, 2018. **42**(1): p. 12-15.
7. Williams, S.L., et al., *Translating a walking intervention for health professional delivery within primary care: A mixed-methods treatment fidelity assessment.* Br J Health Psychol, 2019. **25**(1): p. 17-38.
8. Bull, F., K. Milton, and C. Boehler, *Evaluation of the Physical Activity Care Pathway London Feasibility Pilot–Final Technical Report*, 2008: https://assets.publishing.service.gov.uk/government/uploads/system/uploads/attachment_

data/file/192041/Evaluation_of_the_Physical_Activity_Care_Pathway_London_Feasibility_Pilot_-_Report.pdf.
9. Bull, F.C. and K.E. Milton, *A process evaluation of a" physical activity pathway" in the primary care setting.* BMC Public Health, 2010. **10**(1): p. 463.
10. Boehler, C., et al., *The cost of changing physical activity behaviour: evidence from a "physical activity pathway" in the primary care setting.* BMC Public Health, 2011. **11**(1): p. 370-370.
11. Department of Health. *Resources for commissioning Let's Get Moving interventions* 2012 05/04/2020].
12. Loughren, E.A., C. Baker, and D. Crone, *'Let's Get Moving'Physical Activity Care Pathway (Gloucestershire) Post-Programme Evaluation Report.*, 2014: http://eprints.glos.ac.uk/2378/.
13. Barrett, E.M., J. Hussey, and C.D. Darker, *Feasibility of a physical activity pathway for Irish primary care physiotherapy services.* Physiotherapy, 2017. **103**(1): p. 106-112.
14. Royal College of General Practitioners. *RCGP Active Practice Charter*. 2019 [cited 2019; Available from: https://r1.dotdigital-pages.com/p/49LX-5IR/active-practice-charter.
15. Quirk, H. and S. Haake, *How can we get more people with long-term health conditions involved in parkrun? A qualitative study evaluating parkrun's PROVE project.* BMC Sports Sci Med Rehabil, 2019. **11**(1): p. 22.
16. Brannan, M., et al., *Moving healthcare professionals–a whole system approach to embed physical activity in clinical practice.* BMC Med Educ, 2019. **19**(1): p. 84.
17. Faculty of Sport and Exercise Medicine. *Moving Medicine*. 2018.
18. Avery, L., et al., *Systematic development of a theory-informed multifaceted behavioural intervention to increase physical activity of adults with type 2 diabetes in routine primary care: Movement as Medicine for Type 2 Diabetes.* Implement Sci, 2016. **11**(1).
19. Undefeatable, W.A. *We Are Undefeatable; About the campaign*. 2019 17 July 2020].
