# Supplementary material for: ‘Function First’: how to promote physical activity and physical function in people with long-term conditions managed in primary care? A study combining realist and co-design methods
Source: BMJ Open. 2021 Jul 27;11(7):e046751. doi: 10.1136/bmjopen-2020-046751 (PMC8317101; doi:10.1136/bmjopen-2020-046751)
Supplement: Author's manuscript [file bmjopen-2020-046751.draft_revisions.pdf]

BMJ Open is committed to open peer review. As part of this commitment we make the peer review history of every article we publish publicly available.

When an article is published we post the peer reviewers' comments and the authors' responses online. We also post the versions of the paper that were used during peer review. These are the versions that the peer review comments apply to.

The versions of the paper that follow are the versions that were submitted during the peer review process. They are not the versions of record or the final published versions. They should not be cited or distributed as the published version of this manuscript.

BMJ Open is an open access journal and the full, final, typeset and author-corrected version of record of the manuscript is available on our site with no access controls, subscription charges or pay-per-view fees (http://bmjopen.bmj.com).

If you have any questions on BMJ Open's open peer review process please email info.bmjopen@bmj.com

BMJ Open

'Function First': How to promote physical activity and physical function in people with long-term conditions managed in primary care? A study combining realist and co-design methods.

Journal:	*BMJ Open*
Manuscript ID	bmjopen-2020-046751
Article Type:	Original research
Date Submitted by the Author:	11-Nov-2020

Complete List of Authors:	Law, Rebecca-Jane; Bangor University, North Wales Centre for Primary Care Research<br>Langley, Joseph; Sheffield Hallam University, Lab4Living<br>Hall, Beth; Bangor University, Library and Archives Services<br>Burton, Christopher; Canterbury Christ Church University, School of Allied Health Professions<br>Hiscock, Julia; Bangor University, North Wales Centre for Primary Care Research<br>Williams, Lynne; Bangor University, School of Healthcare Sciences<br>Morrison, Val; School of Psychology, Bangor University<br>Lemmey, Andrew; Bangor University, School of Sports, Health and Exercise Science<br>Lovell-Smith, Candida; Patient and Public Involvement Research Partner<br>Gallanders, John; Patient and Public Involvement Research Partner<br>Cooney, Jennifer; Bangor University, School of Sport, Health and Exercise Sciences<br>Williams, Nefyn; University of Liverpool, Health Services Research

Keywords:	PRIMARY CARE, QUALITATIVE RESEARCH, REHABILITATION MEDICINE, SPORTS MEDICINE

SCHOLARONE™
Manuscripts

I, the Submitting Author has the right to grant and does grant on behalf of all authors of the Work (as defined in the below author licence), an exclusive licence and/or a non-exclusive licence for contributions from authors who are: i) UK Crown employees; ii) where BMJ has agreed a CC-BY licence shall apply, and/or iii) in accordance with the terms applicable for US Federal Government officers or employees acting as part of their official duties; on a worldwide, perpetual, irrevocable, royalty-free basis to BMJ Publishing Group Ltd ("BMJ") its licensees and where the relevant Journal is co-owned by BMJ to the co-owners of the Journal, to publish the Work in this journal and any other BMJ products and to exploit all rights, as set out in our licence.

The Submitting Author accepts and understands that any supply made under these terms is made by BMJ to the Submitting Author unless you are acting as an employee on behalf of your employer or a postgraduate student of an affiliated institution which is paying any applicable article publishing charge ("APC") for Open Access articles. Where the Submitting Author wishes to make the Work available on an Open Access basis (and intends to pay the relevant APC), the terms of reuse of such Open Access shall be governed by a Creative Commons licence – details of these licences and which Creative Commons licence will apply to this Work are set out in our licence referred to above.

Other than as permitted in any relevant BMJ Author's Self Archiving Policies, I confirm this Work has not been accepted for publication elsewhere, is not being considered for publication elsewhere and does not duplicate material already published. I confirm all authors consent to publication of this Work and authorise the granting of this licence.

For peer review only

Function First - Be Active, Stay Independent, BMJ Open submission

Title:

'Function First': How to promote physical activity and physical function in people with long-term conditions managed in primary care? A study combining realist and co-design methods.

Corresponding author details:

Name: Dr Rebecca-Jane Law

Address: North Wales Centre for Primary Care Research

Bangor University

Cambrian House 2

Wrexham Technology Park

Wrexham

LL13 7YP

Email: r.law@bangor.ac.uk

Telephone: 01248 383520

Fax: N/A

Author List

1) Law, Rebecca-Jane; School of Health Sciences, Bangor University, Wrexham, Wales, UK
   ORCID: 0000-0002-1435-5086
2) Langley, Joseph; Lab4Living, Sheffield Hallam University, Sheffield, England, UK
   ORCID: 0000-0002-9770-8720
3) Hall, Beth; Library and Archives Services, Bangor University, Bangor, Wales, UK
   ORCID: 0000-0003-4980-3720
4) Burton, Christopher; School of Allied and Public Health Professions, Canterbury Christ Church University, Kent, England, UK
   ORCID: 0000-0003-1159-1494
5) Hiscock, Julia; School of Health Sciences, Bangor University, Bangor, Wales, UK
   ORCID: 0000-0002-8963-2981
6) Williams, Lynne; School of Health Sciences, Bangor University, Bangor, Wales, UK
   ORCID: 0000-0003-2575-9710
7) Morrison, Val; School of Psychology, Bangor University, Bangor, Wales, UK
   ORCID: 0000 0002 4308 8976

For peer review only - http://bmjopen.bmj.com/site/about/guidelines.xhtml

8) Lemmey, Andrew Bruce; School of Sport, Health and Exercise Sciences, Bangor University, Bangor, Wales, UK

   ORCID: 0000-0003-1667-4539

9) Lovell-Smith, Candida; PPI research partner

10) Gallanders, John; PPI research partner

11) Cooney, Jennifer; School of Sport, Health and Exercise Sciences, Bangor University, Bangor, Wales, UK

    ORCID: 0000-0002-9828-8000

12) Williams, Nefyn Howard: Department of Health Services Research, University of Liverpool, England, UK

    ORCID: 0000-0002-8078-409X

Word count: 4111

For peer review only

Abstract (300/300 words words)

Objectives

To develop a programme theory explaining how interventions improve physical activity and function in people with long-term conditions managed in primary care. To produce an associated taxonomy of interventions, and co-design a prototype intervention.

Design

Realist synthesis combining evidence from a wide range of rich and relevant literature with stakeholder views. Resulting context, mechanism and outcome (CMO) statements informed co-design and knowledge mobilisation workshops with stakeholders to develop a primary care service innovation.

Results

Abridged CMO statements:

1) GP consultations tend not to prioritise physical activity and function. If the culture of the practice is supportive of physical activity, then encouraging physical activity will become part of usual routine.
2) Physical activity promotion is inconsistent and uncoordinated. If physical activity promotion is better resourced, then this will improve opportunities to change behaviour.
3) People with long-term conditions have varying physical activity levels, attitudes and opportunities. If physical activity promotion is adapted to individual needs and preferences, then people will be more likely to carry on.
4) Many general practice staff lack knowledge and confidence about promoting physical activity. If training makes staff more capable, then they will be better able to promote physical activity.
5) If a programme makes sense and is trustworthy, then patients and professionals will engage with it.

A taxonomy was produced, including thirteen categories of physical activity interventions for people with long-term conditions.

Resources were co-designed for developing: an environment and culture that encourages physical activity, knowledge about physical activity, and a new role for someone who can encourage people to use local opportunities to be more active.

For peer review only - http://bmjopen.bmj.com/site/about/guidelines.xhtml

Conclusions

This product needs further development and consideration alongside current schemes and contexts, including those relevant to the Covid-19 pandemic, and then testing in a future study. The integration of realist and co-design methods strengthened this study.

Keywords

Physical activity, physical function, long-term conditions, primary care, realist synthesis, co-design, co-production

Strengths and limitations of this study

1) Co-production with stakeholders was embedded in all stages of the project to enhance the attention to context that is characteristic of a realist approach.
2) Despite systematic, comprehensive and transparent literature searches to identify a wide range of evidence, many papers lacked detailed descriptions of organisational context, characteristics of individuals, and circumstances that led to the success of the intervention.
3) The study became more iterative, facilitating greater integration of the different data sources and enhancing the depth and breadth of the findings.
4) As with all findings based on qualitative methods, the current findings may not apply to other contexts. However, we co-designed a set of flexible resources that embodied the programme theories, but which could adapt to different contexts and augment existing initiatives.
5) These resources need further development and refinement before they can be used in primary care consultation

For peer review only

Introduction

In 2019 in the UK, more than 18 million adults over the age of 18 years had a long-term condition (i.e. 38% of the total adult population) [1]. Approximately 25% of people with one long-term condition report 'problems performing usual activities', rising to over 60% in those with three or more long-term conditions [2]. As older people accumulate more long-term conditions, they become increasingly frail [3-5]. This is one of the biggest challenges facing the health and social care systems [6].

There are known benefits of physical activity in the management of long-term conditions, including improved physical and psychosocial functioning [7-13]. However, the proportion of the adult population in England and Wales that are at least moderately active is low, [14, 15] and even lower in people with long-term conditions. There is an inverse association between habitual physical activity level and multi-morbidity [16, 17].

Primary care is well placed to empower individuals and communities to improve physical activity and function, because 90% of patients' interaction with the National Health Service (NHS) occurs in this setting [18]. However, primary care management of long-term conditions typically focusses on the diagnosis and management of disease, and not on increasing physical activity. A better way for primary care to promote physical activity and reduce functional decline is needed, and is likely to involve a complex intervention. In order to understand the active ingredients of such an intervention, a method that focusses on complexity is required. A realist approach provides a contextualised, explanatory understanding of what works, for whom, in what circumstances, in what respects and over what duration [19-21]. Integrating this with co-design gives new ideas tangible form and tests how these will work in the real world [22].

For peer review only

Objectives

The overall aim was to conduct a realist evidence synthesis, informing the development of a primary care intervention to promote physical activity and physical function for people with long-term conditions. Specific objectives were:

1) To produce a taxonomy of physical activity interventions that aim to reduce functional decline in people with long-term conditions managed in primary care.

For peer review only - http://bmjopen.bmj.com/site/about/guidelines.xhtml

2) To work with patients, health professionals and researchers to uncover the complexity associated with the range of physical activity interventions in primary care, and how these directly or indirectly affect the physical functioning of people with long-term conditions.

3) To identify the mechanisms through which interventions bring about functional improvements in people with long-term conditions, and the circumstances associated with how the interventions are organised and operate within different primary care contexts.

4) To understand the potential impacts of these interventions across primary care and other settings, such as secondary healthcare and social care, paying attention to the conditions that influence how they operate.

5) To co-produce an evidence-based, theory-driven explanatory account, in the form of refined programme theory to underpin and develop a new intervention through a co-design process with patients, health professionals and researchers.

For peer review only

Method

We performed a realist synthesis of literature, with input from key stakeholders, including people with long-term conditions, study management and advisory groups. Co-production was embedded throughout including two participatory theory-building workshops, three co-design workshops and a final knowledge mobilisation workshop, from which ideas for an intervention were developed. The process was iterative, with data sources informing each other as the synthesis progressed (Figure 1). The overall methods are detailed elsewhere [23, 24] and a visual summary is provided in Supplementary Figure 1.

Participants

A stakeholder analysis enabled identification and targeting of the most relevant groups for the different stages of the synthesis and co-design [25]. It included representation from people with long-term conditions, primary care health professionals, allied health professionals, third-sector organisations, council-funded initiatives, social care, policy-makers and commissioners (Supplementary Table 1).

Development of initial programme theory

For peer review only - http://bmjopen.bmj.com/site/about/guidelines.xhtml

Two theory-building stakeholder workshops and an early scoping search of published and grey literature developed initial ideas for programme theories. We used LEGO® SERIOUS PLAY® as a participatory method for the workshops to enable expression and creativity through building models and sharing (Supplementary Figure 2). A preliminary list of 'if…then' statements was created (Supplementary Table 2), which informed the first co-design workshop, the literature search strategy and inclusion/exclusion criteria.

Literature searching and data extraction

We started the review by examining relevant systematic reviews and developed eight 'theory areas': promoting physical literacy across the practice team; framing physical activity promotion around the link between physical activity and physical function; routinely assessing and promoting physical function and activity; reducing time pressure by offering consultation with a credible professional; linking people into existing local initiatives; using behaviour change techniques; tailoring advice and goals; and social support from others. Our literature search identified 73 articles, selected for inclusion because of relevance and theoretical richness. We supplemented this systematic search with forward and backwards citation tracking of key articles and purposive searches of guidelines, grey literature, social prescribing and physical literacy to identify 48 additional articles (Figure 2). Bespoke data extraction forms captured data informing the developing programme theories.

Testing and refining programme theories

The theory areas were explored in 10 'theory-refining' telephone interviews with stakeholders and as part of the second co-design workshop. We extracted data from papers identified as rich and relevant to the theory areas. Through this process and iterative discussion, we developed initial 'candidate' Context, Mechanism and Outcome (CMO) statements. These CMOs were continually refined, throughout the later workshops.

Co-design workshops

Three consecutive co-design workshops included a range of stakeholders (n = 23), including people living with long-term conditions, primary care professionals, a primary care cluster lead with a third sector perspective, a life coach, exercise referral scheme coordinator, researchers and members of the Function First research team. A team of design researchers facilitated the workshops. Ideas were

For peer review only - http://bmjopen.bmj.com/site/about/guidelines.xhtml

developed for an intervention to promote physical activity for people with long-term conditions managed in primary care. Recommendations for service innovation, and plans for making the intervention useable, were designed collaboratively and expanded during each workshop. There were key 'deliverables' from each workshop and, in between workshops, designers worked to develop ideas and provocations for the next workshop, termed 'design activities'.

Knowledge mobilisation workshop

This workshop involved people with long-term conditions, primary care professionals and researchers (n = 12) and explored how best to implement the prototype intervention in different contexts, ensuring that it was desirable, feasible and viable. The design researchers presented a physical example, which embodied the top four concepts generated in the co-design phase: 'directory of local assets', 'specialist role', 'training for health professionals' and 'community transport'. These ideas were refined through input from an external panel including representation from professional bodies for general practice, nursing and physiotherapy, and public health. Whilst detailed content was missing, demonstration of the intervention ideas illustrated how each physical element related to the refined CMO statements, creating an evidence-informed design solution (Figure 3).

Patient and public involvement

Five public research partners were proactively engaged throughout the project and contributed to monthly study management and quarterly external project advisory group meetings. They participated in decision-making, research activities (e.g. group analysis sessions), reviewing public-facing documents, authoring reports and providing feedback on findings as they emerged.

Results

A taxonomy of primary care physical activity interventions for people with long-term conditions was produced and included the following categories: brief interventions [26]; telephone interventions [27]; online/'eHealth' interventions [28]; exercise referral schemes [29]; community 'navigators' [30]; referral to exercise specialists (e.g. exercise physiologists) [31]; intervention delivery by existing primary care staff [32]; physical activity 'pathways' [33-38]; practice-wide initiatives [39]; community initiatives adopted by primary care [40]; a whole system approach to embed physical activity in clinical practice [41, 42]; multi-faceted interventions [43]; and campaigns [44] (Supplementary Table 3).

For peer review only - http://bmjopen.bmj.com/site/about/guidelines.xhtml

Five context (C), mechanism (M), and outcome (O) statements were developed. These explain how the contexts and mechanisms identified lead to outcomes relevant to improving physical activity and physical function in people with long-term conditions. Each explanatory account below highlights meaning and illustrates salient points with examples of evidence from the literature and stakeholder interviews.

1) Changing practice culture through alignment

Primary care settings are characterised by competing demands, and improving physical activity and physical function is often not prioritised in a busy practice (C). However, if the practice team culture can be aligned to promote and support the elements of physical literacy (M), then physical activity promotion will become more routine and embedded in usual care (O).

Lack of time and competing priorities limit discussion of physical activity in primary care [45-47].

"I think physical activity unfortunately does take a bit of a back step because it's probably not seen as so important as referring somebody who is expected cancer or sorting somebody's medications out." (General practitioner, individual interview)

Competing priorities include different models of care, with the primary care management of long-term conditions typically focussing on the diagnosis of disease according to the International Classification of Diseases [48]. The International Classification of Functioning, Disability and Health places more emphasis on functional limitations in a biopsychosocial context [49]. In this context, physical activity has the potential to promote a more pro-active, 'whole person' and preventive care approach [50, 51]. However the time and resource limitations in primary care act as barriers to the implementation of this approach [46, 52].

Physical literacy is defined as "the motivation, confidence, physical competence, knowledge and understanding to value and take responsibility for engagement in physical activities for life" [53]. Aligning practice culture with physical literacy could facilitate successful physical activity promotion. A physical literacy model for adults aged 65 years and older has been developed [54] and in the UK, the Active Practice Charter aims to enhance the culture of physical activity promotion across the primary care setting [39].

Interventions are more likely to be effective when integrated into routine practice [55, 56]. For example, the 'Let's Get Moving' pathway involved primary care embedding a physical activity promotion pathway into routine practice. However, the pathway was less successful when implemented more widely, required modifications and lacked the simplicity required to align with

For peer review only - http://bmjopen.bmj.com/site/about/guidelines.xhtml

existing programmes [33, 37, 38]. Care is also needed to reduce the burden of routine physical activity promotion.

"But, would I want any more forms to fill in or boxes to tick or guidance that says, "If you can touch your toes and tie up your shoelaces without getting breathless you score a one…it wouldn't help me at all" (General Practitioner, individual interview).

In order to encourage the promotion of physical activity 'as routine', protocols, pathways and procedures are insufficient; strategies are needed that align the practice team, settings and systems with the principles of physical literacy.

*2) **Providing resources***

Physical activity promotion in primary care is inconsistent and uncoordinated (C). If specific resources are allocated to physical activity promotion (in combination with a practice culture which is supportive) (M), then this will improve opportunities to change behaviour (O).

Despite a rise in initiatives and research [57], physical activity promotion in primary care remains inconsistent [58-63]. Exercise referral schemes have shown small effects on physical activity [29], but low attendance and completion rates [64, 65], and there are many barriers to referral [66]. To reduce burden on GPs, many interventions have allocated specific resources to physical activity promotion by identifying alternative professionals to deliver physical activity advice. Practice nurses [67-72], healthcare assistants [73], expert patients [74] physical activity 'coaches', 'counsellors' or 'facilitators' [75-78], exercise professionals [79], physiotherapists [80, 81], accredited exercise physiologists [82] and different combinations of allied health professionals [50, 83] have been trained to apply their existing skills and work with patients on physical activity specific goals. Furthermore, social prescribing initiatives include physical activity promotion [84-87].

In a randomised controlled trial of referral from Australian primary care to exercise physiologists, a 12-week face-to-face and telephone coaching intervention resulted in participants completing the equivalent of 10 minutes more walking per day, which persisted after nine months [31]. The Exercise as a Vital Sign programme involved a medical assistant ascertaining a patient's self-reported physical activity prior to the GP entering the room, triggering exercise related care processes [88].

Primary care resource to advise patients about insufficient physical activity during routine consultations and link them to a robust referral system of physical activity opportunities could facilitate improvements in physical activity promotion and behaviour.

3) Individual advice

For peer review only - http://bmjopen.bmj.com/site/about/guidelines.xhtml

People with long-term conditions have varying levels of physical function and physical activity, varying attitudes to physical activity and differing access to local resources that enable physical activity (C). If physical activity promotion is adapted to individual needs, priorities and preferences, and considers local resource availability (M), then this will facilitate a sustained improvement in physical activity (O).

People with long-term conditions are on a spectrum of physical functioning and physical activity levels. Some people are already active, socially integrated and able to organise their everyday lives independently, whereas others have limited independence and rely on others for care [59, 89-91]. People are at varying stages in the behaviour change process [92, 93], as explained by one participant in this study:

'There's no point in people starting to dictate to people if they're not on board with it' (Public contributor, long-term condition, individual interview).

NICE guidance highlights the importance of recognising times when people may be more open to change [94]. A variety of approaches are required to encourage people with long-term conditions to start and maintain a physically active lifestyle in a personally relevant way. The use of behaviour change techniques have been emphasised in guidance, recommending the development of goals that consider individual contexts and the impact of social support [95, 96]. One-to-one sessions can be helpful to enable initial tailoring and review, whereas group-based activities can offer another source of motivation [79]. Group consultations for people with long-term conditions have shown positive effects, indicating the potential for use when resources are limited [97, 98].

Incorporating individualised, relevant and tailored advice has the potential to maximise relevance and effectiveness. Physical activity advice needs to avoid being too demanding [99], whilst providing sufficient challenge [100]. Interventions have acknowledged the unpredictable nature of living with a long-term condition by incorporating the ability to make adjustments over time [70, 71, 73, 98, 101-104]. Tailoring should link physical activity with personally relevant, enjoyable activities that are a 'good return' for the time and effort invested [82, 105-107], for example, canine-based interventions and community football schemes [108, 109]. Alternative ways of providing advice include online [28, 110, 111] or telephone counselling [27, 112, 113] which may be preferable for some people.

4) Improving capability of practice workforce

Many primary care practice staff have a lack of knowledge and confidence to promote physical activity (C). If staff develop an improved sense of capability through education and training (M), then they will increase their engagement in physical activity promotion (O).

For peer review only - http://bmjopen.bmj.com/site/about/guidelines.xhtml

People with long-term conditions are familiar with primary care and typically have established trust and rapport with staff [90, 114], however staff lack knowledge due to limited training and resources. An online survey of self-selecting GPs in England, found that only 20% were familiar with the national physical activity guidelines, 26% were not familiar with any physical activity assessment tools and 55% reported that they had not undertaken any training to encourage physical activity [58]. Indeed, only very limited medical curriculum time is devoted to physical activity and health [115-118]. Professionals described particular difficulties delivering motivational components, such as improving self-efficacy, which were delivered less comprehensively as a result [32, 119, 120]. They lacked confidence, knowledge and understanding about roles and responsibilities for physical activity promotion [121, 122].

Interventions such as 'Movement as Medicine' [43], 'Moving Healthcare Professionals' [41] and 'Moving Medicine' [42] have addressed this need and aim to provide more training and education for primary care health professionals. In particular, 'Moving Medicine' [42] aims to incorporate conversations about physical activity during routine care, and offers online support relevant to patients of all ages with different long-term conditions. Improved education should increase the confidence of healthcare professionals in delivering physical activity advice [123].

5) Programme credibility

If a programme is credible (C), then trust and confidence in the programme will develop (M) and more patients and professionals engage with the programme (O).

Established programmes that take place in hospitals or leisure centres, and are delivered by qualified personnel (e.g. cardiac rehabilitation or exercise referral schemes), have a high degree of credibility due to their association with the health service, relevant regulatory bodies and inclusion as part of NICE guidance [95]. GP referrals are often chosen as a strategy because recommendation from a known and trusted professional is felt to increase uptake [31, 90, 124].

A mixed-methods review of physical activity for people with osteoarthritis, found that advice was viewed as valuable if it came from a knowledgeable healthcare professional who could explain why a person should do something, tailors the advice, clearly specifies what to do and explains the benefits [114]. Active health professionals are more likely to provide better, more credible, and motivating advice to their patients [125]. Credibility can also be achieved by including peer-led elements as this tends to increase self-efficacy amongst patients receiving advice, enhance empathy and improve the likelihood of realistic advice being given [74, 126, 127]. Understanding, tolerance, taking a genuine interest, encouragement and support were also important qualities [114].

For peer review only - http://bmjopen.bmj.com/site/about/guidelines.xhtml

"It needs to be someone who is really qualified, got a good track record. They do the assessments...part of the assessment is talking to people for a while, not just 5 minutes and that's it" (Public contributor, long-term condition).

Both professionals and patients need to feel that a programme is safe [128, 129] and effective in order to engage with it [130]. Professional acceptance and implementation is more likely if an intervention is accompanied by an evaluation that determines its effectiveness and benefit [57].

Intervention co-design

A prototype multi-component intervention was co-designed, embodying the five programme theories and providing resources to promote physical activity and physical function for people with long-term conditions (Figure 4 and Table 1).

The prototype consisted of:

- Resources designed to encourage a culture of physical literacy amongst staff and within the practice.
- Suggestions for changing the physical layout of the practice and promotional materials to create an environment that encourages physical activity.
- Materials to help develop the role of a credible professional who would facilitate behaviour change during bespoke consultations with people with long-term conditions.
- Identification of community resources, which can address barriers to the uptake of physical activity, such as community transport schemes.
- Plans to develop, or adapt, an electronic directory of local physical activity opportunities, clubs and groups.

Table 1: The components of a conceptual online 'Function First' resource

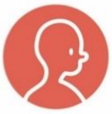	**Patients/General Public**
	Anyone participating in the Function First sessions could benefit from an online profile that tracks their progress, helps keep track of their follow-up consultation schedule and supports them with bespoke, personalised activity recommendations. The general public get access to the complete directory of local activities and transport.
	**GP & Surgery**

For peer review only

For peer review only - http://bmjopen.bmj.com/site/about/guidelines.xhtml

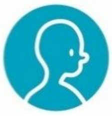	This profile exists as a way for the GP to access the activity record of any patient attending the Function First group sessions. Each member of staff may also have a personal profile as a member of the general public to benefit from the recommendations and access to the physical activity directory.
	**Credible Professional/Physical Activity Advisor (PAA)**
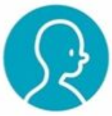	The Advisor could have the ability to edit the patient's profile or activity plan based on the recommendations made during a session. A part of these sessions could be a walk-through of how the online directory works. In addition to this, it would be desirable for the Advisor to begin to grow the network of activities and transport links by fostering communication between parties.
	**Community Transport**
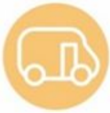	Transport services would be able to list their service in a separate transport section of the directory. Information about the operating area, capacity, number of vehicles, accessibility options and other information can be made available here, as well as direct contact info. An added benefit of this is that transport providers are often operated by volunteers who may also benefit from running this service.
	**Community Activities**
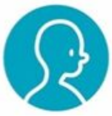	Activity providers would be able to list their service in a dedicated section of the directory. Information about the activity, intensity, cost, capacity, accessibility options and other information can be made available here, as well as direct contact information. An added benefit of this is that activities are often run by volunteers who may also benefit from running this service.

Discussion

Summary of main findings

'Function First' is the first realist evidence synthesis of physical activity promotion for people with long-term conditions managed in primary care. We developed five theoretical statements of what works, for whom and in what circumstances. From this programme theory, we co-designed flexible resources for use by a dedicated person working in primary care to promote physical activity.

Strengths and limitations

The realist approach offered a theory-driven explanation of the promotion of physical activity and function, paying particular attention to context. To enhance this, we embedded co-production with stakeholders at all stages thus incorporating the different perspectives of people with long-term conditions, primary care staff, and the systems in which they live and work.

For peer review only

The study was planned as a linear, sequential process, but became more iterative during the course of the study. This facilitated greater integration of the different data sources and enhanced the depth and breadth of the findings.

We carried out systematic, comprehensive and transparent literature searches to identify a wide range of evidence and used Covidence software [131] to enable team contribution to reviewing the large dataset of publications. We aimed to identify and present the most relevant and rich evidence, however many lacked detailed descriptions of organisational context, characteristics of individuals, and circumstances that led to the success of the intervention. We also found fewer reports of negative results, or difficulties in implementation.

A realist approach generates evidence-based recommendations that are context specific in terms of time, place and stakeholders. Similarly, co-design is criticised for being too specific, focusing on the needs of the participants in the process, resulting in personal rather than generalisable solutions. In addition, despite using a stakeholder analysis, our range of stakeholders included people with similar socioeconomic status, ethnicity, and attitudes. Therefore, the current findings may not apply to a different set of circumstances and the developed resources need further development and refinement before application. The changes to primary care associated with Covid-19 will need further consideration, including those related to remote consulting, practice re-organisation, use and implementation of evidence, patient behaviour and chronic disease management [132]. The need for physical opportunities to align with social distancing requirements and preferences, as well as mitigating against further health inequality resulting from the pandemic will also need to be considered [133, 134].

There are many initiatives promoting physical activity and from the outset, we desired to complement and not compete with these. Therefore, we involved representation from relevant bodies in our activities, and included a specific search for existing initiatives and campaigns. We co-designed a set of tangible resources that embodied all five programme theories, and are designed to be flexible and augment existing initiatives.

Comparison with existing literature

There are a limited number of existing realist evidence syntheses within the area of physical activity promotion for people with long-term conditions. A realist review exploring the referral of obese adults to weight management services identified similar contextual factors, including varying patient and practitioner characteristics and competing priorities. Practice level mechanisms included changes to

For peer review only - http://bmjopen.bmj.com/site/about/guidelines.xhtml

systems or culture, not assuming a standardised approach, and improving communication with weight management services [135]. Realist evidence suggests that health-related lifestyle advisors can remove barriers to healthy behaviour and create supportive social environments, but there is limited evidence of a positive impact on health knowledge, behaviour and outcomes [136]. A realist review of people living with Chronic Obstructive Pulmonary Disease and frailty found that mechanisms to maximise outcomes from exercise-based interventions included: trusting relationships; creating a shared understanding of needs; having the capacity to address multidimensional concerns; being able to individualise approaches to needs and priorities; and flexible approaches to intervention delivery. Finally, locating healthcare in leisure settings can create a physical environment that re-enforces physical activity culture and supports behaviour change, improve staff and patient experience, increase collaboration and coordination between health professionals, and increase awareness of facilities. Theories explaining the challenges of co-locating concerned the logistics of service delivery and clinical instability [137]. An example of a strategy designed to combine healthcare and physical activity and create a physical activity culture across a larger population is 'Move More' in Sheffield [138].

There are limited examples of applying realist methods to facilitate intervention development. In a study developing a rehabilitation intervention for elderly patients following hip fracture, three programme theories were developed: improving patient engagement by tailoring the intervention; reducing fear of falling and improving self-efficacy to exercise and perform activities of daily living; and coordination of rehabilitation delivery [139]. These informed the development of an enhanced rehabilitation intervention. 'Movement as Medicine' included stakeholder work to develop a prototype intervention [43] and the 'Choose to Move' programme in Canada used participatory methods to co-create new ways to enhance physical activity, mobility and social connectedness in older adults [140].

Implications for practice and research

If general medical practice in the UK is to address the low levels of physical activity and poor physical functioning of people with long-term conditions, then current practice culture needs to change. A new role of a credible professional could facilitate this, with appropriate resources and protected time, increased engagement with local providers of physical activity opportunities, and full utilisation of electronic directories developed for social prescribing. Improved undergraduate and continuing medical education about physical activity is also necessary to augment and sustain this change. The development of primary care networks, or clusters of practices, provides the opportunity for a

For peer review only - http://bmjopen.bmj.com/site/about/guidelines.xhtml

common, shared approach. This intervention will have cost implications, but may also have direct benefits to the NHS in terms of reduced consultations and demand for services.

A future research programme could further develop the intervention and assess its acceptability and effectiveness in the context of the Medical Research Council framework for evaluating complex interventions [141]. Remote co-design options, both digital and non-digital, that can be accessed electronically or posted to individuals may be needed to facilitate this development. The refined intervention, resources and new role need to fit in with existing schemes (e.g. National Exercise Referral Scheme and Moving Medicine) and complement public health campaigns (e.g. 'We Are Undefeatable) [29, 42, 44]. They also need to be flexible enough to adapt to different general medical practice contexts and changes associated with the Covid-19 pandemic.

Ethics

This study received approval from the Healthcare and Medical Sciences Academic Ethics Committee, Bangor University (Reference 2018-16308) and NHS Wales Research Ethics Committee 5 (References 256729 and 262726).

Funding statement

This project is funded by the National Institute for Health Research (NIHR) Health Services and Delivery Research programme (17/45/22). The views expressed are those of the author(s) and not necessarily those of the NIHR or the Department of Health and Social Care.

Competing interests

All authors had financial support from the National Institute for Health Research (NIHR) for the submitted work; NW is a GP partner at Plas Menai Health Centre, Llanfairfechan, Wales and is member of a NIHR Health Technology Assessment programme funding committee (commissioned research); there are no other relationships or activities that could appear to have influenced the submitted work.

Contribution of authors

For peer review only - http://bmjopen.bmj.com/site/about/guidelines.xhtml

Function First - Be Active, Stay Independent, BMJ Open submission

The contributions of the authors to different aspects of this work were as follows: conceiving the study and obtaining funding; R-JL, JL, BH, CB, JH, LW, VM, AL, CL-S, NW; gathering, analysis and interpretation of data: R-JL, JL, BH, CB, JH, LW, VM, AL, CL-S, JG, JC, NW; writing the report (wholly or in part): R-JL, JL, BH, CB, JH, LW, VM, AL, CL-S, JG, JC, NW; revising the report: R-JL, JL, BH, CB, JH, LW, VM, AL, CL-S, JG, JC, NW.

Acknowledgements

We are extremely grateful to all who contributed and supported this study as stakeholders and participants through engagement in workshops, interviews and meetings across the life of the project. We would also like to acknowledge the contribution and support of the Lab4Living research team at Sheffield Hallam University: Chris Redford who created the illustrations, storyboard and the graphics for the co-designed intervention resources, all of which were imperative to the progress and outcome of this project; Rebecca Partridge who was involved in developing and facilitating the overarching creative participatory processes, including the theory building stage and the co-design stage; Remi Bec who developed and facilitated the games and participatory activities for the co-design and knowledge mobilisation workshops and; Gemma Wheeler who also facilitated a co-design workshop. We would like to thank the following members of the study external project advisory group for their ongoing support, oversight and perspectives: Adrian Edwards (Chair), Robert Van Deursen, Julie Richardson, Asan Akpan, Jeanette Thom, Malcolm Ward, Louise Williams, Freya Davies, Andrea Hughes, Alan David Pryce. The authors would also like to thank Philip Bell who was a co-applicant and gave valuable insight from a public and patient perspective during the early stages of this project, and the research support teams within Health and Care Research Wales and the North West Coast Clinical Research Network who facilitated stakeholder involvement. Finally, from the North Wales Centre for Primary Care Research, School of Health Sciences at Bangor University, we would like to acknowledge and thank: Annie Hendry and Matthew Jones, for their help preparing the final report; Natasha Hulley, Nicola Nikolic and Richard Evans, for their ongoing administrative support throughout the project.

For peer review only - http://bmjopen.bmj.com/site/about/guidelines.xhtml

Figure legends

For peer review only

Figure 1. Schematic showing the iterative, integrated flow of information throughout the synthesis. Arrows indicate how each element informed another. The study management group and project advisory group meetings continuously informed the synthesis throughout the life of the project, and both groups involved input from public members.

Figure 2. Flowchart detailing the flow of information through the different phases of the review and the purposive searches

Figure 3: Physical variations, sample content detail and an image showing how the content was deconstructed and refined as part of the workshop.

Figure 4: Design image showing the components of Boxes 1 and 2 and their relation to the CMOs

For peer review only - http://bmjopen.bmj.com/site/about/guidelines.xhtml

References

1. Office for National Statistics. *People with long-term health conditions, UK: January to December 2019*. 2020 14 July 2020].
2. NHS Digital. *Health Survey for England, 2016*. National statistics 2017 05/04/2020].
3. Xue, Q.L., *The frailty syndrome: definition and natural history.* Clin Geriatr Med, 2011. **27**(1): p. 1-15.
4. Fried, L.P., et al., *Frailty in older adults: evidence for a phenotype.* J Gerontol A Biol Sci Med Sci, 2001. **56**(3).
5. Fried, L.P., et al., *Preclinical mobility disability predicts incident mobility disability in older women.* J Gerontol A Biol Sci Med Sci, 2000. **55**(1).
6. Department of Health and Social Care. *Long Term Conditions Compendium of Information: Third Edition*. 2012 05/04/2020].
7. Puts, M.T.E., et al., *Interventions to prevent or reduce the level of frailty in community-dwelling older adults: a scoping review of the literature and international policies.* Age Ageing, 2017. **46**(3): p. 383-392.
8. Geneen, L.J., et al., *Physical activity and exercise for chronic pain in adults: an overview of Cochrane Reviews.* Cochrane Database Syst Rev, 2017. **14**(1).
9. Larun, L., et al., *Exercise therapy for chronic fatigue syndrome.* Cochrane Database of Systematic Reviews, 2016.
10. Fransen, M., et al., *Exercise for osteoarthritis of the hip.* Cochrane Database Syst Rev, 2014. **22**(4).
11. Fransen, M., et al., *Exercise for osteoarthritis of the knee: a Cochrane systematic review.* Br J Sports Med, 2015. **49**(24): p. 1554-7.
12. Ashworth, N.L., et al., *Home versus center based physical activity programs in older adults.* Cochrane Database Syst Rev, 2005. **25**(1).
13. Heiwe, S. and S.H. Jacobson, *Exercise training for adults with chronic kidney disease.* Cochrane Database Syst Rev, 2011. **5**(10).
14. NHS Digital. *Statistics on Obesity, Physical Activity and Diet, England, 2019*. 2019 05/04/2020].
15. NHS Information Centre. *Health Survey for England 2008. Physical activity and fitness.* 2009 05/04/2020].
16. Cimarras-Otal, C., et al., *Association between physical activity, multimorbidity, self-rated health and functional limitation in the Spanish population.* BMC Public Health, 2014. **14**(1170): p. 1471-2458.
17. Autenrieth, C.S., et al., *Physical activity is inversely associated with multimorbidity in elderly men: results from the KORA-Age Augsburg Study.* Prev Med, 2013. **57**(1): p. 17-9.
18. Royal College of General Practitioners. *Health Select Committee Inquiry on Management of Long-Term Conditions*. 2013 October 2019].
19. Pawson, R., et al., *Realist review - a new method of systematic review designed for complex policy interventions.* J Health Serv Res Policy,, 2005. **10**(1): p. 21-34.
20. Pawson, R., *Evidence-based policy. A realist perspective.* London: Sage Publishing, 2006.
21. Pawson, R., *The science of evaluation. A realist manifesto.* London: Sage Publishing, 2013.
22. Cox, G., *Cox Review of Creativity in Business: building on the UK's strengths*, 2005: http://grips-public.mediactive.fr/knowledge_base/view/349/cox-review-of-creativity-in-business-building-on-the-uk-s-strengths/.
23. Law, R.J., et al., *'Function First—Be Active, Stay Independent'—promoting physical activity and physical function in people with long-term conditions by primary care: a protocol for a realist synthesis with embedded co-production and co-design* BMJ Open, 2020. **10**(2).

For peer review only

24. Law, R.J., et al., *Promoting physical activity and physical function in people with long-term conditions in primary care: Realist synthesis with co-design (Function First)* Health Services and Delivery Research, [in press].
25. Spitters, H.P., et al., *Unravelling networks in local public health policymaking in three European countries - a systems analysis.* Health Res Policy Syst, 2017. **15**(1): p. 5.
26. Lamming, L., et al., *What do we know about brief interventions for physical activity that could be delivered in primary care consultations? A systematic review of reviews.* Prev Med, 2017. **99**: p. 152-163.
27. Goode, A.D., M.M. Reeves, and E.G. Eakin, *Telephone-delivered interventions for physical activity and dietary behavior change: an updated systematic review.* Am J Prev Med, 2012. **42**(1): p. 81-88.
28. Muellmann, S., et al., *Effectiveness of eHealth interventions for the promotion of physical activity in older adults: A systematic review.* Prev Med, 2018. **108**: p. 93-110.
29. Campbell, F., et al., *A systematic review and economic evaluation of exercise referral schemes in primary care: a short report.* Health Technol Assess, 2015. **19**(60).
30. Leenaars, K.E., et al., *Exploring the impact of the care sport connector in the Netherlands.* BMC public health, 2017. **17**(1): p. 813.
31. Ewald, B., et al., *Physical activity coaching by Australian Exercise Physiologists is cost effective for patients referred from general practice.* Aust N Z J Public Health, 2018. **42**(1): p. 12-15.
32. Williams, S.L., et al., *Translating a walking intervention for health professional delivery within primary care: A mixed-methods treatment fidelity assessment.* Br J Health Psychol, 2019. **25**(1): p. 17-38.
33. Bull, F., K. Milton, and C. Boehler, *Evaluation of the Physical Activity Care Pathway London Feasibility Pilot–Final Technical Report*, 2008: https://assets.publishing.service.gov.uk/government/uploads/system/uploads/attachment_data/file/192041/Evaluation_of_the_Physical_Activity_Care_Pathway_London_Feasibility_Pilot_-_Report.pdf.
34. Bull, F.C. and K.E. Milton, *A process evaluation of a" physical activity pathway" in the primary care setting.* BMC Public Health, 2010. **10**(1): p. 463.
35. Boehler, C., et al., *The cost of changing physical activity behaviour: evidence from a "physical activity pathway" in the primary care setting.* BMC Public Health, 2011. **11**(1): p. 370-370.
36. Department of Health. *Resources for commissioning Let's Get Moving interventions* 2012 05/04/2020].
37. Loughren, E.A., C. Baker, and D. Crone, *'Let's Get Moving'Physical Activity Care Pathway (Gloucestershire) Post-Programme Evaluation Report.*, 2014: http://eprints.glos.ac.uk/2378/.
38. Barrett, E.M., J. Hussey, and C.D. Darker, *Feasibility of a physical activity pathway for Irish primary care physiotherapy services.* Physiotherapy, 2017. **103**(1): p. 106-112.
39. Royal College of General Practitioners. *RCGP Active Practice Charter*. 2019 [cited 2019; Available from: https://r1.dotdigital-pages.com/p/49LX-5IR/active-practice-charter.
40. Quirk, H. and S. Haake, *How can we get more people with long-term health conditions involved in parkrun? A qualitative study evaluating parkrun's PROVE project.* BMC Sports Sci Med Rehabil, 2019. **11**(1): p. 22.
41. Brannan, M., et al., *Moving healthcare professionals–a whole system approach to embed physical activity in clinical practice.* BMC Med Educ, 2019. **19**(1): p. 84.
42. Moving Medicine. *Moving Medicine*. 2018. Available from: https://movingmedicine.ac.uk/
43. Avery, L., et al., *Systematic development of a theory-informed multifaceted behavioural intervention to increase physical activity of adults with type 2 diabetes in routine primary care: Movement as Medicine for Type 2 Diabetes.* Implement Sci, 2016. **11**(1).
44. Undefeatable, W.A. *We Are Undefeatable; About the campaign*. 2019 17 July 2020].

For peer review only

45. Wheeler, P.C., et al., *Primary care knowledge and beliefs about physical activity and health: a survey of primary healthcare team members.* BJGP open, 2017. **1**(2).
46. Bull, F.C.L., et al., *Beliefs and behaviour of general practitioners regarding promotion of physical activity.* Australian Journal of Public Health, 1995. **19**(3): p. 300-304.
47. Melillo, K.D., et al., *Perceptions of nurse practitioners regarding their role in physical activity and exercise prescription for older adults.* Clinical excellence for nurse practitioners : the international journal of NPACE, 2000. **4**(2): p. 108-116.
48. World Health Organization, *ICD-10: international statistical classification of diseases and related health problems: tenth revision.* 2004.
49. World Health Organisation, *International classification of functioning, disability and health.* 2001.
50. Richardson, J., et al., *Monitoring physical functioning as the sixth vital sign: Evaluating patient and practice engagement in chronic illness care in a primary care setting - A quasi-experimental design.* BMC Family Practice, 2012. **13**.
51. Bierman, A.S., *Functional status, the sixth vital sign.* Journal of General Internal Medicine, 2001(16): p. 785-786.
52. Arden, K., F. Fatoye, and G. Yeowell, *Evaluation of a rolling rehabilitation programme for patients with non-specific low back pain in primary care: an observational cohort study.* Journal of Evaluation in Clinical Practice, 2017. **23**(2): p. 272-278.
53. International Physical Literacy Association. *Physical literacy can be described as the motivation, confidence, physical competence, knowledge and understanding to value and take responsibility for engagement in physical activities for life*. 2017; Available from: https://www.physical-literacy.org.uk/.
54. Jones, G.R., et al., *Development of a physical literacy model for older adults–a consensus process by the collaborative working group on physical literacy for older Canadians.* 2018.
55. Coulter, A., et al., *Personalised care planning for adults with chronic or long-term health conditions.* Cochrane Database Syst Rev, 2015. **3**(3).
56. Smith, S.M., et al., *Interventions for improving outcomes in patients with multi-morbidity in primary care and community settings.* Cochrane Database Syst Rev, 2012. **18**(4).
57. Lion, A., et al., *Physical activity promotion in primary care: a Utopian quest?* Health promotion international, 2019. **34**(4): p. 877-886.
58. Chatterjee, R., et al., *GPs' knowledge, use, and confidence in national physical activity and health guidelines and tools: a questionnaire-based survey of general practice in England.* Br J Gen Pract, 2017. **67**(663): p. e668-e675.
59. Hinrichs, T., et al., *General practitioner advice on physical activity: analyses in a cohort of older primary health care patients (getABI).* BMC family practice, 2011. **12**(1): p. 26.
60. Short, C.E., et al., *Physical activity recommendations from general practitioners in Australia. Results from a national survey.* Australian and New Zealand Journal of Public Health, 2016. **40**(1): p. 83-90.
61. Jorgensen, T.K., M. Nordentoft, and J. Krogh, *How do general practitioners in Denmark promote physical activity?* Scandinavian Journal of Primary Health Care, 2012. **30**(3): p. 141-146.
62. Omura, J.D., et al., *Primary care providers' physical activity counseling and referral practices and barriers for cardiovascular disease prevention.* Preventive Medicine, 2018. **108**: p. 115-122.
63. Lindeman, C., et al., *The extent to which family physicians record their patients' exercise in medical records: a scoping review.* BMJ open, 2020. **10**(2).
64. Murphy, S., et al., *An exploratory cluster randomised trial of a university halls of residence based social norms intervention in Wales, UK.* BMC Public Health, 2012. **12**(186): p. 1471-2458.

For peer review only

65. Moore, G.F., et al., *Mixed-method process evaluation of the welsh national exercise referral scheme.* Health Education 2013.
66. Din, N.U., et al., *Health professionals' perspectives on exercise referral from a process evaluation of the National Exercise Referral Scheme in Wales.* Health Education J 2015. **74**(6): p. 743-757.
67. Wilcox, S., et al., *Adoption and Implementation of Physical Activity and Dietary Counseling by Community Health Center Providers and Nurses*, 2004.
68. Croteau, K., G. Schofield, and G. McLean, *Physical activity advice in the primary care setting: results of a population study in New Zealand.* Australian and New Zealand journal of public health, 2006. **30**(3): p. 262-267.
69. Verwey, R.D.S.e., et al. *Upgrading physical activity counselling in primary care in the Netherlands*. Oxford University Press.
70. Verwey, R., et al., *A pilot study of a tool to stimulate physical activity in patients with COPD or type 2 diabetes in primary care.* Journal of Telemedicine and Telecare, 2014. **20**(1): p. 29-34.
71. Verwey, R., et al., *A monitoring and feedback tool embedded in a counselling protocol to increase physical activity of patients with COPD or type 2 diabetes in primary care: Study protocol of a three-arm cluster randomised controlled trial.* BMC Family Practice, 2014. **15**(1).
72. Verwey, R., et al., *Process evaluation of physical activity counselling with and without the use of mobile technology: A mixed methods study.* International Journal of Nursing Studies, 2016. **53**: p. 3-16.
73. Healey, E.L., et al., *The feasibility and acceptability of a physical activity intervention for older people with chronic musculoskeletal pain: The iPOPP pilot trial protocol.* Musculoskeletal Care, 2018. **16**(1): p. 118-132.
74. Van der Wulp, I., et al., *Effectiveness of peer-led self-management coaching for patients recently diagnosed with Type 2 diabetes mellitus in primary care: A randomized controlled trial.* Diabetic Medicine, 2012. **29**(10).
75. Morgan, G.S., et al., *Physical ACtivity facilitation for Elders (PACE): Study protocol for a randomised controlled trial.* Trials, 2015. **16**(1).
76. Shaw, R., et al., *Pre-exercise screening and health coaching in CHD secondary prevention: A qualitative study of the patient experience.* Health Education Research, 2012. **27**(3): p. 424-436.
77. Vanroy, J., et al., *Short- and long-term effects of a need-supportive physical activity intervention among patients with type 2 diabetes mellitus: A randomized controlled pilot trial.* PLoS ONE, 2017. **12**(4).
78. Rena Parish, T. and B.B.S. Tracie Rena Parish, *Examination of the constructs of the Transtheoretical model in patients with heart failure: a focus on physical activity readiness*, 2006.
79. Fife-Schaw, C., et al., *Comparing exercise interventions to increase persistence with physical exercise and sporting activity among people with hypertension or high normal blood pressure: Study protocol for a randomised controlled trial.* Trials, 2014. **15**(1).
80. McDonough, S.M., et al., *Pedometer-driven walking for chronic low back pain: A feasibility randomized controlled trial.* Clinical Journal of Pain, 2013. **29**(11): p. 972-981.
81. Comer, C., et al., *A Home Exercise Programme Is No More Beneficial than Advice and Education for People with Neurogenic Claudication: Results from a Randomised Controlled Trial.* PLoS ONE, 2013. **8**(9).
82. James, E.L., et al., *Referral for Expert Physical Activity Counseling: A Pragmatic RCT.* American Journal of Preventive Medicine 2017. **53**(4): p. 490–499.
83. Andryukhin, A., et al., *The impact of a nurse-led care programme on events and physical and psychosocial parameters in patients with heart failure with preserved ejection fraction: A*

For peer review only

randomized clinical trial in primary care in Russia. European Journal of General Practice, 2010. **16**(4): p. 205-214.

84. Smith, T.O., et al., *Social prescribing programmes to prevent or delay frailty in community-dwelling older adults.* Geriatrics, 2019. **4**(4): p. 65.
85. Bickerdike, L., et al., *Social prescribing: less rhetoric and more reality. A systematic review of the evidence.* BMJ open, 2017. **7**(4): p. e013384.
86. Husk, K., et al., *What approaches to social prescribing work, for whom, and in what circumstances? A realist review.* Health & social care in the community., 2019.
87. Pescheny, J.V., Y. Pappas, and G. Randhawa, *Facilitators and barriers of implementing and delivering social prescribing services: a systematic review.* BMC health services research, 2018. **18**(1): p. 86.
88. Grant, R.W., et al., *Exercise as a vital sign: A quasi-experimental analysis of a health system intervention to collect patient-reported exercise levels.* Journal of General Internal Medicine, 2014. **29**(2): p. 341-348.
89. Department of Health, P.A., Health Improvement and Protection, *Start Active, Stay Active: A report on physical activity from the four home countries' Chief Medical Officers (Now updated)*, 2011: https://assets.publishing.service.gov.uk/government/uploads/system/uploads/attachment_data/file/830943/withdrawn_dh_128210.pdf.
90. Hinrichs, T., et al., *Effects of an exercise programme for chronically ill and mobility-restricted elderly with structured support by the general practitioner's practice (HOMEfit) - study protocol of a randomised controlled trial.* Trials, 2011. **12**.
91. UK CMOs, *Physical activity guidelines: UK chief medical officers' report*, 2019: https://www.gov.uk/government/publications/physical-activity-guidelines-uk-chief-medical-officers-report.
92. Prochaska, J.O., C.C. DiClemente, and J.C. Norcross, *In search of how people change: Applications to addictive behaviors.* American Psychologist, 1992. **47**(9): p. pp. 1102–1114.
93. Marcus, B.H. and L.R. Simkin, *The transtheoretical model: applications to exercise behavior.* Medicine & Science in Sports & Exercise., 1994. **26**(11): p. pp. 1400–1404.
94. National Institute for Clinical Excellence, *Behaviour change: individual approaches*, 2014.
95. National Institute for Clinical Excellence, *Physical activity: exercise referral schemes*, 2014.
96. National Institute for Clinical Excellence, *Physical activity: brief advice for adults in primary care.* London: Nice public health guidance 2013: p. 44.
97. NHS Leading Change Adding Value Team. *Introducing group consultations for adults with Type 2 diabetes*. 2019 2020].
98. Åsenlöf, P., E. Denison, and P. Lindberg, *Individually tailored treatment targeting activity, motor behavior, and cognition reduces pain-related disability: A randomized controlled trial in patients with musculoskeletal pain.* Journal of Pain, 2005. **6**(9): p. 588-603.
99. Bjerk, M., et al., *A falls prevention programme to improve quality of life, physical function and falls efficacy in older people receiving home help services: Study protocol for a randomised controlled trial.* BMC Health Services Research, 2017. **17**(1).
100. Bearne, L.M., et al., *Feasibility of an exercise-based rehabilitation programme for chronic hip pain.* Musculoskeletal Care, 2011. **9**(3): p. 160-168.
101. Holden, M.A., et al., *Role of exercise for knee pain: What do older adults in the community think?* Arthritis Care and Research, 2012. **64**(10): p. 1554-1564.
102. Leijon, M.E., et al., *Physical activity referrals in Swedish primary health care - Prescriber and patient characteristics, reasons for prescriptions, and prescribed activities.* BMC Health Services Research, 2008. **8**.
103. Lohmann, H., V. Siersma, and N.F. Olivarius, *Fitness consultations in routine care of patients with type 2 diabetes in general practice: An 18-month non-randomised intervention study.* BMC Family Practice, 2010. **11**.

104. Prochaska, J.J., et al., *PACE Interactive Communication Technology for Behavior Change in Clinical Settings*, 2000. p. 127-131.
105. Hurley, M., et al., *Exercise interventions and patient beliefs for people with hip, knee or hip and knee osteoarthritis: A mixed methods review*, 2018, John Wiley and Sons Ltd.
106. Danielsson, L., B. Kihlbom, and S. Rosberg, *"Crawling Out of the Cocoon": Patients' Experiences of a Physical Therapy Exercise Intervention in the Treatment of Major Depression*, 2016.
107. Åsenlöf, P., E. Denison, and P. Lindberg, *Long-term follow-up of tailored behavioural treatment and exercise based physical therapy in persistent musculoskeletal pain: A randomized controlled trial in primary care.* European Journal of Pain, 2009. **13**(10): p. 1080-1088.
108. Rhodes, R.E., et al., *Increasing physical activity by four legs rather than two: systematic review of dog-facilitated physical activity interventions.* British Journal of Sports Medicine, 2020.
109. Bjerre, E.D., et al., *Community-based football in men with prostate cancer: 1-year follow-up on a pragmatic, multicentre randomised controlled trial.* PLoS medicine, 2019. **16**(10).
110. Chaplin, E., et al., *The evaluation of an interactive web-based Pulmonary Rehabilitation programme: protocol for the WEB SPACE for COPD feasibility study.* BMJ open, 2015. **5**(8): p. e008055.
111. Devi, R., J. Powell, and S. Singh, *A web-based program improves physical activity outcomes in a primary care angina population: Randomized controlled trial.* Journal of Medical Internet Research, 2014. **16**(9).
112. Weinstock, R.S., et al., *Lessened decline in physical activity and impairment of older adults with diabetes with telemedicine and pedometer use: results from the IDEATel study.* Age and Ageing, 2011. **40**(1): p. 98-105.
113. Allen, K.D., et al., *Patient and provider interventions for managing osteoarthritis in primary care: protocols for two randomized controlled trials*, 2012.
114. Forsyth, A., F.P. Deane, and P. Williams, *Dietitians and exercise physiologists in primary care: Lifestyle interventions for patients with depression and/or anxiety.* Journal of Allied Health, 2009.
115. Ward, M., *A Survey of Physical Activity in MedicalCurricula: A report of the HEPA in Health Care Settings*, 2015: HEPA Europe Working Group.
116. Dacey, M.L., et al., *Physical activity counseling in medical school education: a systematic review.* Medical education online, 2014. **19**(1): p. 24325.
117. Dunlop, M. and A.D. Murray, *Major limitations in knowledge of physical activity guidelines among UK medical students revealed: implications for the undergraduate medical curriculum.* Br J Sports Med, 2013. **47**(11): p. 718-720.
118. Weiler, R., et al., *Physical activity education in the undergraduate curricula of all UK medical schools. Are tomorrow's doctors equipped to follow clinical guidelines?* British journal of sports medicine, 2012. **46**(14): p. 1024-1026.
119. Jansink, R., et al., *Primary care nurses struggle with lifestyle counseling in diabetes care: a qualitative analysis.* BMC family practice, 2010. **11**(1): p. 41.
120. Sturgiss, E., et al., *Increasing general practitioners' confidence and self-efficacy in managing obesity: a mixed methods study* BMJ open 2017. **7**(1): p. e014314.
121. Rushforth, B., et al., *Barriers to effective management of type 2 diabetes in primary care: qualitative systematic review.* Br J Gen Pract, 2016. **66**(643): p. e114-e127.
122. Leemrijse, C.J., et al., *Collaboration of general practitioners and exercise providers in promotion of physical activity a written survey among general practitioners.* BMC Family Practice, 2015. **16**(1): p. 1-9.
123. Savill, B., A. Murray, and R. Weiler, *Is general practice engaged with physical activity promotion?* Br J Gen Pract, 2015. **65**(638): p. 484-485.

For peer review only

124. Stone, R.C. and J. Baker, *Painful choices: a qualitative exploration of facilitators and barriers to active lifestyles among adults with osteoarthritis.* Journal of Applied Gerontology, 2015. **36**(9): p. 1091-1116.
125. Lobelo, F., J. Duperly, and E. Frank, *Physical activity habits of doctors and medical students influence their counselling practices.* Br J Sports Med online, 2009. **43**(2): p. 89-92.
126. Gamboa Moreno, E., et al., *Impact of a self-care education programme on patients with type 2 diabetes in primary care in the Basque Country.* BMC Public Health, 2013. **13**(1).
127. Gamboa Moreno, E., et al., *A Pilot Study to Assess the Feasibility of the Spanish Diabetes Self-Management Program in the Basque Country.* Journal of diabetes research, 2016. **2016**: p. 9145673-9145673.
128. Walsh, J.M.E., et al., *Exercise Counseling by Primary Care Physicians in the Era of Managed Care*, 1999.
129. Hinrichs, T., et al., *Home-Based Exercise Supported by General Practitioner Practices: Ineffective in a Sample of Chronically Ill, Mobility-Limited Older Adults (the HOMEfit Randomized Controlled Trial).* Journal of the American Geriatrics Society, 2016. **64**(11): p. 2270-2279.
130. Marchal, B., et al., *Is realist evaluation keeping its promise? A review of published empirical studies in the field of health systems research* Evaluation, 2012. **18**(2): p. 192-212.
131. Covidence. *Better systematic review management: Covidence systematic review software*. 2020 05/04/2020].
132. Khan, N., et al., *A brave new world: the new normal for general practice after the COVID-19 pandemic.* BJGP open, 2020. **4**(3): p. bjgpopen20X101103.
133. Bambra, C., et al., *The COVID-19 pandemic and health inequalities.* Journal of Epidemiology and Community Health, 2020: p. jech-2020-214401.
134. Hall G, L.D., Phillips SA, Lavie CJ, Arena R., *A tale of two pandemics: How will COVID-19 and global trends in physical inactivity and sedentary behavior affect one another?* Prog Cardiovasc Dis., 2020. **8**.
135. Blane, D.N., S. Macdonald, and C.A. O'Donnell, *What works and why in the identification and referral of adults with comorbid obesity in primary care: A realist review.* Obes Rev, 2019. **21**(4): p. e12979.
136. Carr, S., et al., *An evidence synthesis of qualitative and quantitative research on component intervention techniques, effectiveness, cost-effectiveness, equity and acceptability of different versions of health-related lifestyle advisor role in improving health.* Health Technol Assess 2011. **15**(9): p. 1-284.
137. Grinvalds, N., et al. *What works to facilitate the promotion of physical activity in co-located healthcare and leisure settings: a realist review.* SportRχiv, 2019. DOI: https://doi.org/10.31236/osf.io/h9r8w.
138. Move More. *Move More Challenge: We are World Record breakers!* 2019 accessed 13/03/2020].
139. Roberts, J.L., et al., *Development of an evidence-based complex intervention for community rehabilitation of patients with hip fracture using realist review, survey and focus groups.* BMJ Open, 2017. **7**(10): p. e014362.
140. McKay, H., et al., *Implementation of a co-designed physical activity program for older adults: positive impact when delivered at scale.* BMC Public Health, 2018. **18**: p. 1289.
141. Craig, P., et al., *Developing and evaluating complex interventions: the new Medical Research Council guidance.* BMJ, 2008. **337**: p. a1655.

For peer review only

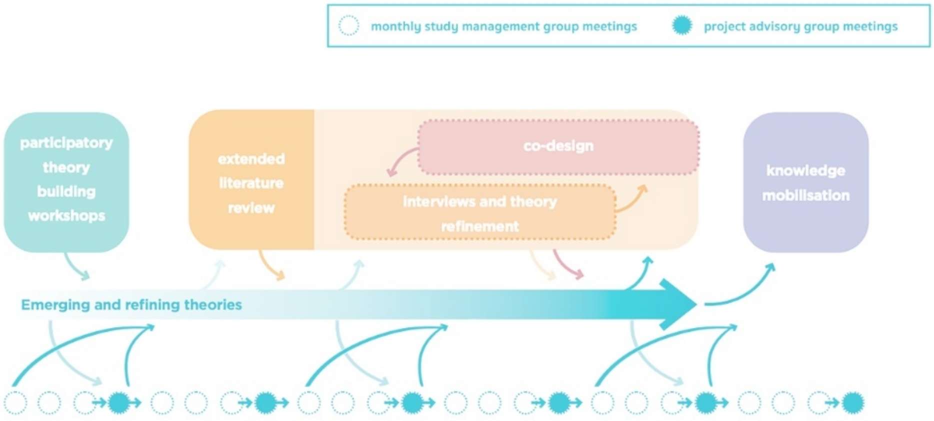

Figure 1. Schematic showing the iterative, integrated flow of information through the following five phases over an 18 month period: 1) participatory theory-building workshops; 2) extended literature review; 3) co-design; 4) interviews and theory refinement; 5) knowledge mobilisation. Arrows indicate how each element informed another. The study management group and project advisory group meetings continuously informed the synthesis throughout the life of the project, and both groups involved input from public members.

279x129mm (96 x 96 DPI)

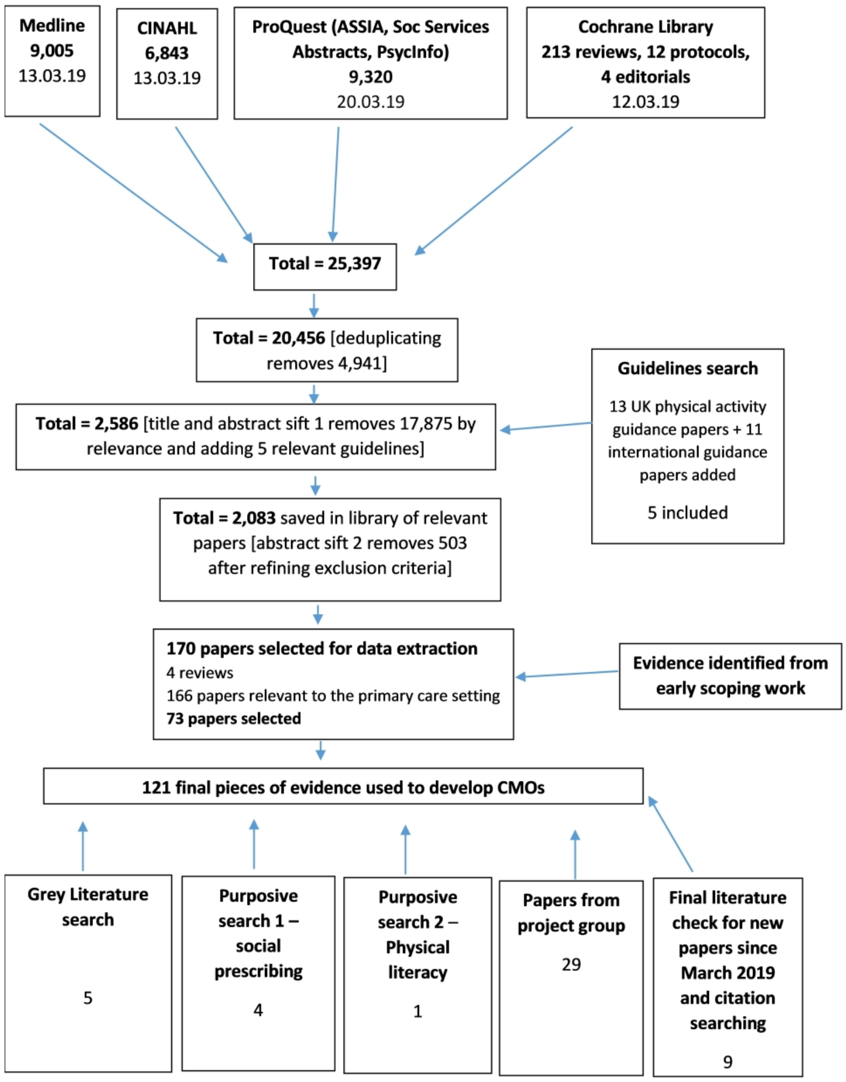

Figure 2: Flowchart detailing the flow of information through the different phases of the review and the purposive searches

1060x1514mm (96 x 96 DPI)

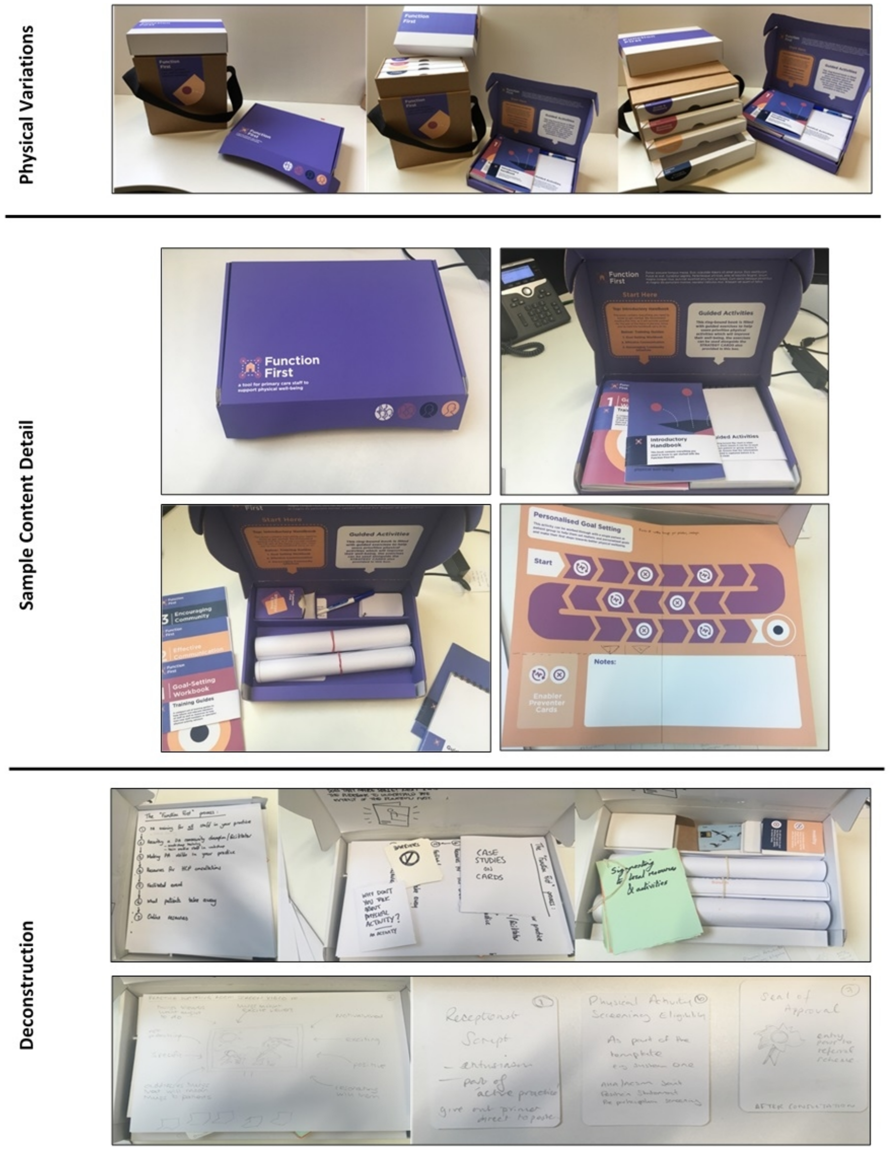

Figure 3: Physical variations, sample content detail and an image showing how the content was deconstructed and refined as part of the workshop.

260x298mm (96 x 96 DPI)

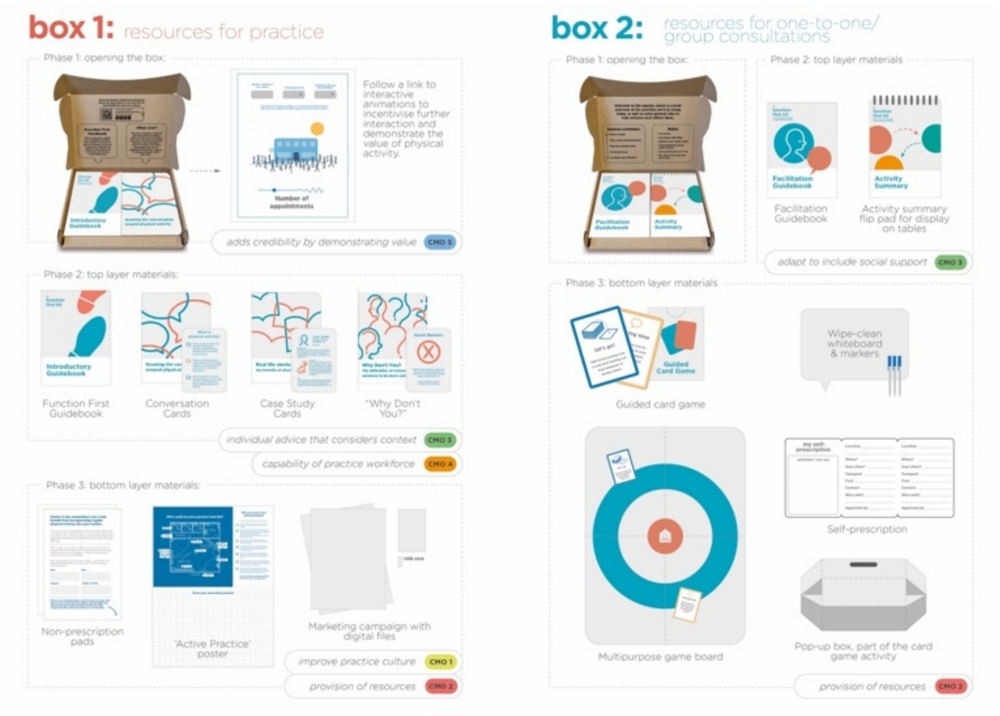

Figure 4: Design image showing the components of Boxes 1 and 2 and their relation to the CMOs

202x144mm (96 x 96 DPI)

Supplementary figure 1: Visual storyboard project summary

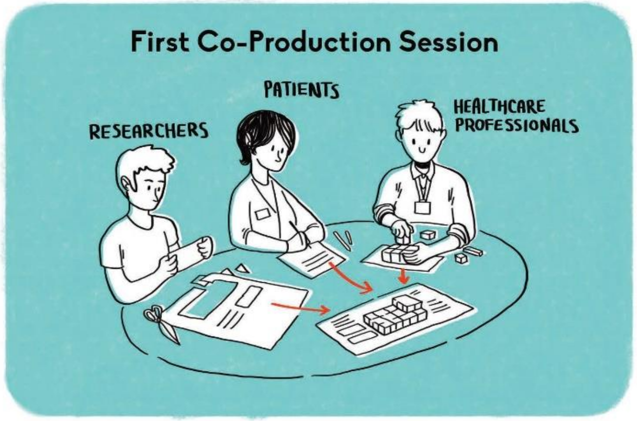

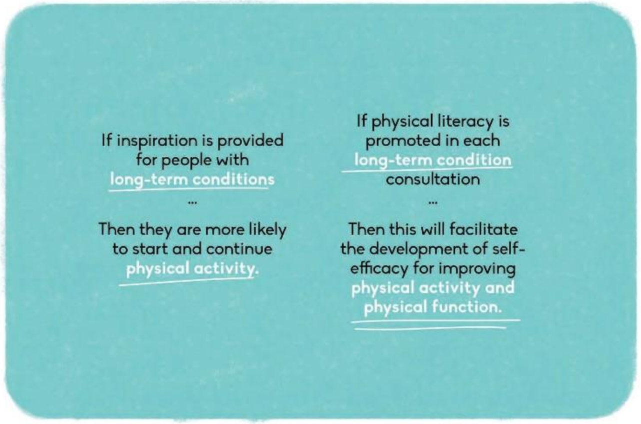

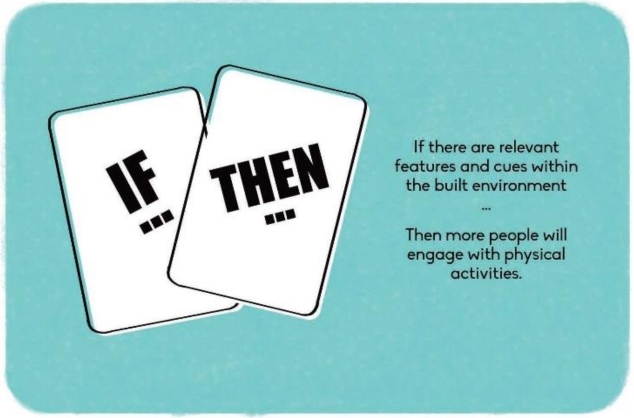

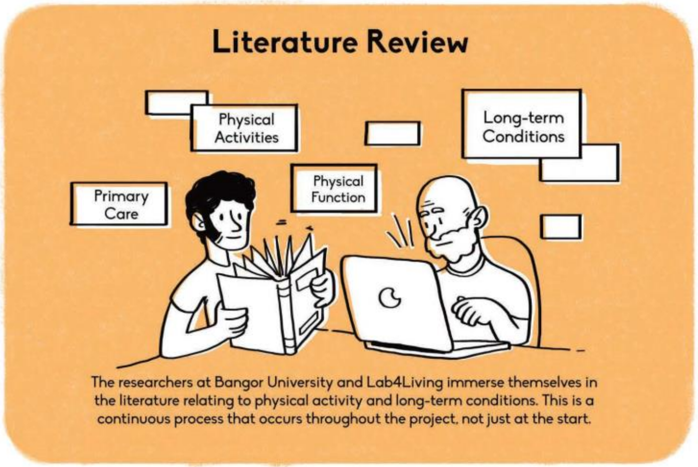
Literature Review
Physical Activities
Long-term Conditions
Primary Care
Physical Function
The researchers at Bangor University and Lab4Living immerse themselves in the literature relating to physical activity and long-term conditions. This is a continuous process that occurs throughout the project, not just at the start.

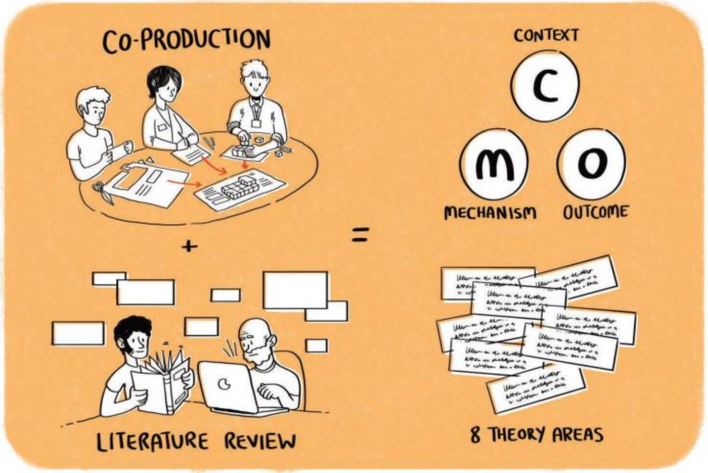
CO-PRODUCTION
+
=
CONTEXT
C
M
O
MECHANISM
OUTCOME
LITERATURE REVIEW
8 THEORY AREAS

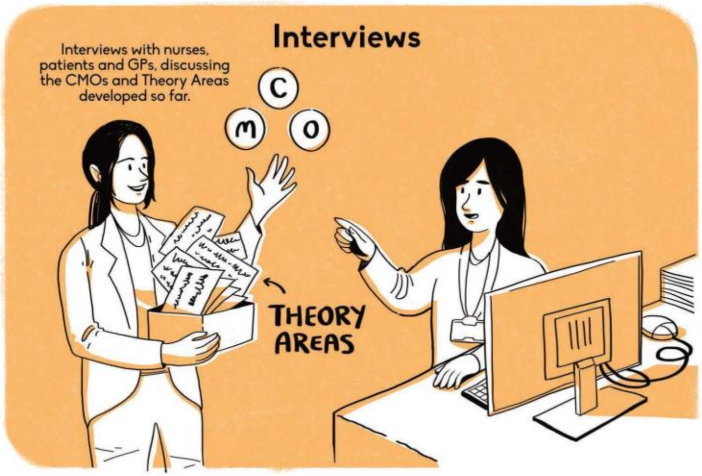
Interviews
Interviews with nurses, patients and GPs, discussing the CMOs and Theory Areas developed so far.
C
M
O
THEORY AREAS

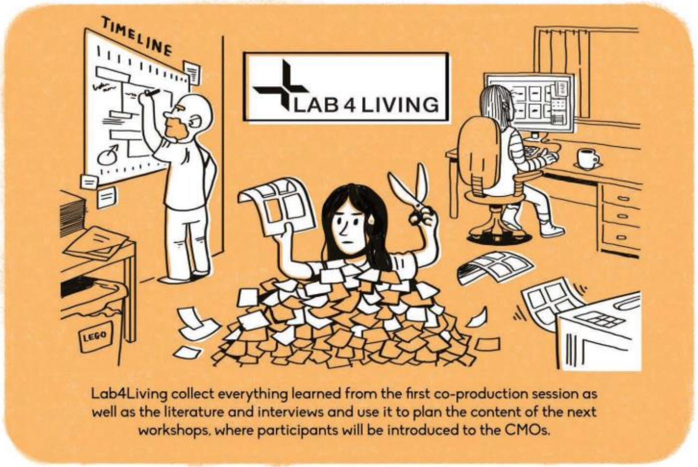
TIMELINE
LAB 4 LIVING
LEGO
Lab4Living collect everything learned from the first co-production session as well as the literature and interviews and use it to plan the content of the next workshops, where participants will be introduced to the CMOs.

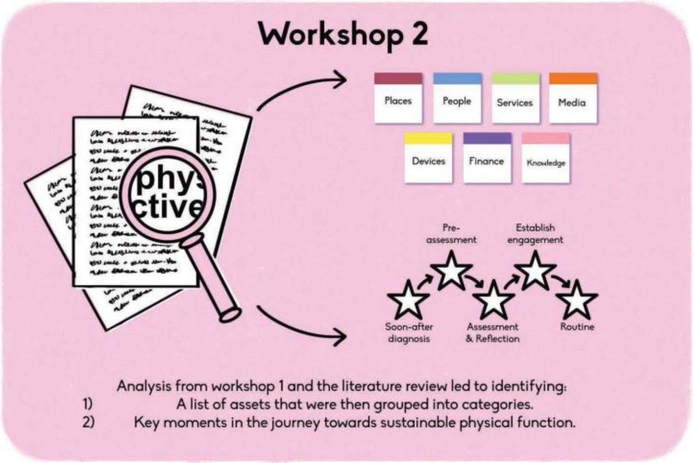
Workshop 2
Places
People
Services
Media
Devices
Finance
Knowledge
Pre-assessment
Establish engagement
Soon-after diagnosis
Assessment & Reflection
Routine
Analysis from workshop 1 and the literature review led to identifying:
1) A list of assets that were then grouped into categories.
2) Key moments in the journey towards sustainable physical function.

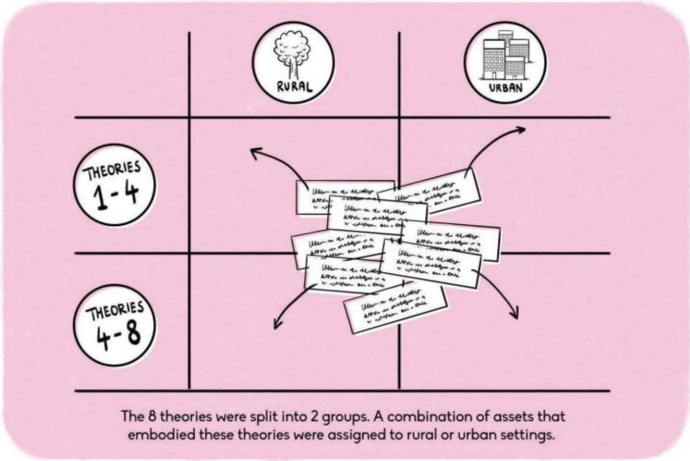
RURAL
URBAN
THEORIES 1-4
THEORIES 4-8
The 8 theories were split into 2 groups. A combination of assets that embodied these theories were assigned to rural or urban settings.

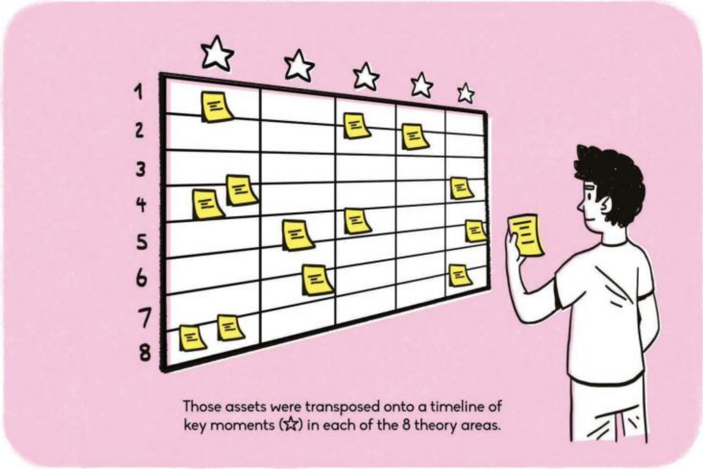
1
2
3
4
5
6
7
8
Those assets were transposed onto a timeline of key moments (☆) in each of the 8 theory areas.

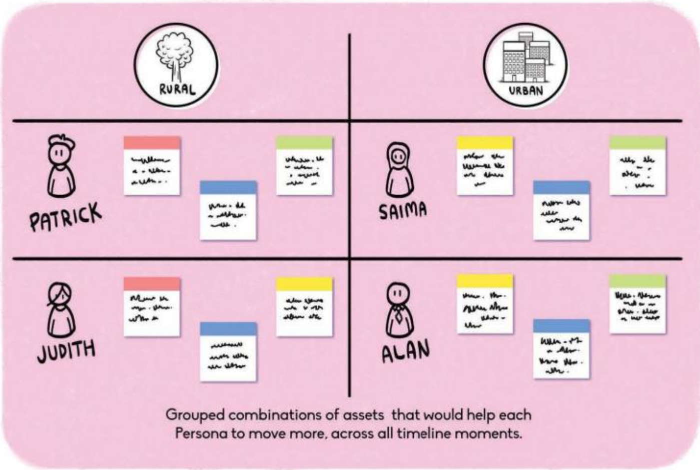
RURAL
URBAN
PATRICK
SAIMA
JUDITH
ALAN
Grouped combinations of assets that would help each Persona to move more, across all timeline moments.

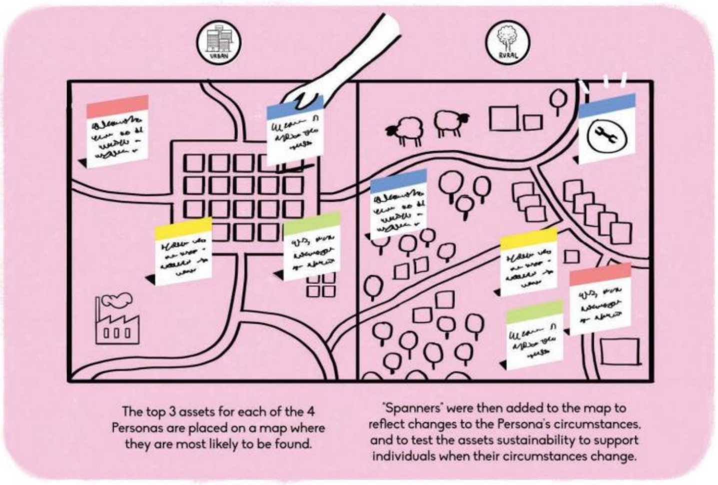
The top 3 assets for each of the 4 Personas are placed on a map where they are most likely to be found.
'Spanners' were then added to the map to reflect changes to the Persona's circumstances, and to test the assets sustainability to support individuals when their circumstances change.

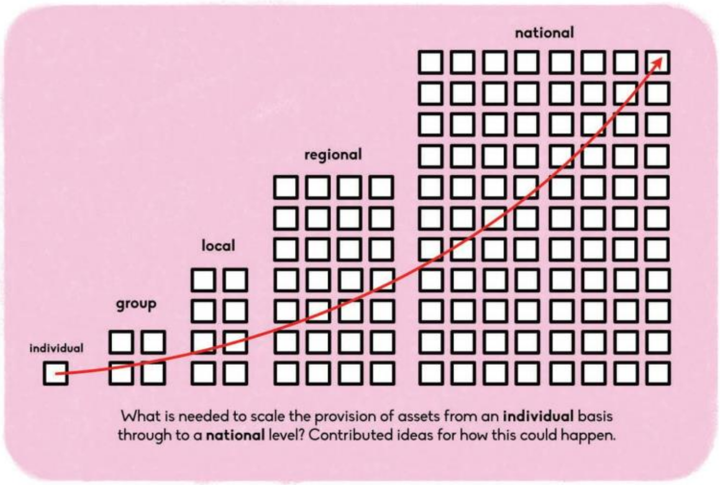
national
regional
local
group
individual
What is needed to scale the provision of assets from an individual basis through to a national level? Contributed ideas for how this could happen.

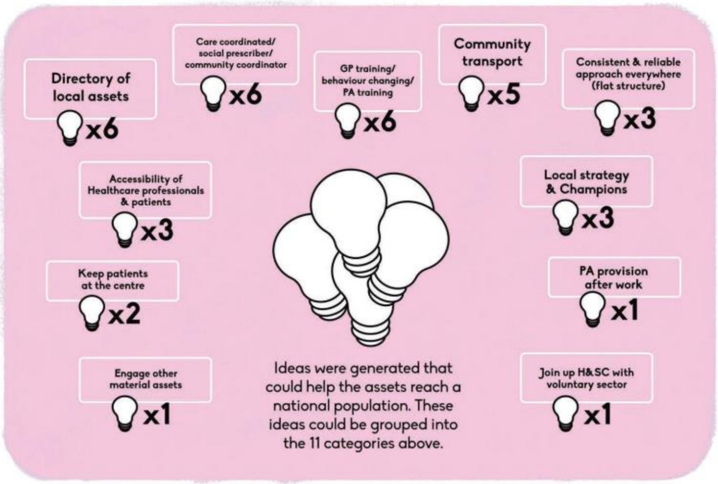
Directory of local assets x6
Care coordinated/ social prescriber/ community coordinator x6
GP training/ behaviour changing/ PA training x6
Community transport x5
Consistent & reliable approach everywhere (flat structure) x3
Accessibility of Healthcare professionals & patients x3
Local strategy & Champions x3
Keep patients at the centre x2
PA provision after work x1
Engage other material assets x1
Join up H&SC with voluntary sector x1
Ideas were generated that could help the assets reach a national population. These ideas could be grouped into the 11 categories above.

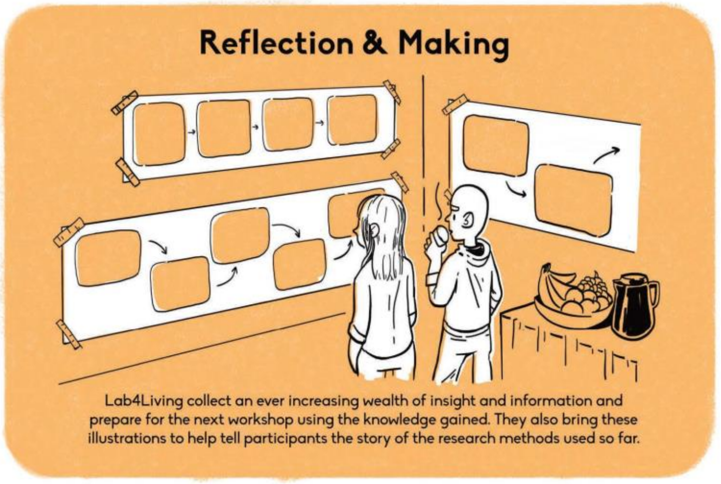
Reflection & Making
Lab4Living collect an ever increasing wealth of insight and information and prepare for the next workshop using the knowledge gained. They also bring these illustrations to help tell participants the story of the research methods used so far.

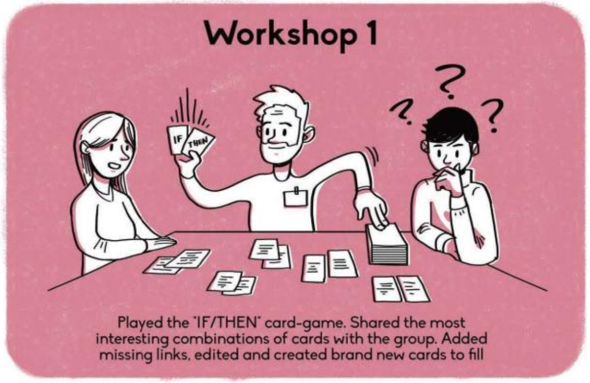

Key Findings:

- Appropriate delivery and accessible language at the first contact is vital.
- Change the cards from 'will' to 'may'. There is no 'one size fits all.'
- Social function can be linked to physical function.

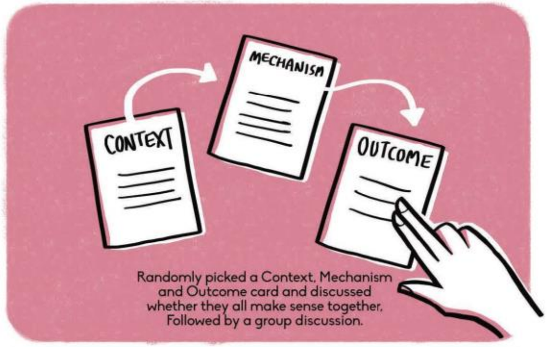

Key Findings:

- Family and personal relationships affect readiness to engage in both positive and negative ways.
- We need to ensure people don't just agree in the GP appointment without taking action afterwards.
- GPs and nurses need to be supported in developing realistic goals shared between both professional and patient.
- Focus on what is possible, rather than focussing on what has been lost.

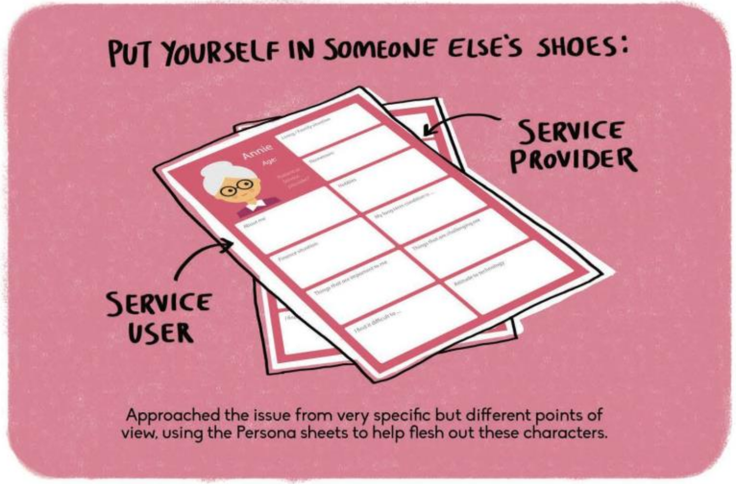

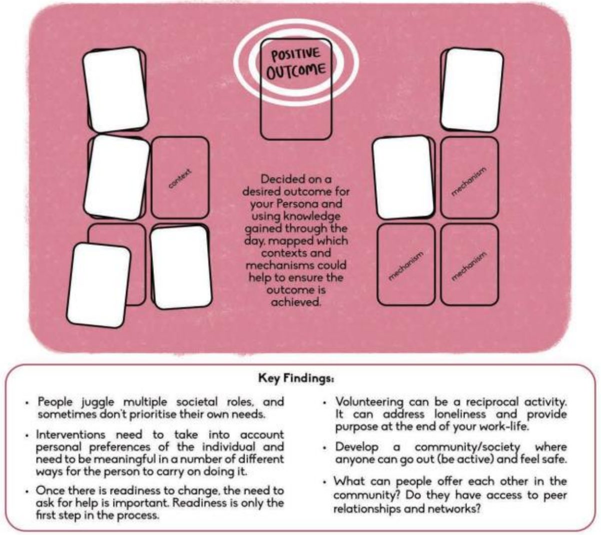
POSITIVE OUTCOME
context
mechanism
mechanism
mechanism
Decided on a desired outcome for your Persona and using knowledge gained through the day, mapped which contexts and mechanisms could help to ensure the outcome is achieved.
Key Findings:
• People juggle multiple societal roles, and sometimes don't prioritise their own needs.
• Interventions need to take into account personal preferences of the individual and need to be meaningful in a number of different ways for the person to carry on doing it.
• Once there is readiness to change, the need to ask for help is important. Readiness is only the first step in the process.
• Volunteering can be a reciprocal activity. It can address loneliness and provide purpose at the end of your work-life.
• Develop a community/society where anyone can go out (be active) and feel safe.
• What can people offer each other in the community? Do they have access to peer relationships and networks?

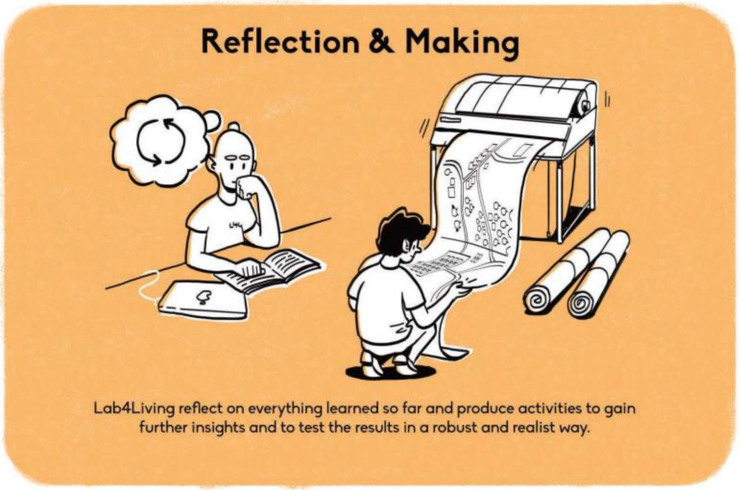
Reflection & Making
Lab4Living reflect on everything learned so far and produce activities to gain further insights and to test the results in a robust and realist way.

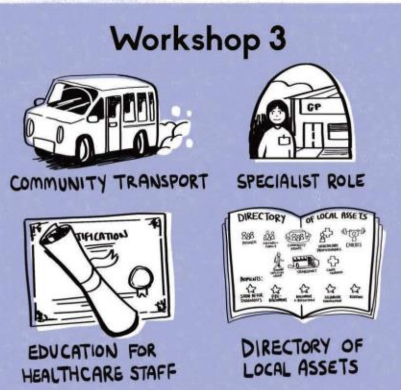

The most commonly mentioned ideas from the previous workshop were reduced into 4 distinct groups.

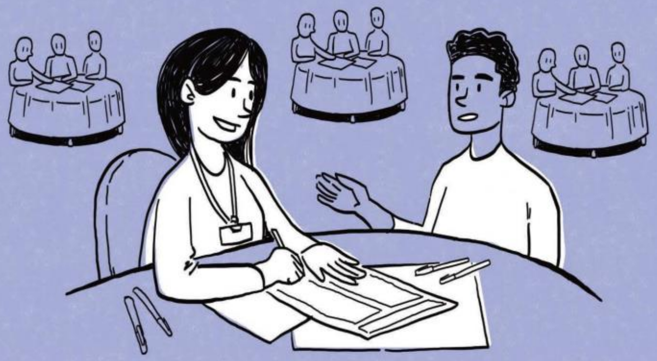

Each group started to flesh out the ideas based on their own specialist knowledge as a patient, community member or healthcare professional.

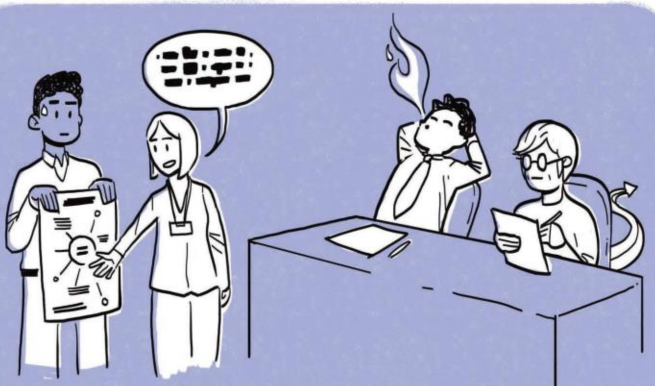

Ideas were presented to a panel of "dragons" made up of GPs, researchers and patient representatives. Ideas were challenged and questioned, exposing further areas of exploration and development.

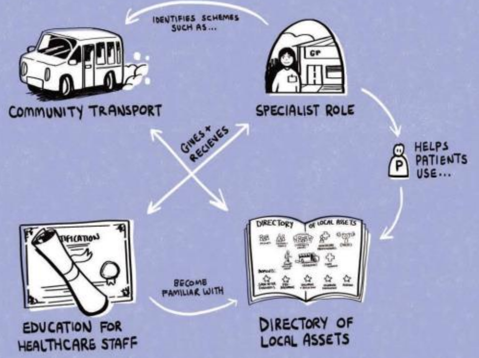

Ideas were further developed based on the dragons recommendations. During the final group discussion the boundaries between ideas began to fall away and connections began to form.

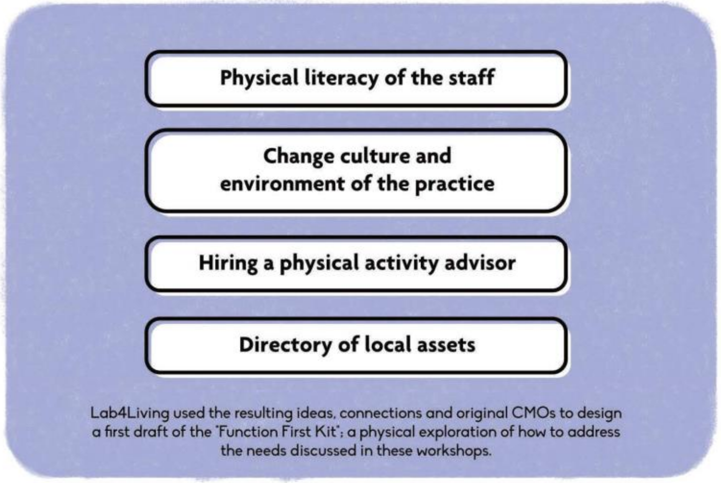
Physical literacy of the staff
Change culture and environment of the practice
Hiring a physical activity advisor
Directory of local assets
Lab4Living used the resulting ideas, connections and original CMOs to design a first draft of the "Function First Kit"; a physical exploration of how to address the needs discussed in these workshops.

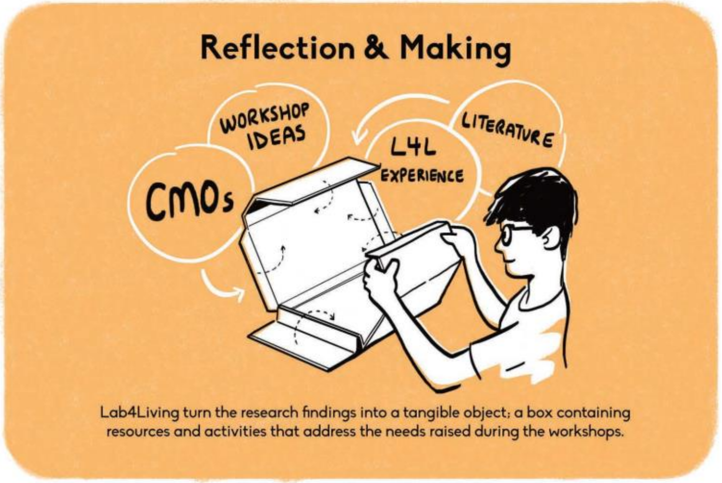
Reflection & Making
WORKSHOP IDEAS
LITERATURE
L4L EXPERIENCE
CMOs
Lab4Living turn the research findings into a tangible object; a box containing resources and activities that address the needs raised during the workshops.

Knowledge Mobilisation Session

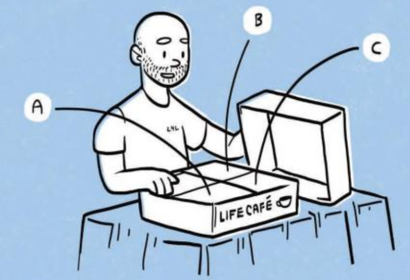

The session started with a walkthrough of previous interventions designed by Lab4Living including Life Cafe to facilitate conversations around end of life and P.E.A.R.L which provides structured reflection which fits into the busy days of healthcare professionals.

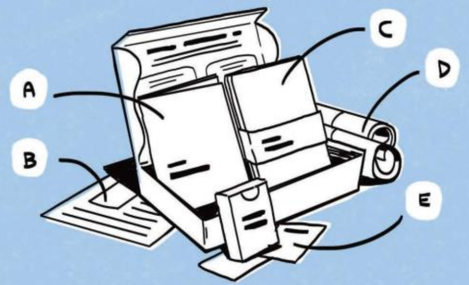

With an understanding of what is possible in terms of physical interventions, Lab4Living presented the Function First box in it's initial form, talking through each part, and how it links back to the co-production which has taken place so far.

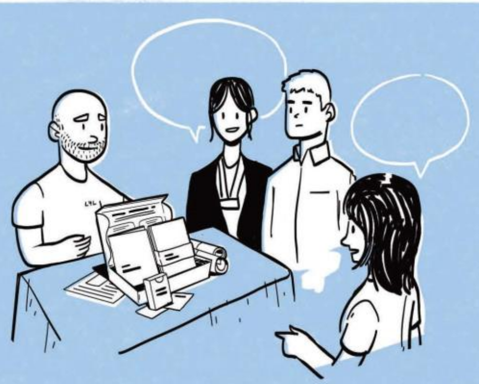

After an introduction to the intervention, a group discussion took place to further analyse, improve and add to the materials designed so far.

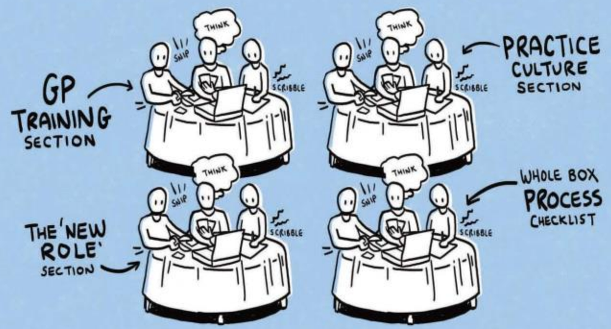

The workshop attendants split into smaller groups and each took a different area of focus. Alongside the designers at Lab4Living they created new content and resources.

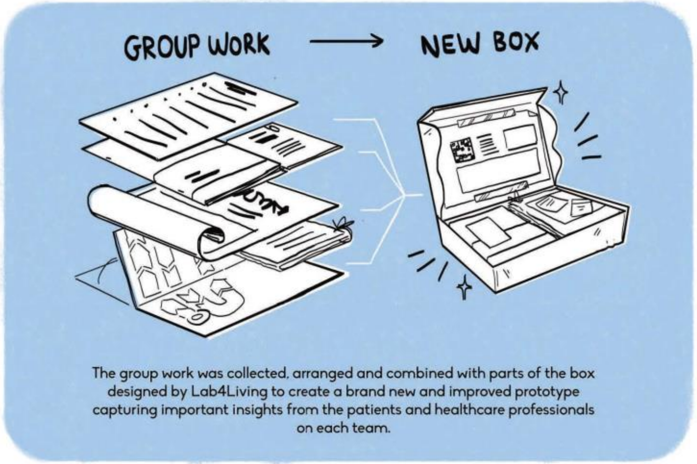
GROUP WORK → NEW BOX
The group work was collected, arranged and combined with parts of the box designed by Lab4Living to create a brand new and improved prototype capturing important insights from the patients and healthcare professionals on each team.

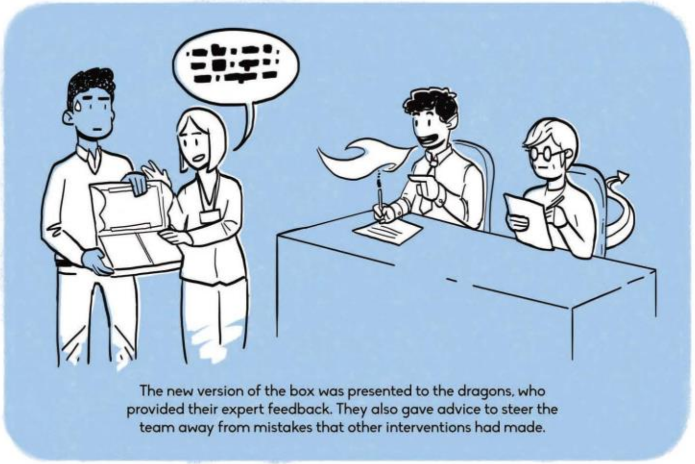
The new version of the box was presented to the dragons, who provided their expert feedback. They also gave advice to steer the team away from mistakes that other interventions had made.

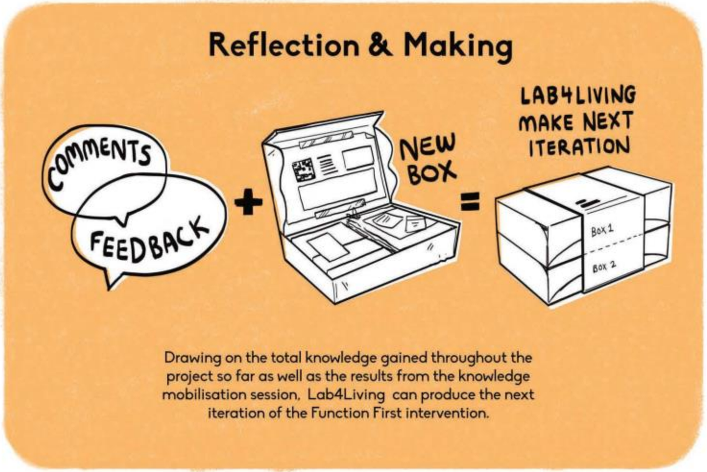
Reflection & Making
COMMENTS
FEEDBACK
+
NEW BOX
=
LAB4LIVING MAKE NEXT ITERATION
Drawing on the total knowledge gained throughout the project so far as well as the results from the knowledge mobilisation session, Lab4Living can produce the next iteration of the Function First intervention.

For peer review only

Supplementary figure 2: Example models built by participants in the theory-building workshops to reflect on and describe their interpretation of: A) what physical function meant to them, B) how they maintained physical function and C) an example of a ‘shared landscape’.

A B

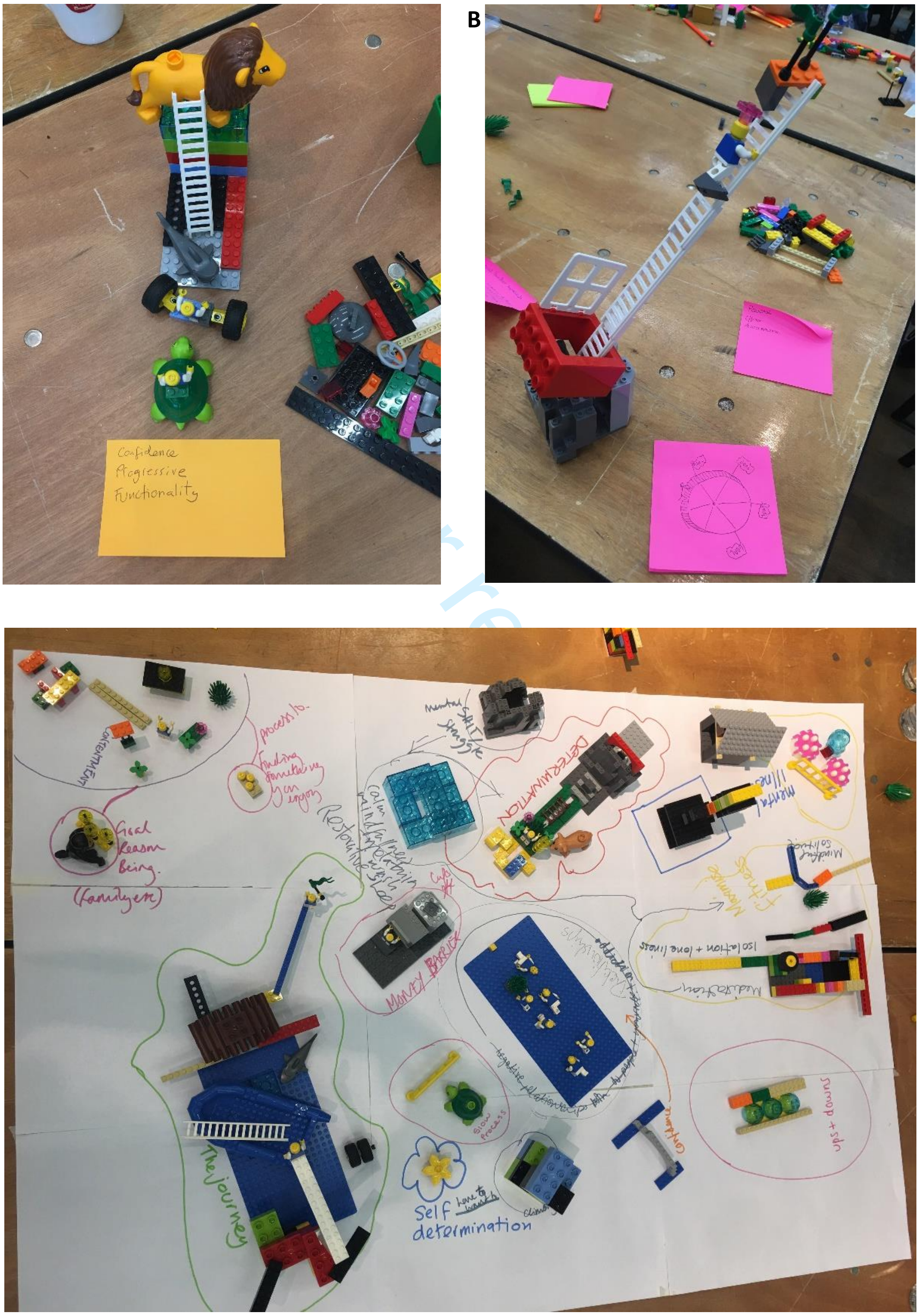

C

Supplementary table 1: Stakeholder contribution to the theory-building workshops and telephone interviews, and cumulative contribution of individual stakeholders to the co-design and knowledge mobilisation workshops. * = also took part in an individual interview (n = 1 public contributor, n = 1 representative from relevant third sector organisation, who also had a long-term condition, n = 1 GP, n = 1 researcher). ** = also took part in co-design workshops 2 and 3 and the knowledge mobilisation workshop (n = 1). *** = also took part in a co-design workshop 3 and the knowledge mobilisation workshop. # = member of research team. Co-Chief Investigators BL (health services researcher) and NW (academic GP) contributed as stakeholders to all study workshops.

Theory-building workshops and stakeholder interviews					Co-design and knowledge mobilisation			
**Stakeholder representation**	Theory-building workshop 1 (N = 10)	Theory-building workshop 2 (N = 13)	Telephone interview (N = 10)	**Stakeholder representation**	Co-design workshop 1 (N = 9)	Co-design workshop 2 (N = 14)	Co-design workshop 3 (N = 11)	Knowledge mobilisation workshop (N = 12)
**Public contributor, long-term condition**	n = 5*	n = 6**	n = 3	**Public contributor 1, long-term condition**	✓	✓	✓	✓
**GP**	n = 1	n = 1*	n = 3	**Public contributor 2, long-term condition**	✓	✓	-	✓
**Leisure centre manager**	n = 1	-	-	**Public contributor 3, long-term condition**	✓	-	-	-
**Sport and outdoor recreation division of local council**	n = 1	-	-	**Public contributor 4, long-term condition**	-	✓	✓	✓

For peer review only

**Health and social care public representation group**	n = 1	-	-
**Practice manager**	-	n = 1	-
**Researcher, social care**	-	n = 1	-
**Occupational therapist**	-	n = 1	-
**Third sector organisation**	n = 1*	n = 1	-
**Physiotherapist**	-	n = 1	-
**Engagement officer**	-	n = 1	-
**Primary care practice nurse**	-	-	n = 2
**Primary care healthcare assistant**	-	-	n = 1
**Researcher, pedagogy**	-	-	n = 1***

**Public contributor 5, long-term condition#**	-	✓	✓	✓
**Public contributor 6, long-term condition**	-	✓	-	-
**Public contributor 7, long-term condition**	-	✓	-	-
**General Practitioner**	-	✓	✓	✓
**Practice nurse**	-	✓	-	-
**Practice manager**	-	✓	-	-
**Physician associate**	✓	-	✓	✓
**Physiotherapist**	-	✓	-	-
**Primary care cluster lead, coordinator of voluntary services**	✓	✓	✓	-
**Exercise referral scheme co-ordinator**	✓	✓	✓	-

For peer review only

**Life coach**	-	✓	-	-
**Researcher, pedagogy***	-	-	✓	✓
**Researcher, physical activity promotion**	-	-	-	✓
**Researcher, clinical exercise physiology#**	-	✓	-	-
**Researcher, medical sociology#**	-	-	✓	✓
**Researcher, health psychology#**	✓	-	✓	✓
**Researcher, nursing, rehabilitation and implementation#**	✓	-	-	-
**Researcher, sports physiology#**	✓	-	✓	✓
**Researcher, information science#**	-	-	-	✓
			**5 ‘Dragon’s’/independent advisors included representation from:**	**4 ‘Dragon’s’/independent advisors included representation from**

					• 2 public contributors with long-term conditions<br>• General practice<br>• Public Health<br>• Physical activity promotion research	organisations and professional bodies relevant to:<br>• General practice<br>• Public health<br>• Nursing<br>• Physiotherapy

For peer review only

Supplementary table 2: If…Then statements

**Overarching theory areas**
**Physical literacy:** the motivation, confidence, physical competence, knowledge and understanding to value and take responsibility for engagement in physical activity for life (Jones et al, 2018)
**International Classification of Functioning:** The International Classification of Functioning, Disability and Health, known more commonly as ICF, is the World Health Organisation's framework for measuring health and disability at both individual and population levels (World Health Organisation, 2019).
**If…then statements**
**Level: Local engagement outside of practice**
If people have access to a variety of physical activity opportunities, then they will be more likely to pursue opportunities to be more physically active.
If there are sufficient places on exercise referral schemes and physiotherapy clinics, then people will receive timely interventions to improve physical function
**Level: Whole practice**
If there is a culture of physical literacy in the practice then patients are more likely to receive interventions that promote physical activity
If a range of referral options are available according to patients' functional status (pyramid model) then more patients will receive appropriate interventions to enhance their physical function
If there are sufficient staff working in the practice who are trained to promote physical literacy then more patients will receive timely physical activity interventions
If staff are trained in a variety of techniques to encourage physical activity (goal-setting, coaching, motivational interviewing) then they are more likely to promote physical literacy
If the practice has good links with outside agencies/social networks providing a wide range of physical activities in the local area then more people will engage with physical activities
If there are relevant features and cues within the built environment, then physical activity and physical function will improve.
**Level: Health professional consultations with people with long-term conditions**
If physical literacy is promoted during routine primary care management of long-term conditions, then physical activity will increase and physical functioning will improve.
If consultations with people with long-term conditions consistently address physical activity and physical function, then people will be more likely to prioritise this element of management
If physical literacy is promoted in each long-term condition consultation then physical literacy will be integrated into routine care

For peer review only

If self-efficacy and goal-setting are promoted as part of routine care then physical activity interventions will be more meaningful to patients and uptake and adherence will increase.
If pacing is used in physical activity promotion, incorporating periods of rest and recuperation, then patients with low energy levels and low fitness levels are more likely to persist with physical activity interventions
**Level: Individual patients**
Physical
If physical activity advice is tailored to patients' own priorities for functioning, then they are more likely to start and continue physical activities
If a functional approach is adoped to the management of long-term conditions, then interventions will be more relevant and tailored towards functional limitations that are meaningful to the individual
Psychological
If being physically active is linked to the freedom, well-being and joy associated with having physical function, then people with long-term conditions will better identify with the reasoning for being physically active and be more likely to continue.
If enjoyment is emphasised as an important element of physical activity, then people will be more motivated to pursue physical activity opportunities and therefore maintain physical function.
If people have built an engrained physical activity 'identity' through previous experiences, then they will be more likely to be motivated to pursue physical activity opportunities
If people with long-term conditions are supported to develop contingency plans for unpredictable circumstances, then they will be more likely to be able to persist with physical activity.
If people have self-efficacy for maintaining and improving physical activity and physical function, then they will be more likely to lead a physically active lifestyle.
If anxiety about exercising is reduced (e.g. fatigue, soreness, shortness of breath, tachycardia) through educational interventions about the normal physiological effect of exercise, then people are more likely to start and continue being physically active.
If people have recently been diagnosed with a long-term condition or experienced a life event, then a window of opportunity where people are most receptive can be utilised to promote physical activity in order to maintain and improve physical function.
If low mood reduces the motivation to exercise then improving mood will increase physical activity participation
If inspiration (indirect and direct) is provded for people with long-term conditions, then this will facilitate the development of self-efficacy for improving physical activity and physical function
If people develop a sense of agency relating to physical activity and physical function, then they will be more likely to pursue physical activity opportunities.
Social

For peer review only

If people feel supported by family or friends then they are more likely to start and continue physical activity
If people perform physical activity or exercise in groups then they are more likely to continue
If people with long-term conditions are labelled/stereotyped less, then perceived and actual restrictions will reduce, encouraging improvements in physical activity and physical function

For peer review only

Supplementary table 3: Taxonomy of primary care physical activity interventions for people with long-term conditions

Type of intervention	Description	Example papers
Brief interventions	Brief verbal advice, discussion, negotiation or encouragement, with or without written or other support or follow-up. It can vary from basic advice to a more extended, individually focused discussion	**Lamming (2017) – systematic review of reviews [26]** Brief Interventions can increase self-reported physical activity in the short term, but there is insufficient evidence about their long-term impact, their impact on objectively measured physical activity, and about the factors that influence their effectiveness, feasibility and acceptability.
Telephone interventions	Physical activity interventions delivered predominantly over the telephone	**Goode (2012) – systematic review [27]** Findings provide continuing strong evidence supporting the efficacy of telephone-delivered interventions to promote physical activity.
Online/'eHealth' interventions	Using information and communication technology for health.	**Muellmann (2018) – systematic review [28]** 'eHealth' interventions can effectively promote PA in older adults aged 55 years and above in the short-term, while evidence regarding long-term effects and the added benefit of eHealth compared to non-eHealth intervention components is still lacking.
Exercise referral schemes	The practice of referring a person from primary care to a qualified exercise professional who uses relevant medical information about the person to develop a tailored programme of physical activity usually lasting from 10 to 12 weeks.	**Campbell (2015) – systematic review [29]** Compared with usual care, exercise referral schemes had a small effect in increasing the proportion of individuals achieving 90–150 min of at least moderate intensity activity per week.
Community 'navigators'	Appropriately trained individuals who can guide patients towards local physical activity opportunities.	**Leenaars 2017 – Care Sport Connectors (CSCs) [30]** Introduced in 2012 in the Netherlands and seem to hold the promise of improving collaboration between the primary care and the physical activity sector, especially because the roles that CSCs perceive themselves as having seem to be directed at eliminating barriers in this connection.
Referral to exercise specialists (e.g.	Referral to a qualified exercise physiologist from primary care (implemented in Australia)	**Ewald 2018 – RCT to establish cost-effectiveness [31]** Referral to an exercise physiologist increased physical activity, which persisted at 9 month

For peer review only

exercise physiologists)		follow-up. Coaching achieved a modest increase in activity equivalent to 10 minutes walking per day, at a cost of AUD$245 (approx. £150) per person. Face-to-face and telephone counselling were both effective. Note: a change in quality of life not observed so could not estimate utility.
Intervention delivery by existing primary care staff	When primary care professionals (e.g. practice nurses, healthcare assistants) deliver physical activity interventions	**Williams et al (2019) – A mixed-methods treatment fidelity assessment [32]**<br>Two practice nurses and six health care assistants delivered a theory-based walking intervention to 63 patients in their own practices. High levels of fidelity of delivery were demonstrated. However, patient-, provider-, and component-level factors impacted on treatment delivery and receipt.
Physical activity 'pathways'	'Let's Get Moving'; a UK-based attempt at embedding physical activity promotion into routine primary care, recommended by Public Health England for commissioning at a local level by Primary Care Trusts within NHS England	**Bull 2008, 2010 – Feasibility pilot study and process evaluation [33, 34]**<br>• Recruitment low, particularly in practices recruiting opportunistically vs. disease register<br>• Multiple promotion strategies needed<br>• Active patients did not move through 'exit' pathway as intended<br>• Professionals modified intervention to meet time available<br>• Needs better integration with existing referral pathways (e.g. ERS)<br>• Inventory of local opportunities would be helpful<br>**Boehler 2011 – 'the costs of changing physical activity behaviour' [35]**<br>Disease register screening is more costly than opportunistic patient recruitment (£53 vs £191). However, additional costs come with a higher completion rate and better outcomes in terms of behavioural change in patients completing the care pathway.<br>**Department of Health commissioning guidance (2012) [36] –** includes resources and protocols for implementation<br>**Loughren, 2014 – 'Let's Get Moving' Physical Activity Care Pathway (Gloucestershire) [37]**<br>Factors determining implementation success:<br>• Time required to deliver lifestyle counselling<br>• Integration/competition with existing ERS

For peer review only

		• On-going support for deliverers in coordination<br>**Barrett 2017 – Feasibility in primary care physiotherapy [38]**<br>Concluded to be a clinically feasible resource to primary care physiotherapists with some modifications and with the support of additional resources (i.e. professional training and integration with existing schemes)
Practice-wide initiatives	Active Practice Charter promoted by the Royal College of General Practitioners	**Active Practice Charter (2019) [39]**<br>The Royal College of GPs and Sport England have launched the Active Practice Charter to inspire and celebrate GP practices that are taking steps to increase activity and reduce sedentary behaviour in their patients and staff.
Community initiatives adopted by primary care	*Parkrun* practice	**Quirk and Haarke (2019) [40]** - *Parkrun* launched a project called PROVE in 2016 to engage people living with long-term health conditions in England. Over the 3 year project, *Parkrun* appointed volunteer outreach ambassadors with a specialist interest in the health condition they represented whose role was to ensure *parkrun* was welcoming, supportive and inclusive. A qualitative evaluation showed that PROVE was regarded by the ambassadors (patients, carers, professionals) as important for ensuring that people with long-term health conditions can engage in physical activity and volunteering in a safe and supportive environment.
A whole system approach to embed physical activity in clinical practice	Moving healthcare professionals programme (MHPP)	**Brannan et al (2019) [41] –** The MHPP model is a partnership between Public Health England and Sport England. It has delivered face-to-face training to 17,105 healthcare professionals, embedded materials in almost three quarters of medical schools in England and overseen > 95,000 e-learning modules over two and half years. The programme aims to bring about improvements in knowledge, skills and practice. Individual elements of the model are being evaluated and further evaluation is planned to assess patient impact.

	Moving Medicine	**'Moving Medicine' [42]** was created to aid healthcare workers in integrating conversations about physical activity during routine clinical care. Moving medicine offers this online support for all patients of all ages and conditions. It also offers advice depending on the amount of time available with a patient and adjusts the conversations to these needs. Moving Medicine has the option of completing an 'online physical activity training course, which is accredited with 16 CPD points from the Faculty of Sport and Exercise Medicine (UK).
Multi-faceted interventions	Interventions involving a combination of approaches	**Avery et al (2016) [43] –** 'Movement as Medicine for Type 2 diabetes' is a multifaceted intervention informed by the theory of planned behaviour and social cognitive theory and consisted of 15 behaviour change techniques. It includes an accredited online training programme for healthcare professionals who deliver a behavioural intervention for adults with type 2 diabetes. This. Intervention intensity and duration were informed by a systematic review and stakeholder work. Usability testing resolved technical problems with using the online training intervention on practice IT systems. An open pilot study of the intervention identified mechanisms to enhance intervention implementation during routine diabetes consultations.
Campaigns	Age UK Resources – 'We Are Undefeatable'	The **'We Are Undefeatable' [44]** campaign was developed by 15 leading health and social care charities. Its main aim is to support and encourage ways for individuals with a wide range of health conditions, to remain active even during times where this can be challenging.

For peer review

Realist And MEta-narrative Evidence Syntheses (RAMESES) Checklist

Section	Item	Page number
**Title**		
1.	In the title, identify the document as a realist synthesis or review	1
**Abstract**		
2.	While acknowledging publication requirements and house style, abstracts should ideally contain brief details of: the study's background, review question or objectives; search strategy; methods of selection, appraisal, analysis and synthesis of sources; main results; and implications for practice.	2
**Introduction**		
3. Rationale for review	Explain why the review is needed and what it is likely to contribute to existing understanding of the topic area.	4
4. Objectives and focus of review	State the objective(s) of the review and/or the review question(s). Define and provide a rationale for the focus of the review.	4-5
**Methods**		
5. Changes in the review process	Any changes made to the review process that was initially planned should be briefly described and justified.	13
6. Rationale for using realist synthesis	Explain why realist synthesis was considered the most appropriate method to use.	4
7. Scoping the literature	Describe and justify the initial process of exploratory scoping of the literature.	6
8. Searching processes	While considering specific requirements of the journal or other publication outlet, state and provide a rationale for	6

For peer review only

	how the iterative searching was done. Provide details on all the sources accessed for information in the review. Where searching in electronic databases has taken place, the details should include, for example, name of database, search terms, dates of coverage and date last searched. If individuals familiar with the	
9. Selection and appraisal of documents	Explain how judgements were made about including and excluding data from documents, and justify these.	6
10. Data extraction	Describe and explain which data or information were extracted from the included documents and justify this selection.	6
11. Analysis and synthesis processes	Describe the analysis and synthesis processes in detail. This section should include information on the constructs analysed and describe the analytic process.	6
**Results**		
12. Document flow diagram	Provide details on the number of documents assessed for eligibility and included in the review with reasons for exclusion at each stage as well as an indication of their source of origin (for example, from searching databases, reference lists and so on). You may consider using the example templates (which are likely to need modification to suit the data) that are provided.	6, Figure 2
13. Document characteristics	Provide information on the characteristics of the documents included in the review.	6, Figure 2

For peer review only

14. Main findings	Present the key findings with a specific focus on theory building and testing.	7-11
**Discussion**		
15. Summary of findings	Summarize the main findings, taking into account the review's objective(s), research question(s), focus and intended audience(s).	12
16. Strengths, limitations and future research directions	Discuss both the strengths of the review and its limitations. These should include (but need not be restricted to) (a) consideration of all the steps in the review process and (b) comment on the overall strength of evidence supporting the explanatory insights which emerged. The limitations identified may point to areas where further work is needed.	13-15
17. Comparison with existing literature	Where applicable, compare and contrast the review's findings with the existing literature (for example, other reviews) on the same topic.	14
18. Conclusion and recommendations	List the main implications of the findings and place these in the context of other relevant literature. If appropriate, offer recommendations for policy and practice.	14-15
19. Funding	Provide details of funding source (if any) for the review, the role played by the funder (if any) and any conflicts of interests of the reviewers.	15

For peer review only

BMJ Open

'Function First': How to promote physical activity and physical function in people with long-term conditions managed in primary care? A study combining realist and co-design methods.

Journal:	*BMJ Open*
Manuscript ID	bmjopen-2020-046751.R1
Article Type:	Original research
Date Submitted by the Author:	03-May-2021

Complete List of Authors:	Law, Rebecca-Jane; Bangor University, North Wales Centre for Primary Care Research<br>Langley, Joseph; Sheffield Hallam University, Lab4Living<br>Hall, Beth; Bangor University, Library and Archives Services<br>Burton, Christopher; Canterbury Christ Church University, School of Allied Health Professions<br>Hiscock, Julia; Bangor University, North Wales Centre for Primary Care Research<br>Williams, Lynne; Bangor University, School of Healthcare Sciences<br>Morrison, Valerie; School of Psychology, Bangor University<br>Lemmey, Andrew; Bangor University, School of Sports, Health and Exercise Science<br>Lovell-Smith, Candida; Patient and Public Involvement Research Partner<br>Gallanders, John; Patient and Public Involvement Research Partner<br>Cooney, Jennifer; Bangor University, School of Sport, Health and Exercise Sciences<br>Williams, Nefyn; University of Liverpool, Health Services Research

**Primary Subject Heading**:	General practice / Family practice
Secondary Subject Heading:	Sports and exercise medicine, Research methods, Rehabilitation medicine, Health services research
Keywords:	PRIMARY CARE, QUALITATIVE RESEARCH, REHABILITATION MEDICINE, SPORTS MEDICINE

SCHOLARONE™ Manuscripts

I, the Submitting Author has the right to grant and does grant on behalf of all authors of the Work (as defined in the below author licence), an exclusive licence and/or a non-exclusive licence for contributions from authors who are: i) UK Crown employees; ii) where BMJ has agreed a CC-BY licence shall apply, and/or iii) in accordance with the terms applicable for US Federal Government officers or employees acting as part of their official duties; on a worldwide, perpetual, irrevocable, royalty-free basis to BMJ Publishing Group Ltd ("BMJ") its licensees and where the relevant Journal is co-owned by BMJ to the co-owners of the Journal, to publish the Work in this journal and any other BMJ products and to exploit all rights, as set out in our licence.

The Submitting Author accepts and understands that any supply made under these terms is made by BMJ to the Submitting Author unless you are acting as an employee on behalf of your employer or a postgraduate student of an affiliated institution which is paying any applicable article publishing charge ("APC") for Open Access articles. Where the Submitting Author wishes to make the Work available on an Open Access basis (and intends to pay the relevant APC), the terms of reuse of such Open Access shall be governed by a Creative Commons licence – details of these licences and which Creative Commons licence will apply to this Work are set out in our licence referred to above.

Other than as permitted in any relevant BMJ Author's Self Archiving Policies, I confirm this Work has not been accepted for publication elsewhere, is not being considered for publication elsewhere and does not duplicate material already published. I confirm all authors consent to publication of this Work and authorise the granting of this licence.

For peer review only

Function First - Be Active, Stay Independent, BMJ Open submission

Title:

'Function First': How to promote physical activity and physical function in people with long-term conditions managed in primary care? A study combining realist and co-design methods.

Corresponding author details:

Name: Dr Rebecca-Jane Law

Address: North Wales Centre for Primary Care Research

Bangor University

Cambrian House 2

Wrexham Technology Park

Wrexham

LL13 7YP

Email: r.law@bangor.ac.uk

Telephone: 01248 383520

Fax: N/A

Author List

1) Law, Rebecca-Jane; School of Health Sciences, Bangor University, Wrexham, Wales, UK
ORCID: 0000-0002-1435-5086
2) Langley, Joseph; Lab4Living, Sheffield Hallam University, Sheffield, England, UK
ORCID: 0000-0002-9770-8720
3) Hall, Beth; Library and Archives Services, Bangor University, Bangor, Wales, UK
ORCID: 0000-0003-4980-3720
4) Burton, Christopher; School of Allied and Public Health Professions, Canterbury Christ Church University, Kent, England, UK
ORCID: 0000-0003-1159-1494
5) Hiscock, Julia; School of Health Sciences, Bangor University, Bangor, Wales, UK
ORCID: 0000-0002-8963-2981
6) Williams, Lynne; School of Health Sciences, Bangor University, Bangor, Wales, UK
ORCID: 0000-0003-2575-9710
7) Morrison, Valerie; School of Psychology, Bangor University, Bangor, Wales, UK
ORCID: 0000 0002 4308 8976

For peer review only - http://bmjopen.bmj.com/site/about/guidelines.xhtml

8) Lemmey, Andrew Bruce; School of Sport, Health and Exercise Sciences, Bangor University, Bangor, Wales, UK

ORCID: 0000-0003-1667-4539

9) Lovell-Smith, Candida; PPI research partner

10) Gallanders, John; PPI research partner

11) Cooney, Jennifer; School of Sport, Health and Exercise Sciences, Bangor University, Bangor, Wales, UK

ORCID: 0000-0002-9828-8000

12) Williams, Nefyn Howard: Department of Health Services Research, University of Liverpool, England, UK

ORCID: 0000-0002-8078-409X

Word count: 4908

For peer review only

Function First - Be Active, Stay Independent, BMJ Open submission

Abstract (300 words)

Objectives

To develop a taxonomy of interventions and a programme theory explaining how interventions improve physical activity and function in people with long-term conditions managed in primary care. To co-design a prototype intervention informed by the programme theory.

Design

Realist synthesis combining evidence from a wide range of rich and relevant literature with stakeholder views. Resulting context, mechanism and outcome (CMO) statements informed co-design and knowledge mobilisation workshops with stakeholders to develop a primary care service innovation.

Results

A taxonomy was produced, including thirteen categories of physical activity interventions for people with long-term conditions.

Abridged realist programme theory:

Routinely addressing physical activity within consultations is dependent on a reinforcing practice culture, and targeted resources with better coordination will generate more opportunities to address low physical activity. The adaptation of physical activity promotion to individual needs and preferences of people with long-term conditions is helpful in affecting positive patient behaviour change. Training can improve the knowledge, confidence and capability of practice staff to better promote physical activity. Engagement in any physical activity promotion programme will depend on the degree to which it makes sense to patients and professions, and is seen as trustworthy.

These statements informed the co-design of a prototype intervention to improve physical literacy amongst practice staff, describe/develop the role of a physical activity advisor who can encourage people to use local opportunities to be more active, and materials to support this behaviour change.

Conclusions

Previous physical activity interventions in primary care have had limited effect, perhaps because they only partially address factors emerging in our programme theory. Our prototype intervention addresses all the elements of this emergent theory, but needs further development and consideration alongside current schemes and contexts (including COVID-19 restrictions), and testing in a future study. The integration of realist and co-design methods strengthened this study.

For peer review only

For peer review only - http://bmjopen.bmj.com/site/about/guidelines.xhtml

Keywords

Physical activity, physical function, long-term conditions, primary care, realist synthesis, co-design, co-production

Strengths and limitations of this study

1) Co-production with stakeholders was embedded in all stages of the project to enhance the attention to context that is characteristic of a realist approach.
2) A wide range of evidence was reviewed in order to search for organisational context, characteristics of individuals, and circumstances that led to the success or failure of an interventions, focussing on evidence containing rich description where possible.
3) The iterative way in which the different data sources were integrated enhanced the depth and breadth of the findings.
4) We co-designed a set of flexible resources that embodied the programme theory, but which could adapt to different contexts and augment existing initiatives.
5) These resources need further development and refinement before they can be used in primary care consultations.

Introduction

In 2019 in the UK, more than 18 million adults over the age of 18 years had a long-term condition (i.e. 38% of the total adult population) [1]. Approximately 25% of people with one long-term condition report 'problems performing usual activities', rising to over 60% in those with three or more long-term conditions [2]. As older people accumulate more long-term conditions, they become increasingly frail [3-5]. This is one of the biggest challenges facing the health and social care systems [6].

There are known benefits of physical activity in the management of long-term conditions, including improved physical and psychosocial functioning [7-13]. However, the proportion of the adult population in England and Wales that are at least moderately active is low, [14, 15] and even lower in people with long-term conditions. There is an inverse association between habitual physical activity level and multi-morbidity [16, 17].

Primary care is well placed to empower individuals and communities to improve physical activity and function, because 90% of patients' interaction with the National Health Service (NHS) occurs in this setting [18]. However, primary care management of long-term conditions typically focusses on the

For peer review only - http://bmjopen.bmj.com/site/about/guidelines.xhtml

diagnosis and management of disease, and not on increasing physical activity. A better way for primary care to promote physical activity and reduce functional decline is needed, and is likely to involve a complex intervention. In order to understand the active ingredients of such an intervention, a method that focusses on complexity is required. A realist approach provides a contextualised, explanatory understanding of what works, for whom, in what circumstances, in what respects and over what duration [19-21]. Integrating this with co-design gives new ideas tangible form, and tests how these will work in the real world [22].

Objectives

The overall aim was to conduct a realist evidence synthesis, informing the development of a primary care intervention to promote physical activity and physical function for people with long-term conditions. Specific objectives were:

1) To produce a taxonomy of physical activity interventions that aim to reduce functional decline in people with long-term conditions managed in primary care.
2) To work with patients, health professionals and researchers to uncover the complexity associated with the range of physical activity interventions in primary care, and how these directly or indirectly affect the physical functioning of people with long-term conditions.
3) To identify the mechanisms through which interventions bring about functional improvements in people with long-term conditions, and the circumstances associated with how the interventions are organised and operate within different primary care contexts.
4) To understand the potential impacts of these interventions across primary care and other settings, such as secondary healthcare and social care, paying attention to the conditions that influence how they operate.
5) To co-produce an evidence-based, theory-driven explanatory account, in the form of refined programme theory to underpin and develop a new intervention through a co-design process with patients, health professionals and researchers.

Method

We performed a realist synthesis of literature following established methods [19, 23] to develop Context, Mechanism and Outcome (CMO) statements, with input from key stakeholders; including

For peer review only - http://bmjopen.bmj.com/site/about/guidelines.xhtml

people with long-term conditions, health professionals and our study management and advisory groups. Stakeholders gave feedback on the emerging theories based on their lived experience as someone with a long-term condition, health professional or researcher.

Co-production was embedded throughout the following five phases over an 18 month period: 1) participatory theory-building workshops; 2) extended literature review; 3) co-design; 4) interviews and theory refinement and 5) knowledge mobilisation. The process was iterative, with data sources informing each other as the synthesis progressed (Figure 1). In this study, 'co-production' refers to the co-production of the whole research project with stakeholders, and 'co-design' refers to the specific activities, within the co-produced research project, which focused on designing a set of resources. The overall methods are detailed elsewhere [24, 25] and a visual summary is provided in Supplementary Figure 1.

Patient and public involvement

Five public research partners were proactively engaged throughout the project and contributed to monthly study management and quarterly external project advisory group meetings. They participated in decision-making, research activities (e.g. group analysis sessions), reviewing public-facing documents, authoring reports and providing feedback on findings as they emerged.

Participants

A stakeholder analysis enabled identification and targeting of the most relevant groups for the different stages of the synthesis and co-design [26]. It included representation from people with long-term conditions, primary care professionals, allied health professionals, third-sector organisations, council-funded initiatives, social care, policy-makers and commissioners and researchers (Supplementary table 1). Stakeholders were recruited through primary care patient engagement groups, health professional groups, and academic and research support networks. All participants gave informed consent.

1) Theory-building

Two theory-building stakeholder workshops and an early scoping search of published and grey literature developed initial ideas for programme theories. We used LEGO® SERIOUS PLAY® as a participatory method for the workshops to enable expression and creativity through building models

For peer review only

and to facilitate the sharing of experiences around physical activity and physical function (for an example, see Supplementary Figure 2). A preliminary list of 'if…then' statements was developed (Supplementary table 2) which informed the first co-design workshop, the literature search strategy and inclusion/exclusion criteria.

2) *Extended literature review*

We developed and amended an iterative systematic search strategy including search terms such as 'physical activity', 'physical function' and 'primary care' [24, 25]. We ran searches across the bibliographic databases: Medline, CINAHL, ASSIA, Soc Services Abstracts, PsycInfo and Cochrane Library. We used Covidence software [27] to coordinate the review process and apply our initial inclusion and exclusion criteria to identify potentially relevant papers (Supplementary table 3). First of all, we examined and summarised relevant systematic reviews, which informed the development of the following eight 'theory areas':

- Promoting physical literacy across the practice team;
- Framing physical activity promotion around the link between physical activity and physical function;
- Routinely assessing and promoting physical function and activity;
- Reducing time pressure by offering consultation with a credible professional;
- Linking people into existing local initiatives;
- Using behaviour change techniques;
- Tailoring advice and goals;
- Social support from others.

Our initial literature search identified 170 articles for data extraction, using bespoke data extraction forms to capture study details, findings and data relevant to the above theory areas. 73 articles were selected for final inclusion because of relevance and theoretical richness (i.e. they contained explanatory information that was detailed enough to contribute to programme theory development)[25]. We supplemented the systematic search with forward and backwards citation tracking of key articles and purposive searches of guidelines, grey literature, social prescribing and physical literacy to identify 48 additional articles (Figure 2). A total of 121 pieces of evidence were selected and used to develop the CMO statements (see Supplementary table 4 for final list of papers).

For peer review only - http://bmjopen.bmj.com/site/about/guidelines.xhtml

Whilst reviewing the literature, we developed a taxonomy of interventions to help organise the breadth of interventions available and inform the developing programme theories. The taxonomy was added to as the project progressed.

3) Interviews and theory refinement

The theory areas were explored in 'theory-refining' telephone interviews with ten stakeholders and as part of the second co-design workshop. Using the data extracted from the included papers, and through ongoing discussion within the project team and advisory group, we developed initial 'candidate' CMO statements [25]. These CMO statements were continually refined throughout the later workshops.

4) Co-design

The storyboard shown in Supplementary figure 1 provides a visual representation of how the project progressed through the different stages.

Three consecutive workshops were conducted to co-design an intervention to promote physical activity for people with long-term conditions managed in primary care. The workshops included a range of stakeholders (n = 23), including people living with long-term conditions, primary care professionals, a primary care cluster lead with a third sector perspective, a life coach, exercise referral scheme coordinator, researchers and members of the Function First research team (see Supplementary table 1). A team of design researchers facilitated the workshops, during which the emerging programme theories and project storyboard, were discussed and presented visually and verbally to inform and remind participants of the evolving context.

Early indications of theories emerging from the literature were presented to the co-design participants using card games based on the 'if…then' statements [28] . Thereafter, the relationship between the evidence and concepts was iterative; we continuously ensured that the developing CMO statements were represented and embodied in the concepts and designed products. In addition, concepts that the co-design participants raised were explored in the literature

Using design-based activities including immersion, ideation and co-design [25], ideas and recommendations for service innovation, and plans for making the intervention useable, were designed collaboratively and expanded during each workshop. There were key 'deliverables' from

For peer review only - http://bmjopen.bmj.com/site/about/guidelines.xhtml

each workshop and, in between workshops, designers worked to develop ideas and provocations for the next workshop termed ‘design activities’.

5) Knowledge mobilisation

This workshop involved people with long-term conditions, primary care professionals and researchers (n = 12) and explored how best to implement the prototype intervention in different contexts, ensuring that it was desirable, feasible and viable. The design researchers presented a physical example, which embodied the top four concepts generated in the co-design phase: ‘directory of local assets’, ‘specialist role’, ‘training for health professionals’ and ‘community transport’. This prototype was designed to represent and challenge these initial ideas, bring together “the creativity of designers and people not trained in design working together in the design development process” [29], and encourage consideration from broader perspectives.

These co-developed ideas were refined through input from an external panel including representation from professional bodies for general practice, nursing and physiotherapy, and public health. Whilst detailed content was missing, demonstration of the intervention ideas illustrated how each physical element related to the refined CMO statements, creating an evidence-informed design solution (Figure 3).

Results

A taxonomy of primary care physical activity interventions for people with long-term conditions was produced and included the following categories: brief interventions [30]; telephone interventions [31]; online/’eHealth’ interventions [32]; exercise referral schemes [33]; community ‘navigators’ [34]; referral to exercise specialists (e.g. exercise physiologists) [35]; intervention delivery by existing primary care staff [36]; physical activity ‘pathways’ [37-42]; practice-wide initiatives [43]; community initiatives adopted by primary care [44]; a whole system approach to embed physical activity in clinical practice [45, 46]; multi-faceted interventions [47]; and campaigns [48] (Supplementary table 5).

This informed the development of five CMO statements explaining how the contexts and mechanisms identified lead to outcomes relevant to improving physical activity and physical function in people with long-term conditions. Each explanatory account below highlights meaning and illustrates salient points with examples of evidence from the literature and stakeholder interviews.

For peer review only - http://bmjopen.bmj.com/site/about/guidelines.xhtml

1) Changing practice culture through alignment

Primary care settings are characterised by competing demands, and improving physical activity and physical function is often not prioritised in a busy practice (C). However, if the practice team culture can be aligned to promote and support the elements of physical literacy (M), then physical activity promotion will become more routine and embedded in usual care (O).

Lack of time and competing priorities limit discussion of physical activity in primary care [49-51].

"I think physical activity unfortunately does take a bit of a back step because it's probably not seen as so important as referring somebody who is expected cancer or sorting somebody's medications out." (General practitioner, individual interview)

Competing priorities include different models of care, with the primary care management of long-term conditions typically focussing on the diagnosis of disease according to the International Classification of Diseases [52]. The International Classification of Functioning, Disability and Health places more emphasis on functional limitations in a biopsychosocial context [53]. In this context, physical activity has the potential to promote more pro-active, 'whole person' and preventive care [54, 55]. However the time and resource limitations in primary care act as barriers to the implementation of this approach [50, 56].

Physical literacy is defined as "the motivation, confidence, physical competence, knowledge and understanding to value and take responsibility for engagement in physical activities for life" [57]. Aligning practice culture with physical literacy could facilitate successful physical activity promotion. A physical literacy model for adults aged 65 years and older has been developed [58] and in the UK, the Active Practice Charter aims to enhance the culture of physical activity promotion across the primary care setting [43].

Interventions are more likely to be effective when integrated into routine practice [59, 60]. For example, the 'Let's Get Moving' pathway involved primary care embedding a physical activity promotion pathway into routine practice. However, the pathway was less successful when implemented more widely, required modifications and lacked the simplicity required to align with existing programmes [37, 41, 42]. Care is also needed to reduce the burden of routine physical activity promotion within primary care.

"But, would I want any more forms to fill in or boxes to tick or guidance that says, "If you can touch your toes and tie up your shoelaces without getting breathless you score a one…it wouldn't help me at all" (General Practitioner, individual interview).

For peer review only - http://bmjopen.bmj.com/site/about/guidelines.xhtml

In order to encourage the promotion of physical activity 'as routine', protocols, pathways and procedures are insufficient; strategies are needed that align the practice team, settings and systems with the principles of physical literacy.

2) Providing resources

Physical activity promotion in primary care is inconsistent and uncoordinated (C). If specific resources are allocated to physical activity promotion (in combination with a practice culture which is supportive) (M), then this will improve opportunities to change behaviour (O).

Despite a rise in initiatives and research [61], physical activity promotion in primary care remains inconsistent [62-67]. Exercise referral schemes have shown small effects on physical activity [33], but with low attendance and completion rates [68, 69]. There are many barriers to exercise referral at an individual, social and system-level [70]. To reduce burden on GPs, many interventions have allocated specific resources to physical activity promotion by identifying alternative professionals to deliver physical activity advice. Practice nurses [71-76], healthcare assistants [77], expert patients [78] physical activity 'coaches', 'counsellors' or 'facilitators' [79-82], exercise professionals [83], physiotherapists [84, 85], accredited exercise physiologists [86] and different combinations of allied health professionals [54, 87] have been trained to apply their existing skills and work with patients on physical activity specific goals. Furthermore, social prescribing initiatives include physical activity promotion [88-91].

In a randomised controlled trial of referral from Australian primary care to exercise physiologists, a 12-week face-to-face and telephone coaching intervention resulted in participants completing the equivalent of 10 minutes more walking per day, which persisted after nine months [35]. The Exercise as a Vital Sign programme involved a medical assistant ascertaining a patient's self-reported physical activity prior to the GP entering the room, triggering exercise related care processes [92].

Primary care resource to advise patients about insufficient physical activity during routine consultations and link them to a robust referral system of physical activity opportunities could facilitate improvements in physical activity promotion and behaviour.

3) Individual advice

People with long-term conditions have varying levels of physical function and physical activity, varying attitudes to physical activity and differing access to local resources that enable physical activity (C). If physical activity promotion is adapted to individual needs, priorities and preferences, and considers local resource availability (M), then this will facilitate a sustained improvement in physical activity (O).

For peer review only - http://bmjopen.bmj.com/site/about/guidelines.xhtml

People with long-term conditions are on a spectrum of physical functioning and physical activity levels. Some people are already active, socially integrated and able to organise their everyday lives independently, whereas others have limited independence and rely on others for care [63, 93-95]. People are at varying stages in the behaviour change process [96, 97], as explained by one participant in this study:

'There's no point in people starting to dictate to people if they're not on board with it' (Public contributor, long-term condition, individual interview).

NICE guidance highlights the importance of recognising times when people may be more open to change [98]. A variety of approaches are required to encourage people with long-term conditions to start and maintain a physically active lifestyle in a personally relevant way. The use of behaviour change techniques have been emphasised in guidance, recommending the development of goals that consider individual contexts and the impact of social support [99, 100]. One-to-one sessions can be helpful to enable initial tailoring and review, whereas group-based activities can offer another source of motivation [83]. Group consultations for people with long-term conditions have shown positive effects, indicating the potential for use when resources are limited [101, 102].

Incorporating individualised, relevant and tailored advice has the potential to maximise relevance and effectiveness. Physical activity advice needs to avoid being too demanding [103], whilst providing sufficient challenge [104]. Interventions have acknowledged the unpredictable nature of living with a long-term condition by incorporating the ability to make adjustments over time [74, 75, 77, 102, 105-108]. Tailoring should link physical activity with personally relevant, enjoyable activities that are a 'good return' for the time and effort invested [86, 109-111], for example, canine-based interventions and community football schemes [112, 113]. Alternative ways of providing advice include online [32, 114, 115] or telephone counselling [31, 116, 117] which may be preferable for some people.

4) Improving capability of practice workforce

Many primary care practice staff have a lack of knowledge and confidence to promote physical activity (C). If staff develop an improved sense of capability through education and training (M), then they will increase their engagement in physical activity promotion (O).

People with long-term conditions are familiar with primary care and typically have established trust and rapport with staff [94, 118], however staff lack knowledge due to limited training and resources. An online survey of self-selecting GPs in England, found that only 20% were familiar with the national physical activity guidelines, 26% were not familiar with any physical activity assessment tools and 55% reported that they had not undertaken any training to encourage physical activity [62]. Indeed, only

For peer review only - http://bmjopen.bmj.com/site/about/guidelines.xhtml

very limited medical curriculum time is devoted to physical activity and health [119-122]. Professionals described particular difficulties delivering motivational components, such as improving self-efficacy, which were delivered less comprehensively as a result [36, 123, 124]. They lacked confidence, knowledge and understanding about roles and responsibilities for physical activity promotion [125, 126].

Interventions such as 'Movement as Medicine' [47], 'Moving Healthcare Professionals' [45] and 'Moving Medicine' [46] have addressed this need and aim to provide more training and education for primary care health professionals. In particular, 'Moving Medicine' [46] aims to incorporate conversations about physical activity during routine care, and offers online support relevant to patients of all ages with different long-term conditions. Improved education should increase the confidence of healthcare professionals in delivering physical activity advice [127].

5) Programme credibility

If a programme is credible (C), then trust and confidence in the programme will develop (M) and more patients and professionals engage with the programme (O).

Established programmes that take place in hospitals or leisure centres, and are delivered by qualified personnel (e.g. cardiac rehabilitation or exercise referral schemes), have a high degree of credibility due to their association with the health service, relevant regulatory bodies and inclusion as part of NICE guidance [99]. GP referrals are often chosen as a strategy because recommendation from a known and trusted professional is felt to increase uptake [35, 94, 128].

A mixed-methods review of physical activity for people with osteoarthritis, found that advice was viewed as valuable if it came from a knowledgeable healthcare professional who can explain why a person should do something, tailors the advice, clearly specifies what to do and explains the benefits [118]. Active health professionals are more likely to provide better, more credible, and motivating advice to their patients [129]. Credibility can also be achieved by including peer-led elements as this tends to increase self-efficacy amongst patients receiving advice, enhance empathy and improve the likelihood of realistic advice being given [78, 130, 131]. Understanding, tolerance, taking a genuine interest, encouragement and support were also important qualities [118].

"It needs to be someone who is really qualified, got a good track record. They do the assessments...part of the assessment is talking to people for a while, not just 5 minutes and that's it" (Public contributor, long-term condition).

For peer review only - http://bmjopen.bmj.com/site/about/guidelines.xhtml

Both professionals and patients need to feel that a programme is safe [132, 133] and effective in order to engage with it [134]. Professional acceptance and implementation is more likely if an intervention is accompanied by an evaluation that determines its effectiveness and benefit [61].

Intervention co-design

A prototype multi-component intervention was co-designed, embodying the five programme theories and providing resources to promote physical activity and physical function for people with long-term conditions (Figure 4 and Table 1).

The prototype consisted of:

- Resources designed to encourage a culture of physical literacy amongst staff and within the practice.
- Suggestions for changing the physical layout of the practice and promotional materials to create an environment that encourages physical activity.
- Materials to help develop the role of a credible professional who would facilitate behaviour change during bespoke consultations with people with long-term conditions.
- Identification of community resources, which can address barriers to the uptake of physical activity, such as community transport schemes.
- Plans to develop, or adapt, an electronic directory of local physical activity opportunities, clubs and groups.

Table 1: The components of a conceptual online 'Function First' resource

**Patients/General Public**
Anyone participating in the Function First sessions could benefit from an online profile that tracks their progress, helps keep track of their follow-up consultation schedule and supports them with bespoke, personalised activity recommendations. The general public get access to the complete directory of local activities and transport.
**GP & Surgery**
This profile exists as a way for the GP to access the activity record of any patient attending the Function First group sessions. Each member of staff may also have a personal profile as a member of the general public to benefit from the recommendations and access to the physical activity directory.
**Credible Professional/Physical Activity Advisor (PAA)**
The Advisor could have the ability to edit the patient's profile or activity plan based on the recommendations made during a session. A part of these sessions could be a

For peer review only - http://bmjopen.bmj.com/site/about/guidelines.xhtml

walk-through of how the online directory works. In addition to this, it would be desirable for the Advisor to begin to grow the network of activities and transport links by fostering communication between parties.
**Community Transport**
Transport services would be able to list their service in a separate transport section of the directory. Information about the operating area, capacity, number of vehicles, accessibility options and other information can be made available here, as well as direct contact information. An added benefit of this is that transport providers are often operated by volunteers who may also benefit from running this service.
**Community Activities**
Activity providers would be able to list their service in a dedicated section of the directory. Information about the activity, intensity, cost, capacity, accessibility options and other information can be made available here, as well as direct contact information. An added benefit of this is that activities are often run by volunteers who may also benefit from running this service.

Discussion

Summary of main findings

'Function First' is the first realist evidence synthesis of physical activity promotion for people with long-term conditions managed in primary care. We developed five theoretical statements of what works, for whom and in what circumstances. From this programme theory, we co-designed flexible resources for use by a dedicated person working in primary care to promote physical activity.

Strengths and limitations

The realist approach offered a theory-driven explanation of the promotion of physical activity and function, paying particular attention to context (i.e. settings within which interventions are placed, or pre-existing factors such as motivation or organisational factors) [135]. To enhance this, we embedded co-production with stakeholders at all stages, thus incorporating the different perspectives of people with long-term conditions, primary care staff, and the systems in which they live and work.

The study was planned as a linear, sequential process, but became more iterative during the course of the study. This facilitated greater integration of the different data sources and enhanced the depth and breadth of the findings.

We carried out systematic, comprehensive and transparent literature searches to identify a wide range of evidence and used Covidence software [27] to enable team contribution to reviewing the large dataset of publications. However, whilst we aimed to identify and present the most relevant and

For peer review only - http://bmjopen.bmj.com/site/about/guidelines.xhtml

rich evidence, many publications lacked detailed descriptions of organisational context, characteristics of individuals, and circumstances that led to the success of the intervention. We also found fewer reports of negative results, or difficulties in implementation.

A realist approach generates evidence-based recommendations that are related to a specific time, place and group of stakeholders, but which may not be applicable to alternative contexts. Similarly, co-design can be criticised for being too specific; focusing on the needs of the participants in the process, resulting in personal rather than generalisable solutions. This prototype intervention has been informed by a 'core' programme theory, but has been co-designed to be adaptable to different contexts.

Following our stakeholder analysis, we set out to recruit people from a range of socioeconomic backgrounds, with differing ethnicity and attitudes, however in reality this diversity proved difficult to achieve. This could be due to the timing and location of the face-to-face workshops (e.g. during the day, at premises linked to the University), as well as self-selection bias whereby people supportive of and engaged with physical activity would be more likely to participate. This could be addressed in the future by offering alternative ways to participate from the outset, including remote methods [136] and dedicating more time and resource to reach out to diverse groups. Therefore, the current findings may not apply to a different population/set of circumstances and the developed resources need further development and refinement before application. The changes to primary care associated with the Covid-19 pandemic will need further consideration, including those related to remote consulting, practice re-organisation, use and implementation of evidence, patient behaviour and chronic disease management [137]. The need for physical activity opportunities to align with social distancing requirements and preferences, as well as mitigating against further health inequality resulting from the pandemic, will also need to be considered [138, 139].

There are many initiatives promoting physical activity, and from the outset, we desired to complement rather than compete with these. Therefore, we involved representation from relevant bodies in our activities, and included a specific search for existing initiatives and campaigns. We co-designed a set of tangible resources that embodied all five programme theories, which are designed to be flexible and augment existing initiatives.

Comparison with existing literature

Existing realist evidence syntheses within the area of physical activity promotion for people with long-term conditions have identified similar findings to the current study. For example, a realist review

For peer review only - http://bmjopen.bmj.com/site/about/guidelines.xhtml

exploring the referral of obese adults to weight management services identified contextual factors including varying patient and practitioner characteristics and competing priorities. Practice level mechanisms included changes to systems or culture, not assuming a standardised approach, and improving communication with weight management services [140]. Trusting relationships; a shared understanding of needs; capacity to address multidimensional concerns; being able to individualise approaches to needs and priorities; and flexible intervention delivery are mechanisms proposed to maximise outcomes from exercise-based interventions for people living with Chronic Obstructive Pulmonary Disease and frailty [141].

Existing evidence suggests that health-related lifestyle advisors can remove barriers to healthy behaviour and create supportive social environments, but there is limited evidence of a positive impact on health knowledge, behaviour and outcomes [142]. The physical activity advisor role described in the current study is different because the role would be underpinned by knowledge and expertise specific to physical activity for people with long-term conditions.

Locating healthcare in leisure settings can create a physical environment that re-enforces physical activity culture and supports behaviour change, improve staff and patient experience, increase collaboration and coordination between health professionals, and increase awareness of facilities. Locating physical activity advisors in primary healthcare settings, as described in the current study, may have similar advantages. However, theories explaining the challenges of co-locating services highlight that the logistics of service delivery and the inconsistency of clinical schedules [143], may need further attention. In addition, components of theories proposed to explain the behavior change practices of exercise referral practitioners (e.g. planning and training, supportive leadership, and integration between health professionals and practitioners) may also need consideration [144] alongside learning from strategies designed to combine healthcare and physical activity to create a physical activity culture across a larger population e.g. 'Move More' in Sheffield [145].

There are limited examples of applying realist methods to facilitate intervention development. In a study developing a rehabilitation intervention for elderly patients following hip fracture, three programme theories were developed: improving patient engagement by tailoring the intervention; reducing fear of falling and improving self-efficacy to exercise and perform activities of daily living; and coordination of rehabilitation delivery [146]. These informed the development of an enhanced rehabilitation intervention. 'Movement as Medicine' included stakeholder work to develop a prototype intervention [47] and the 'Choose to Move' programme in Canada used participatory methods to co-create new ways to enhance physical activity, mobility and social connectedness in older adults [147].

For peer review only - http://bmjopen.bmj.com/site/about/guidelines.xhtml

Implications for practice and research

If general medical practice in the UK is to address the low levels of physical activity and poor physical functioning of people with long-term conditions, then current practice culture needs to change. A new role of a credible professional could facilitate this, with appropriate resources and protected time, increased engagement with local providers of physical activity opportunities, and full utilisation of electronic directories developed for social prescribing. Improved undergraduate and continuing medical education about physical activity is also necessary to augment and sustain this change. The development of primary care networks, or clusters of practices, provides the opportunity for a common, shared approach. This intervention will have cost implications, but may also have direct benefits to the NHS in terms of reduced consultations and demand for services.

Addressing only some components, and not the whole of this programme theory, may reduce the effectiveness of an intervention and explain why some existing interventions have not been successful. Our co-designed prototype intervention aimed to address all components of the programme theory, but components of existing initiatives could also contribute to a future refined intervention.

A future planned research programme will further develop the prototype intervention, and assess its acceptability and effectiveness in the context of the Medical Research Council framework for evaluating complex interventions [148]. Remote co-design options, both digital and non-digital, that can be accessed electronically, or posted to individuals, may be needed to facilitate this development [136]. The refined intervention, resources and new role need to fit in with existing schemes (e.g. National Exercise Referral Scheme and Moving Medicine) and complement public health campaigns (e.g. 'We Are Undefeatable) [33, 46, 48]. They also need to be flexible enough to adapt to different general medical practice contexts and changes associated with the Covid-19 pandemic. The programme theory and developed resources are relevant to the UK NHS context but could be adapted for other healthcare systems.

Conclusions

Despite the large number of interventions promoting physical activity in primary care, physical activity levels remain low, particularly in people with long-term conditions. The limited effect of these previous interventions might be because they only partially address factors, identified as important,

For peer review only - http://bmjopen.bmj.com/site/about/guidelines.xhtml

within our programme theory. Our prototype intervention addresses all elements of this theory, but needs further development and refinement.

Ethics

This study received approval from the Healthcare and Medical Sciences Academic Ethics Committee, Bangor University (Reference 2018-16308) and NHS Wales Research Ethics Committee 5 (References 256729 and 262726).

Funding statement

This project is funded by the National Institute for Health Research (NIHR) Health Services and Delivery Research programme (17/45/22). The views expressed are those of the author(s) and not necessarily those of the NIHR or the Department of Health and Social Care.

Data availability statement

This is an evidence synthesis involving qualitative data collection and therefore the data generated is not suitable for sharing beyond that contained within this publication and the final report published in the National Institute for Health Research journals library (Health Services and Delivery Research). Further information can be obtained from the corresponding author.

Competing interests

All authors had financial support from the National Institute for Health Research (NIHR) for the submitted work; NW is a GP partner at Plas Menai Health Centre, Llanfairfechan, Wales and is member of a NIHR Health Technology Assessment programme funding committee (commissioned research); there are no other relationships or activities that could appear to have influenced the submitted work.

Contribution of authors

For peer review only - http://bmjopen.bmj.com/site/about/guidelines.xhtml

Function First - Be Active, Stay Independent, BMJ Open submission

The contributions of the authors to different aspects of this work were as follows: conceiving the study and obtaining funding; R-JL, JL, BH, CB, JH, LW, VM, AL, CL-S, NW; gathering, analysis and interpretation of data: R-JL, JL, BH, CB, JH, LW, VM, AL, CL-S, JG, JC, NW; writing the report (wholly or in part): R-JL, JL, BH, CB, JH, LW, VM, AL, CL-S, JG, JC, NW; revising the report: R-JL, JL, BH, CB, JH, LW, VM, AL, CL-S, JG, JC, NW.

Acknowledgements

We are extremely grateful to all who contributed and supported this study as stakeholders and participants through engagement in workshops, interviews and meetings across the life of the project. We would also like to acknowledge the contribution and support of the Lab4Living research team at Sheffield Hallam University: Chris Redford who created the illustrations, storyboard and the graphics for the co-designed intervention resources, all of which were imperative to the progress and outcome of this project; Rebecca Partridge who was involved in developing and facilitating the overarching creative participatory processes, including the theory building stage and the co-design stage; Remi Bec who developed and facilitated the games and participatory activities for the co-design and knowledge mobilisation workshops and; Gemma Wheeler who also facilitated a co-design workshop. We would like to thank the following members of the study external project advisory group for their ongoing support, oversight and perspectives: Adrian Edwards (Chair), Robert Van Deursen, Julie Richardson, Asan Akpan, Jeanette Thom, Malcolm Ward, Louise Williams, Freya Davies, Andrea Hughes, Alan David Pryce. The authors would also like to thank Philip Bell who was a co-applicant and gave valuable insight from a public and patient perspective during the early stages of this project, and the research support teams within Health and Care Research Wales and the North West Coast Clinical Research Network who facilitated stakeholder involvement. Finally, from the North Wales Centre for Primary Care Research, School of Health Sciences at Bangor University, we would like to acknowledge and thank: Annie Hendry and Matthew Jones, for their help preparing the final report; Natasha Hulley, Nicola Nikolic and Richard Evans, for their ongoing administrative support throughout the project.

Figure legends

Figure 1. Schematic showing the iterative, integrated flow of information through the following five phases over an 18 month period: 1) participatory theory-building workshops; 2) extended literature review; 3) co-design; 4) interviews and theory refinement; 5) knowledge mobilisation. Arrows indicate how each element informed another. The study management group and project advisory group

For peer review only - http://bmjopen.bmj.com/site/about/guidelines.xhtml

meetings continuously informed the synthesis throughout the life of the project, and both groups involved input from public members.

Figure 2. Flowchart detailing the flow of information through the different phases of the review and the purposive searches

Figure 3: Physical variations, sample content detail and an image showing how the content was deconstructed and refined as part of the workshop.

Figure 4: Design image showing the components of Boxes 1 and 2 and their relation to the CMOs

For peer review only

References

1. Office for National Statistics. *People with long-term health conditions, UK: January to December 2019*. 2020 14 July 2020].
2. NHS Digital. *Health Survey for England, 2016*. National statistics 2017 05/04/2020].
3. Xue, Q.L., *The frailty syndrome: definition and natural history.* Clin Geriatr Med, 2011. **27**(1): p. 1-15.
4. Fried, L.P., et al., *Frailty in older adults: evidence for a phenotype.* J Gerontol A Biol Sci Med Sci, 2001. **56**(3).
5. Fried, L.P., et al., *Preclinical mobility disability predicts incident mobility disability in older women.* J Gerontol A Biol Sci Med Sci, 2000. **55**(1).
6. Department of Health and Social Care. *Long Term Conditions Compendium of Information: Third Edition*. 2012 05/04/2020].
7. Puts, M.T.E., et al., *Interventions to prevent or reduce the level of frailty in community-dwelling older adults: a scoping review of the literature and international policies.* Age Ageing, 2017. **46**(3): p. 383-392.
8. Geneen, L.J., et al., *Physical activity and exercise for chronic pain in adults: an overview of Cochrane Reviews.* Cochrane Database Syst Rev, 2017. **14**(1).
9. Larun, L., et al., *Exercise therapy for chronic fatigue syndrome.* Cochrane Database of Systematic Reviews, 2016.
10. Fransen, M., et al., *Exercise for osteoarthritis of the hip.* Cochrane Database Syst Rev, 2014. **22**(4).
11. Fransen, M., et al., *Exercise for osteoarthritis of the knee: a Cochrane systematic review.* Br J Sports Med, 2015. **49**(24): p. 1554-7.
12. Ashworth, N.L., et al., *Home versus center based physical activity programs in older adults.* Cochrane Database Syst Rev, 2005. **25**(1).
13. Heiwe, S. and S.H. Jacobson, *Exercise training for adults with chronic kidney disease.* Cochrane Database Syst Rev, 2011. **5**(10).
14. NHS Digital. *Statistics on Obesity, Physical Activity and Diet, England, 2019*. 2019 05/04/2020].
15. NHS Information Centre. *Health Survey for England 2008. Physical activity and fitness.* 2009 05/04/2020].
16. Cimarras-Otal, C., et al., *Association between physical activity, multimorbidity, self-rated health and functional limitation in the Spanish population.* BMC Public Health, 2014. **14**(1170): p. 1471-2458.
17. Autenrieth, C.S., et al., *Physical activity is inversely associated with multimorbidity in elderly men: results from the KORA-Age Augsburg Study.* Prev Med, 2013. **57**(1): p. 17-9.
18. Royal College of General Practitioners. *Health Select Committee Inquiry on Management of Long-Term Conditions*. 2013 October 2019].
19. Pawson, R., et al., *Realist review - a new method of systematic review designed for complex policy interventions.* J Health Serv Res Policy,, 2005. **10**(1): p. 21-34.
20. Pawson, R., *Evidence-based policy. A realist perspective.* London: Sage Publishing, 2006.
21. Pawson, R., *The science of evaluation. A realist manifesto.* London: Sage Publishing, 2013.
22. Cox, G., *Cox Review of Creativity in Business: building on the UK's strengths*, 2005: http://grips-public.mediactive.fr/knowledge_base/view/349/cox-review-of-creativity-in-business-building-on-the-uk-s-strengths/.
23. Rycroft-Malone, J., et al., *Realist synthesis: illustrating the method for implementation research.* Implement Sci, 2012. **7**(1): p. 33.
24. Law, R.J., et al., *Promoting physical activity and physical function in people with long-term conditions in primary care: Realist synthesis with co-design (Function First)* Health Services and Delivery Research, [in press].

25. Law, R.J., et al., *'Function First—Be Active, Stay Independent'—promoting physical activity and physical function in people with long-term conditions by primary care: a protocol for a realist synthesis with embedded co-production and co-design* BMJ Open, 2020. **10**(2).
26. Spitters, H.P., et al., *Unravelling networks in local public health policymaking in three European countries - a systems analysis.* Health Res Policy Syst, 2017. **15**(1): p. 5.
27. Covidence. *Better systematic review management: Covidence systematic review software*. 2020 05/04/2020].
28. Langley, J., et al. *'Playing' with Evidence: combining creative co-design methods with realist evidence synthesis*. in *Proceedings of the 6th International Conference on Design4Health*. 2020. Amsterdam: Lab4Living, Sheffield Hallam University.
29. Sanders, E.B.N. and P.J. Stappers, *Co-creation and the new landscapes of design.* CoDesign, 2008. **4**(1): p. 5-18.
30. Lamming, L., et al., *What do we know about brief interventions for physical activity that could be delivered in primary care consultations? A systematic review of reviews.* Prev Med, 2017. **99**: p. 152-163.
31. Goode, A.D., M.M. Reeves, and E.G. Eakin, *Telephone-delivered interventions for physical activity and dietary behavior change: an updated systematic review.* Am J Prev Med, 2012. **42**(1): p. 81-88.
32. Muellmann, S., et al., *Effectiveness of eHealth interventions for the promotion of physical activity in older adults: A systematic review.* Prev Med, 2018. **108**: p. 93-110.
33. Campbell, F., et al., *A systematic review and economic evaluation of exercise referral schemes in primary care: a short report.* Health Technol Assess, 2015. **19**(60).
34. Leenaars, K.E., et al., *Exploring the impact of the care sport connector in the Netherlands.* BMC public health, 2017. **17**(1): p. 813.
35. Ewald, B., et al., *Physical activity coaching by Australian Exercise Physiologists is cost effective for patients referred from general practice.* Aust N Z J Public Health, 2018. **42**(1): p. 12-15.
36. Williams, S.L., et al., *Translating a walking intervention for health professional delivery within primary care: A mixed-methods treatment fidelity assessment.* Br J Health Psychol, 2019. **25**(1): p. 17-38.
37. Bull, F., K. Milton, and C. Boehler, *Evaluation of the Physical Activity Care Pathway London Feasibility Pilot–Final Technical Report*, 2008: https://assets.publishing.service.gov.uk/government/uploads/system/uploads/attachment_data/file/192041/Evaluation_of_the_Physical_Activity_Care_Pathway_London_Feasibility_Pilot_-_Report.pdf.
38. Bull, F.C. and K.E. Milton, *A process evaluation of a" physical activity pathway" in the primary care setting.* BMC Public Health, 2010. **10**(1): p. 463.
39. Boehler, C., et al., *The cost of changing physical activity behaviour: evidence from a "physical activity pathway" in the primary care setting.* BMC Public Health, 2011. **11**(1): p. 370-370.
40. Department of Health. *Resources for commissioning Let's Get Moving interventions* 2012 05/04/2020].
41. Loughren, E.A., C. Baker, and D. Crone, *'Let's Get Moving'Physical Activity Care Pathway (Gloucestershire) Post-Programme Evaluation Report.*, 2014: http://eprints.glos.ac.uk/2378/.
42. Barrett, E.M., J. Hussey, and C.D. Darker, *Feasibility of a physical activity pathway for Irish primary care physiotherapy services.* Physiotherapy, 2017. **103**(1): p. 106-112.
43. Royal College of General Practitioners. *RCGP Active Practice Charter*. 2019 [cited 2019; Available from: https://r1.dotdigital-pages.com/p/49LX-5IR/active-practice-charter.
44. Quirk, H. and S. Haake, *How can we get more people with long-term health conditions involved in parkrun? A qualitative study evaluating parkrun's PROVE project.* BMC Sports Sci Med Rehabil, 2019. **11**(1): p. 22.

For peer review only

45. Brannan, M., et al., *Moving healthcare professionals–a whole system approach to embed physical activity in clinical practice.* BMC Med Educ, 2019. **19**(1): p. 84.
46. Moving Medicine. *Moving Medicine*. 2018.
47. Avery, L., et al., *Systematic development of a theory-informed multifaceted behavioural intervention to increase physical activity of adults with type 2 diabetes in routine primary care: Movement as Medicine for Type 2 Diabetes.* Implement Sci, 2016. **11**(1).
48. Undefeatable, W.A. *We Are Undefeatable; About the campaign*. 2019 17 July 2020].
49. Wheeler, P.C., et al., *Primary care knowledge and beliefs about physical activity and health: a survey of primary healthcare team members.* BJGP open, 2017. **1**(2).
50. Bull, F.C.L., et al., *Beliefs and behaviour of general practitioners regarding promotion of physical activity.* Australian Journal of Public Health, 1995. **19**(3): p. 300-304.
51. Melillo, K.D., et al., *Perceptions of nurse practitioners regarding their role in physical activity and exercise prescription for older adults.* Clinical excellence for nurse practitioners : the international journal of NPACE, 2000. **4**(2): p. 108-116.
52. World Health Organization, *ICD-10: international statistical classification of diseases and related health problems: tenth revision.* 2004.
53. World Health Organisation, *International classification of functioning, disability and health.* 2001.
54. Richardson, J., et al., *Monitoring physical functioning as the sixth vital sign: Evaluating patient and practice engagement in chronic illness care in a primary care setting - A quasi-experimental design.* BMC Family Practice, 2012. **13**.
55. Bierman, A.S., *Functional status, the sixth vital sign.* Journal of General Internal Medicine, 2001(16): p. 785-786.
56. Arden, K., F. Fatoye, and G. Yeowell, *Evaluation of a rolling rehabilitation programme for patients with non-specific low back pain in primary care: an observational cohort study.* Journal of Evaluation in Clinical Practice, 2017. **23**(2): p. 272-278.
57. International Physical Literacy Association. *Physical literacy can be described as the motivation, confidence, physical competence, knowledge and understanding to value and take responsibility for engagement in physical activities for life*. 2017; Available from: https://www.physical-literacy.org.uk/.
58. Jones, G.R., et al., *Development of a physical literacy model for older adults–a consensus process by the collaborative working group on physical literacy for older Canadians.* 2018.
59. Coulter, A., et al., *Personalised care planning for adults with chronic or long-term health conditions.* Cochrane Database Syst Rev, 2015. **3**(3).
60. Smith, S.M., et al., *Interventions for improving outcomes in patients with multi-morbidity in primary care and community settings.* Cochrane Database Syst Rev, 2012. **18**(4).
61. Lion, A., et al., *Physical activity promotion in primary care: a Utopian quest?* Health promotion international, 2019. **34**(4): p. 877-886.
62. Chatterjee, R., et al., *GPs' knowledge, use, and confidence in national physical activity and health guidelines and tools: a questionnaire-based survey of general practice in England.* Br J Gen Pract, 2017. **67**(663): p. e668-e675.
63. Hinrichs, T., et al., *General practitioner advice on physical activity: analyses in a cohort of older primary health care patients (getABI).* BMC family practice, 2011. **12**(1): p. 26.
64. Short, C.E., et al., *Physical activity recommendations from general practitioners in Australia. Results from a national survey.* Australian and New Zealand Journal of Public Health, 2016. **40**(1): p. 83-90.
65. Jorgensen, T.K., M. Nordentoft, and J. Krogh, *How do general practitioners in Denmark promote physical activity?* Scandinavian Journal of Primary Health Care, 2012. **30**(3): p. 141-146.

For peer review only

66. Omura, J.D., et al., *Primary care providers' physical activity counseling and referral practices and barriers for cardiovascular disease prevention.* Preventive Medicine, 2018. **108**: p. 115-122.
67. Lindeman, C., et al., *The extent to which family physicians record their patients' exercise in medical records: a scoping review.* BMJ open, 2020. **10**(2).
68. Murphy, S., et al., *An exploratory cluster randomised trial of a university halls of residence based social norms intervention in Wales, UK.* BMC Public Health, 2012. **12**(186): p. 1471-2458.
69. Moore, G.F., et al., *Mixed-method process evaluation of the welsh national exercise referral scheme.* Health Education 2013.
70. Din, N.U., et al., *Health professionals' perspectives on exercise referral from a process evaluation of the National Exercise Referral Scheme in Wales.* Health Education J 2015. **74**(6): p. 743-757.
71. Wilcox, S., et al., *Adoption and Implementation of Physical Activity and Dietary Counseling by Community Health Center Providers and Nurses*, 2004.
72. Croteau, K., G. Schofield, and G. McLean, *Physical activity advice in the primary care setting: results of a population study in New Zealand.* Australian and New Zealand journal of public health, 2006. **30**(3): p. 262-267.
73. Verwey, R.D.S.e., et al. *Upgrading physical activity counselling in primary care in the Netherlands*. Oxford University Press.
74. Verwey, R., et al., *A pilot study of a tool to stimulate physical activity in patients with COPD or type 2 diabetes in primary care.* Journal of Telemedicine and Telecare, 2014. **20**(1): p. 29-34.
75. Verwey, R., et al., *A monitoring and feedback tool embedded in a counselling protocol to increase physical activity of patients with COPD or type 2 diabetes in primary care: Study protocol of a three-arm cluster randomised controlled trial.* BMC Family Practice, 2014. **15**(1).
76. Verwey, R., et al., *Process evaluation of physical activity counselling with and without the use of mobile technology: A mixed methods study.* International Journal of Nursing Studies, 2016. **53**: p. 3-16.
77. Healey, E.L., et al., *The feasibility and acceptability of a physical activity intervention for older people with chronic musculoskeletal pain: The iPOPP pilot trial protocol.* Musculoskeletal Care, 2018. **16**(1): p. 118-132.
78. Van der Wulp, I., et al., *Effectiveness of peer-led self-management coaching for patients recently diagnosed with Type 2 diabetes mellitus in primary care: A randomized controlled trial.* Diabetic Medicine, 2012. **29**(10).
79. Morgan, G.S., et al., *Physical ACtivity facilitation for Elders (PACE): Study protocol for a randomised controlled trial.* Trials, 2015. **16**(1).
80. Shaw, R., et al., *Pre-exercise screening and health coaching in CHD secondary prevention: A qualitative study of the patient experience.* Health Education Research, 2012. **27**(3): p. 424-436.
81. Vanroy, J., et al., *Short- and long-term effects of a need-supportive physical activity intervention among patients with type 2 diabetes mellitus: A randomized controlled pilot trial.* PLoS ONE, 2017. **12**(4).
82. Rena Parish, T. and B.B.S. Tracie Rena Parish, *Examination of the constructs of the Transtheoretical model in patients with heart failure: a focus on physical activity readiness*, 2006.
83. Fife-Schaw, C., et al., *Comparing exercise interventions to increase persistence with physical exercise and sporting activity among people with hypertension or high normal blood pressure: Study protocol for a randomised controlled trial.* Trials, 2014. **15**(1).

For peer review only

84. McDonough, S.M., et al., *Pedometer-driven walking for chronic low back pain: A feasibility randomized controlled trial.* Clinical Journal of Pain, 2013. **29**(11): p. 972-981.
85. Comer, C., et al., *A Home Exercise Programme Is No More Beneficial than Advice and Education for People with Neurogenic Claudication: Results from a Randomised Controlled Trial.* PLoS ONE, 2013. **8**(9).
86. James, E.L., et al., *Referral for Expert Physical Activity Counseling: A Pragmatic RCT.* American Journal of Preventive Medicine 2017. **53**(4): p. 490–499.
87. Andryukhin, A., et al., *The impact of a nurse-led care programme on events and physical and psychosocial parameters in patients with heart failure with preserved ejection fraction: A randomized clinical trial in primary care in Russia.* European Journal of General Practice, 2010. **16**(4): p. 205-214.
88. Smith, T.O., et al., *Social prescribing programmes to prevent or delay frailty in community-dwelling older adults.* Geriatrics, 2019. **4**(4): p. 65.
89. Bickerdike, L., et al., *Social prescribing: less rhetoric and more reality. A systematic review of the evidence.* BMJ open, 2017. **7**(4): p. e013384.
90. Husk, K., et al., *What approaches to social prescribing work, for whom, and in what circumstances? A realist review.* Health & social care in the community., 2019.
91. Pescheny, J.V., Y. Pappas, and G. Randhawa, *Facilitators and barriers of implementing and delivering social prescribing services: a systematic review.* BMC health services research, 2018. **18**(1): p. 86.
92. Grant, R.W., et al., *Exercise as a vital sign: A quasi-experimental analysis of a health system intervention to collect patient-reported exercise levels.* Journal of General Internal Medicine, 2014. **29**(2): p. 341-348.
93. Department of Health, P.A., Health Improvement and Protection, *Start Active, Stay Active: A report on physical activity from the four home countries' Chief Medical Officers (Now updated)*, 2011: https://assets.publishing.service.gov.uk/government/uploads/system/uploads/attachment_data/file/830943/withdrawn_dh_128210.pdf.
94. Hinrichs, T., et al., *Effects of an exercise programme for chronically ill and mobility-restricted elderly with structured support by the general practitioner's practice (HOMEfit) - study protocol of a randomised controlled trial.* Trials, 2011. **12**.
95. UK CMOs, *Physical activity guidelines: UK chief medical officers' report*, 2019: https://www.gov.uk/government/publications/physical-activity-guidelines-uk-chief-medical-officers-report.
96. Prochaska, J.O., C.C. DiClemente, and J.C. Norcross, *In search of how people change: Applications to addictive behaviors.* American Psychologist, 1992. **47**(9): p. pp. 1102–1114.
97. Marcus, B.H. and L.R. Simkin, *The transtheoretical model: applications to exercise behavior.* Medicine & Science in Sports & Exercise., 1994. **26**(11): p. pp. 1400–1404.
98. National Institute for Clinical Excellence, *Behaviour change: individual approaches*, 2014.
99. National Institute for Clinical Excellence, *Physical activity: exercise referral schemes*, 2014.
100. National Institute for Clinical Excellence, *Physical activity: brief advice for adults in primary care.* London: Nice public health guidance 2013: p. 44.
101. NHS Leading Change Adding Value Team. *Introducing group consultations for adults with Type 2 diabetes*. 2019 2020].
102. Åsenlöf, P., E. Denison, and P. Lindberg, *Individually tailored treatment targeting activity, motor behavior, and cognition reduces pain-related disability: A randomized controlled trial in patients with musculoskeletal pain.* Journal of Pain, 2005. **6**(9): p. 588-603.
103. Bjerk, M., et al., *A falls prevention programme to improve quality of life, physical function and falls efficacy in older people receiving home help services: Study protocol for a randomised controlled trial.* BMC Health Services Research, 2017. **17**(1).

For peer review only

104. Bearne, L.M., et al., *Feasibility of an exercise-based rehabilitation programme for chronic hip pain.* Musculoskeletal Care, 2011. **9**(3): p. 160-168.
105. Holden, M.A., et al., *Role of exercise for knee pain: What do older adults in the community think?* Arthritis Care and Research, 2012. **64**(10): p. 1554-1564.
106. Leijon, M.E., et al., *Physical activity referrals in Swedish primary health care - Prescriber and patient characteristics, reasons for prescriptions, and prescribed activities.* BMC Health Services Research, 2008. **8**.
107. Lohmann, H., V. Siersma, and N.F. Olivarius, *Fitness consultations in routine care of patients with type 2 diabetes in general practice: An 18-month non-randomised intervention study.* BMC Family Practice, 2010. **11**.
108. Prochaska, J.J., et al., *PACE Interactive Communication Technology for Behavior Change in Clinical Settings*, 2000. p. 127-131.
109. Hurley, M., et al., *Exercise interventions and patient beliefs for people with hip, knee or hip and knee osteoarthritis: A mixed methods review*, 2018, John Wiley and Sons Ltd.
110. Danielsson, L., B. Kihlbom, and S. Rosberg, *"Crawling Out of the Cocoon": Patients' Experiences of a Physical Therapy Exercise Intervention in the Treatment of Major Depression*, 2016.
111. Åsenlöf, P., E. Denison, and P. Lindberg, *Long-term follow-up of tailored behavioural treatment and exercise based physical therapy in persistent musculoskeletal pain: A randomized controlled trial in primary care.* European Journal of Pain, 2009. **13**(10): p. 1080-1088.
112. Rhodes, R.E., et al., *Increasing physical activity by four legs rather than two: systematic review of dog-facilitated physical activity interventions.* British Journal of Sports Medicine, 2020.
113. Bjerre, E.D., et al., *Community-based football in men with prostate cancer: 1-year follow-up on a pragmatic, multicentre randomised controlled trial.* PLoS medicine, 2019. **16**(10).
114. Chaplin, E., et al., *The evaluation of an interactive web-based Pulmonary Rehabilitation programme: protocol for the WEB SPACE for COPD feasibility study.* BMJ open, 2015. **5**(8): p. e008055.
115. Devi, R., J. Powell, and S. Singh, *A web-based program improves physical activity outcomes in a primary care angina population: Randomized controlled trial.* Journal of Medical Internet Research, 2014. **16**(9).
116. Weinstock, R.S., et al., *Lessened decline in physical activity and impairment of older adults with diabetes with telemedicine and pedometer use: results from the IDEATel study.* Age and Ageing, 2011. **40**(1): p. 98-105.
117. Allen, K.D., et al., *Patient and provider interventions for managing osteoarthritis in primary care: protocols for two randomized controlled trials*, 2012.
118. Forsyth, A., F.P. Deane, and P. Williams, *Dietitians and exercise physiologists in primary care: Lifestyle interventions for patients with depression and/or anxiety.* Journal of Allied Health, 2009.
119. Ward, M., *A Survey of Physical Activity in MedicalCurricula: A report of the HEPA in Health Care Settings*, 2015: HEPA Europe Working Group.
120. Dacey, M.L., et al., *Physical activity counseling in medical school education: a systematic review.* Medical education online, 2014. **19**(1): p. 24325.
121. Dunlop, M. and A.D. Murray, *Major limitations in knowledge of physical activity guidelines among UK medical students revealed: implications for the undergraduate medical curriculum.* Br J Sports Med, 2013. **47**(11): p. 718-720.
122. Weiler, R., et al., *Physical activity education in the undergraduate curricula of all UK medical schools. Are tomorrow's doctors equipped to follow clinical guidelines?* British journal of sports medicine, 2012. **46**(14): p. 1024-1026.

For peer review only

123. Jansink, R., et al., *Primary care nurses struggle with lifestyle counseling in diabetes care: a qualitative analysis.* BMC family practice, 2010. **11**(1): p. 41.
124. Sturgiss, E., et al., *Increasing general practitioners' confidence and self-efficacy in managing obesity: a mixed methods study* BMJ open 2017. **7**(1): p. e014314.
125. Rushforth, B., et al., *Barriers to effective management of type 2 diabetes in primary care: qualitative systematic review.* Br J Gen Pract, 2016. **66**(643): p. e114-e127.
126. Leemrijse, C.J., et al., *Collaboration of general practitioners and exercise providers in promotion of physical activity a written survey among general practitioners.* BMC Family Practice, 2015. **16**(1): p. 1-9.
127. Savill, B., A. Murray, and R. Weiler, *Is general practice engaged with physical activity promotion?* Br J Gen Pract, 2015. **65**(638): p. 484-485.
128. Stone, R.C. and J. Baker, *Painful choices: a qualitative exploration of facilitators and barriers to active lifestyles among adults with osteoarthritis.* Journal of Applied Gerontology, 2015. **36**(9): p. 1091-1116.
129. Lobelo, F., J. Duperly, and E. Frank, *Physical activity habits of doctors and medical students influence their counselling practices.* Br J Sports Med online, 2009. **43**(2): p. 89-92.
130. Gamboa Moreno, E., et al., *Impact of a self-care education programme on patients with type 2 diabetes in primary care in the Basque Country.* BMC Public Health, 2013. **13**(1).
131. Gamboa Moreno, E., et al., *A Pilot Study to Assess the Feasibility of the Spanish Diabetes Self-Management Program in the Basque Country.* Journal of diabetes research, 2016. **2016**: p. 9145673-9145673.
132. Walsh, J.M.E., et al., *Exercise Counseling by Primary Care Physicians in the Era of Managed Care*, 1999.
133. Hinrichs, T., et al., *Home-Based Exercise Supported by General Practitioner Practices: Ineffective in a Sample of Chronically Ill, Mobility-Limited Older Adults (the HOMEfit Randomized Controlled Trial).* Journal of the American Geriatrics Society, 2016. **64**(11): p. 2270-2279.
134. Marchal, B., et al., *Is realist evaluation keeping its promise? A review of published empirical studies in the field of health systems research* Evaluation, 2012. **18**(2): p. 192-212.
135. Timmins, P. and C. Miller, *Making evaluations realistic: the challenge of complexity.* Support for Learning 2007. **22**(1): p. 9-16.
136. Davis, A., et al., *Low-Contact Co-Design: Considering more flexible spatiotemporal models for the co-design workshop.* Strategic Design Research Journal, 2021. **14**(1): p. 124-137.
137. Khan, N., et al., *A brave new world: the new normal for general practice after the COVID-19 pandemic.* BJGP open, 2020. **4**(3): p. bjgpopen20X101103.
138. Bambra, C., et al., *The COVID-19 pandemic and health inequalities.* Journal of Epidemiology and Community Health, 2020: p. jech-2020-214401.
139. Hall G, L.D., Phillips SA, Lavie CJ, Arena R., *A tale of two pandemics: How will COVID-19 and global trends in physical inactivity and sedentary behavior affect one another?* Prog Cardiovasc Dis., 2020. **8**.
140. Blane, D.N., S. Macdonald, and C.A. O'Donnell, *What works and why in the identification and referral of adults with comorbid obesity in primary care: A realist review.* Obes Rev, 2019. **21**(4): p. e12979.
141. Brighton LJ, E.C., Man WDC, Maddocks M. 2020;, *Improving Exercise-Based Interventions for People Living with Both COPD and Frailty: A Realist Review.* Int J Chron Obstruct Pulmon Dis, 2020. **15**: p. 841-855.
142. Carr, S., et al., *An evidence synthesis of qualitative and quantitative research on component intervention techniques, effectiveness, cost-effectiveness, equity and acceptability of different versions of health-related lifestyle advisor role in improving health.* Health Technol Assess 2011. **15**(9): p. 1-284.

For peer review only

143. Grinvalds, N., et al. *What works to facilitate the promotion of physical activity in co-located healthcare and leisure settings: a realist review.* SportRχiv, 2019. DOI: https://doi.org/10.31236/osf.io/h9r8w.

144. Downey, J., et al., *Behaviour change practices in exercise referral schemes: developing realist programme theory of implementation.* BMC health services research, 2021. **21**(1): p. 335.

145. Move More. *Move More Challenge: We are World Record breakers!* 2019 accessed 13/03/2020].

146. Roberts, J.L., et al., *Development of an evidence-based complex intervention for community rehabilitation of patients with hip fracture using realist review, survey and focus groups.* BMJ Open, 2017. **7**(10): p. e014362.

147. McKay, H., et al., *Implementation of a co-designed physical activity program for older adults: positive impact when delivered at scale.* BMC Public Health, 2018. **18**: p. 1289.

148. Craig, P., et al., *Developing and evaluating complex interventions: the new Medical Research Council guidance.* BMJ, 2008. **337**: p. a1655.

For peer review only

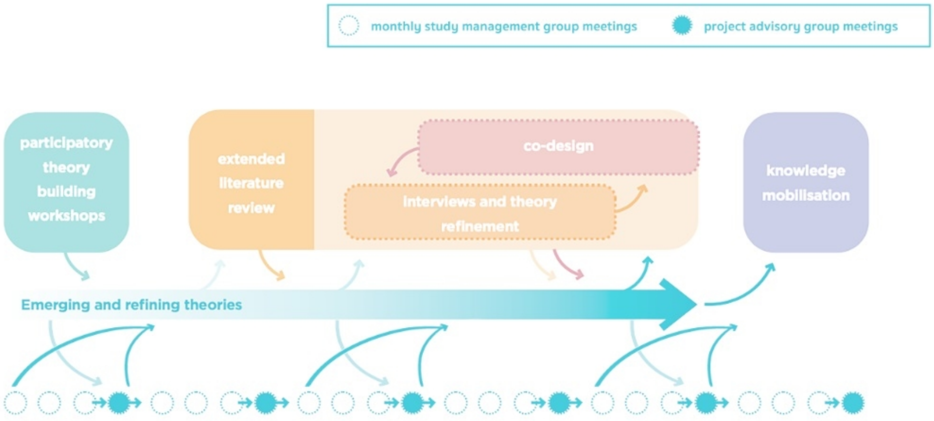

Figure 1. Schematic showing the iterative, integrated flow of information through the following five phases over an 18 month period: 1) participatory theory-building workshops; 2) extended literature review; 3) co-design; 4) interviews and theory refinement; 5) knowledge mobilisation. Arrows indicate how each element informed another. The study management group and project advisory group meetings continuously informed the synthesis throughout the life of the project, and both groups involved input from public members.

279x129mm (96 x 96 DPI)

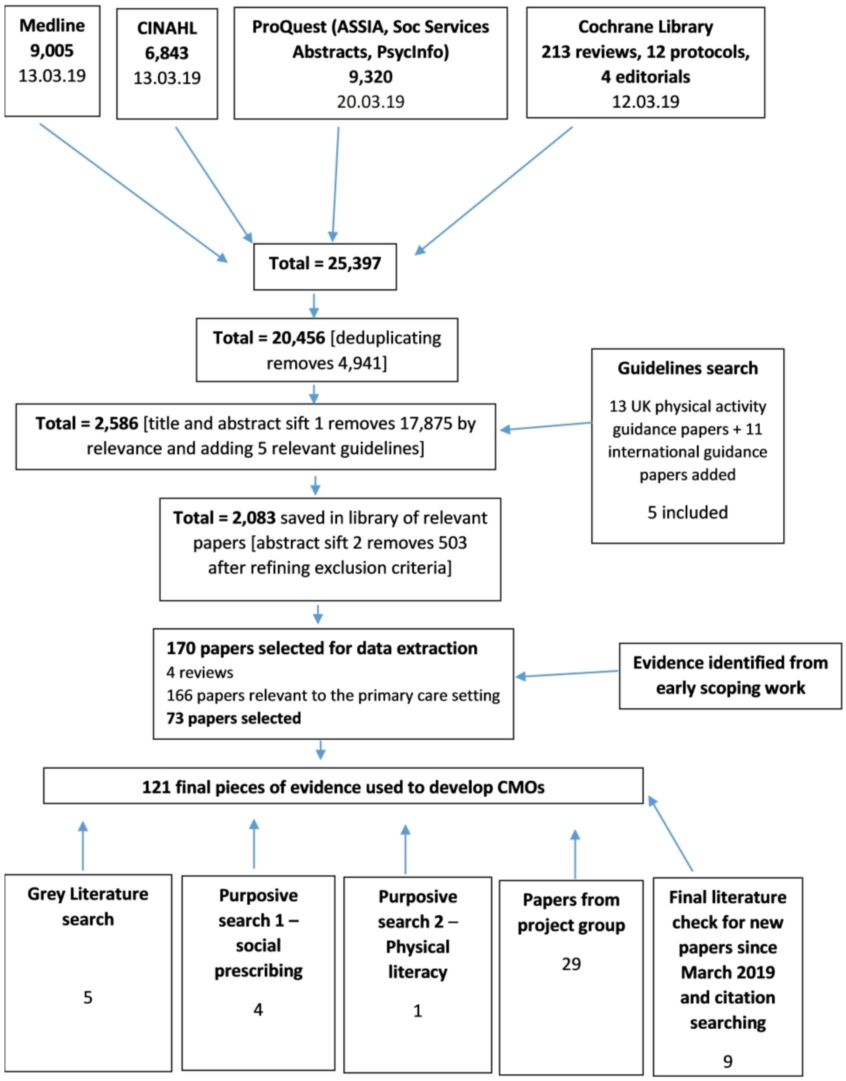

Figure 2: Flowchart detailing the flow of information through the different phases of the review and the purposive searches

1060x1514mm (96 x 96 DPI)

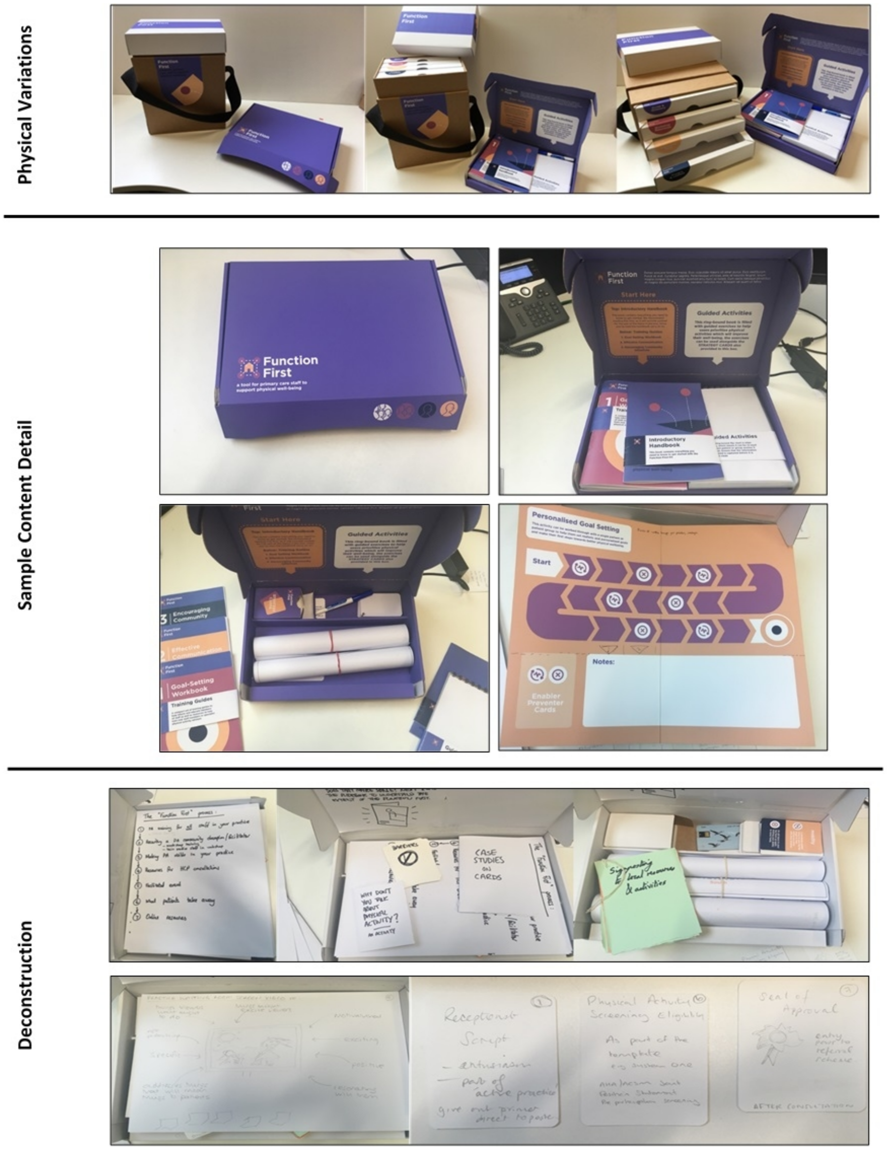

Figure 3: Physical variations, sample content detail and an image showing how the content was deconstructed and refined as part of the workshop.

260x298mm (96 x 96 DPI)

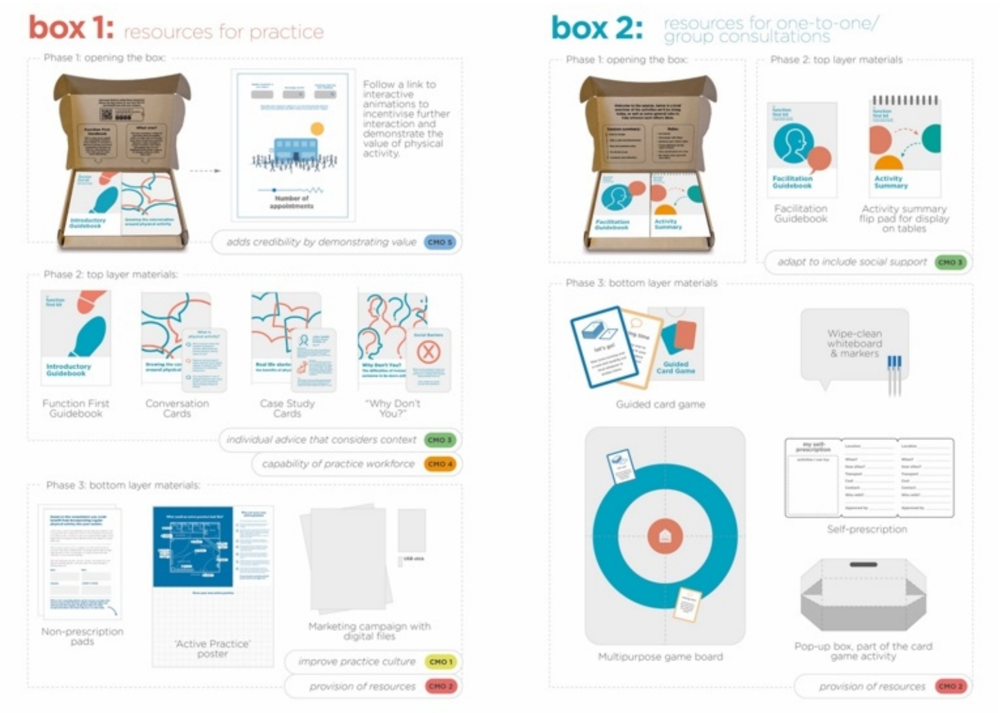

Figure 4: Design image showing the components of Boxes 1 and 2 and their relation to the CMOs

202x144mm (96 x 96 DPI)

Supplementary figure 1: Visual storyboard project summary

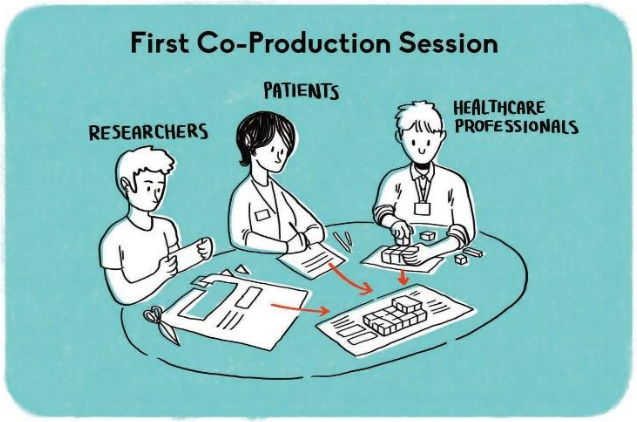

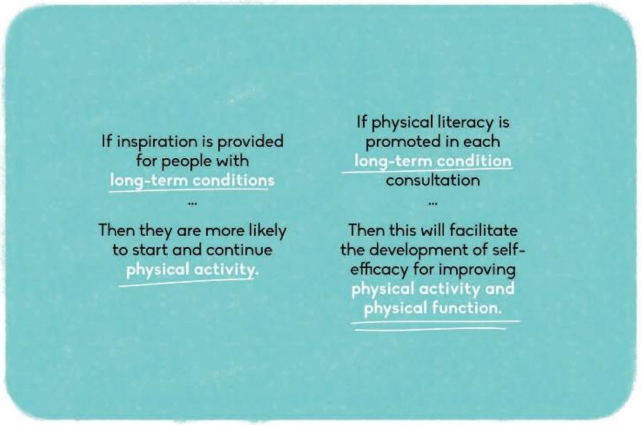

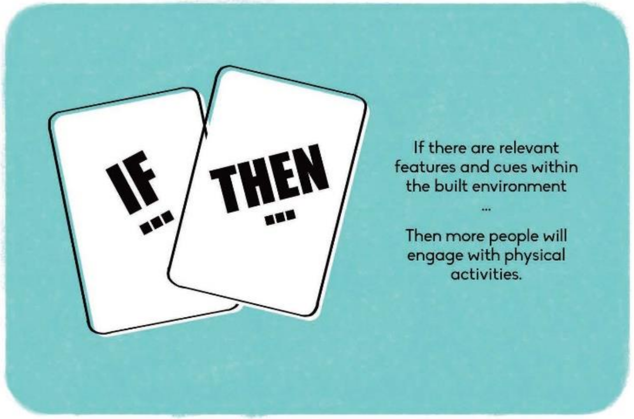

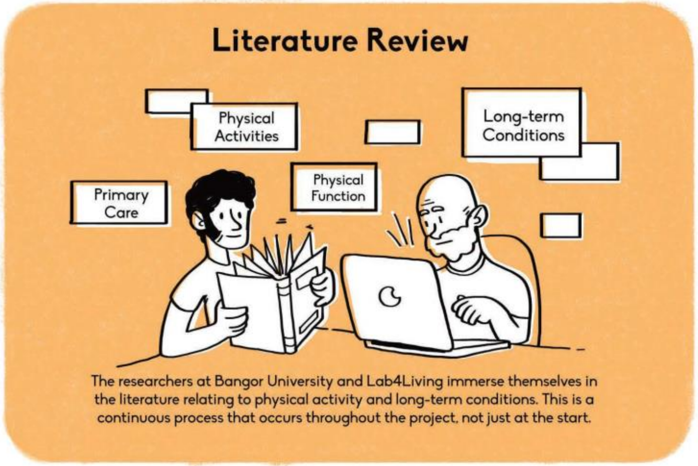
Literature Review
Physical Activities
Long-term Conditions
Primary Care
Physical Function
The researchers at Bangor University and Lab4Living immerse themselves in the literature relating to physical activity and long-term conditions. This is a continuous process that occurs throughout the project, not just at the start.

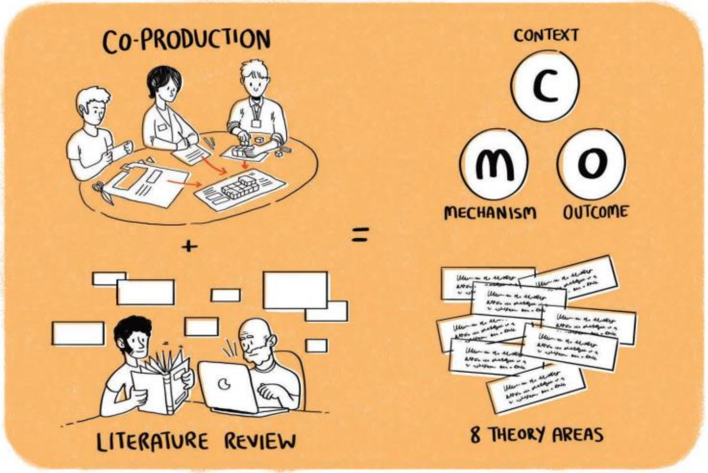
CO-PRODUCTION
CONTEXT
C
M
O
MECHANISM
OUTCOME
+
=
LITERATURE REVIEW
8 THEORY AREAS

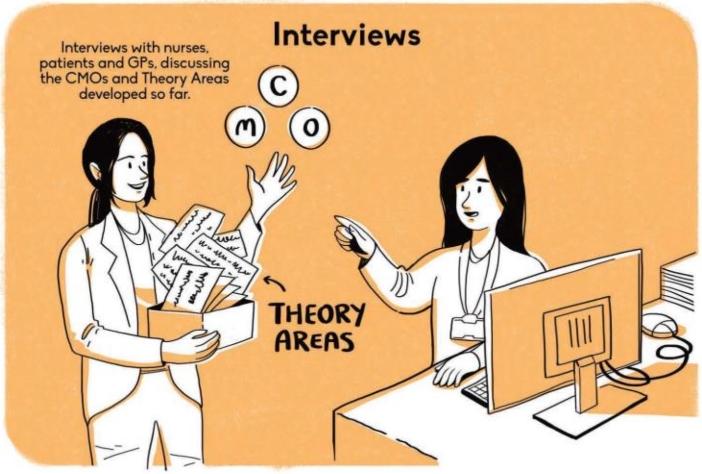
Interviews
Interviews with nurses, patients and GPs, discussing the CMOs and Theory Areas developed so far.
C
M
O
THEORY AREAS

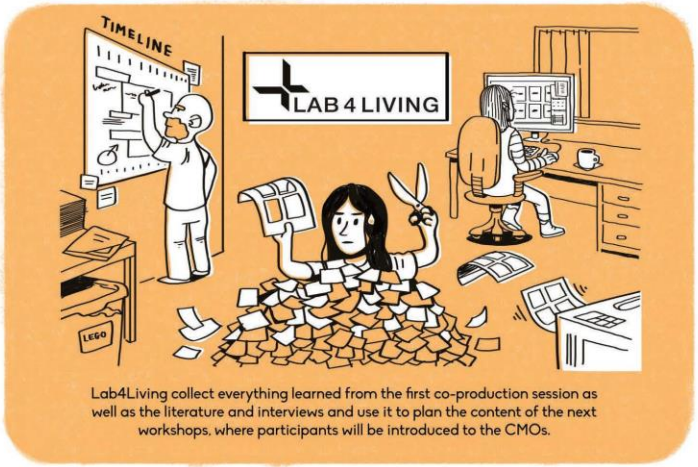
TIMELINE
LAB 4 LIVING
LEGO
Lab4Living collect everything learned from the first co-production session as well as the literature and interviews and use it to plan the content of the next workshops, where participants will be introduced to the CMOs.

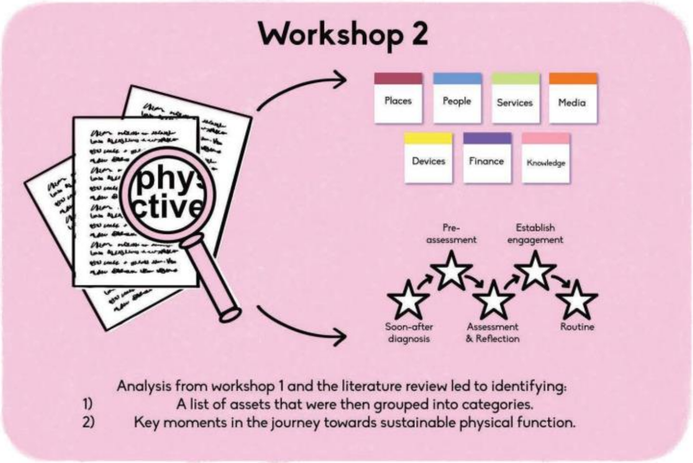
Workshop 2
Places
People
Services
Media
Devices
Finance
Knowledge
Pre-assessment
Establish engagement
Soon-after diagnosis
Assessment & Reflection
Routine
Analysis from workshop 1 and the literature review led to identifying:
1) A list of assets that were then grouped into categories.
2) Key moments in the journey towards sustainable physical function.

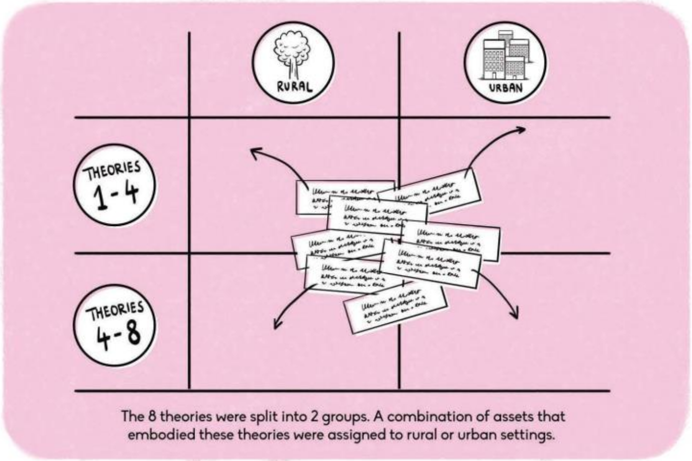
RURAL
URBAN
THEORIES 1-4
THEORIES 4-8
The 8 theories were split into 2 groups. A combination of assets that embodied these theories were assigned to rural or urban settings.

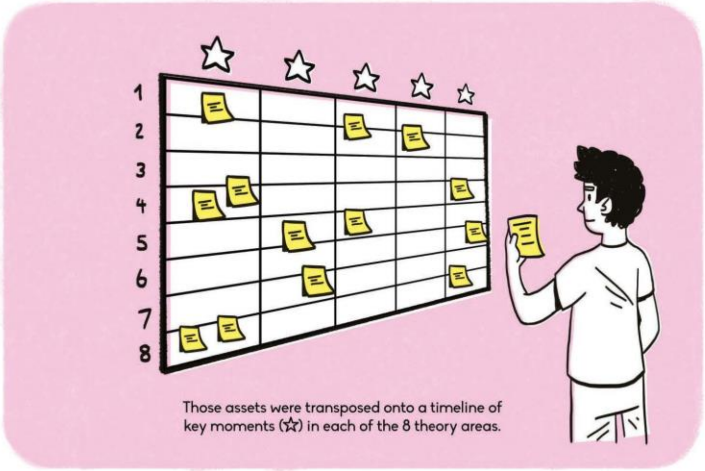
1
2
3
4
5
6
7
8
Those assets were transposed onto a timeline of key moments (☆) in each of the 8 theory areas.

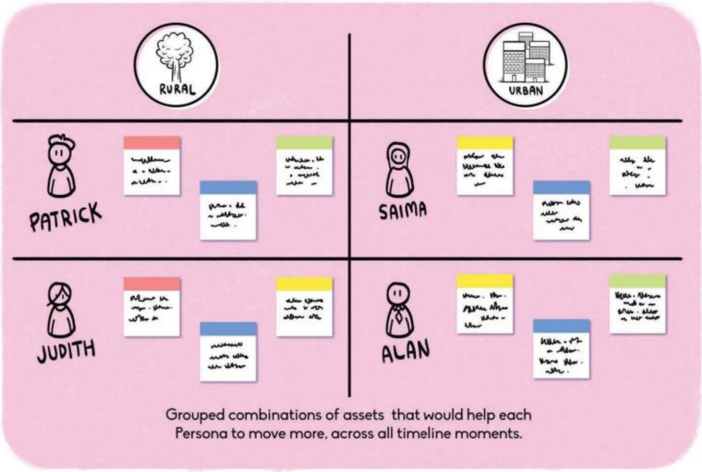
RURAL
URBAN
PATRICK
SAIMA
JUDITH
ALAN
Grouped combinations of assets that would help each Persona to move more, across all timeline moments.

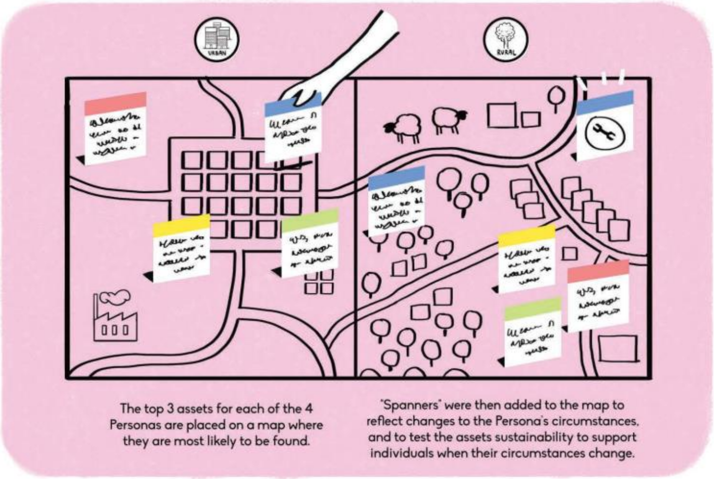
The top 3 assets for each of the 4 Personas are placed on a map where they are most likely to be found.
'Spanners' were then added to the map to reflect changes to the Persona's circumstances, and to test the assets sustainability to support individuals when their circumstances change.

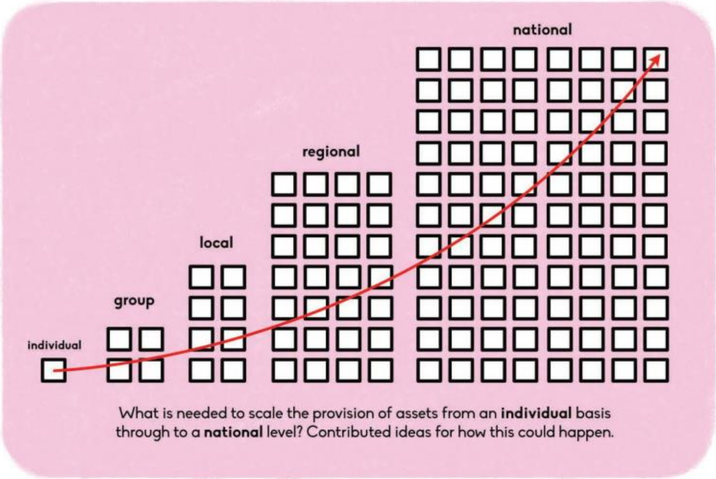
national
regional
local
group
individual
What is needed to scale the provision of assets from an **individual** basis through to a **national** level? Contributed ideas for how this could happen.

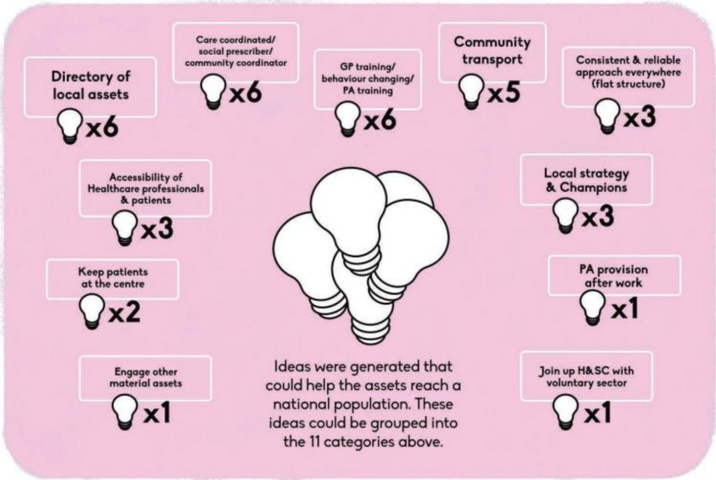
Directory of local assets x6
Care coordinated/ social prescriber/ community coordinator x6
GP training/ behaviour changing/ PA training x6
Community transport x5
Consistent & reliable approach everywhere (flat structure) x3
Accessibility of Healthcare professionals & patients x3
Local strategy & Champions x3
Keep patients at the centre x2
PA provision after work x1
Engage other material assets x1
Join up H&SC with voluntary sector x1
Ideas were generated that could help the assets reach a national population. These ideas could be grouped into the 11 categories above.

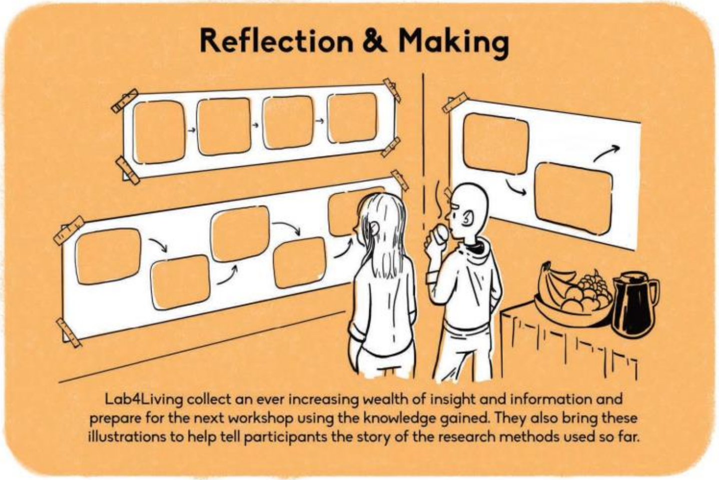
Reflection & Making
Lab4Living collect an ever increasing wealth of insight and information and prepare for the next workshop using the knowledge gained. They also bring these illustrations to help tell participants the story of the research methods used so far.

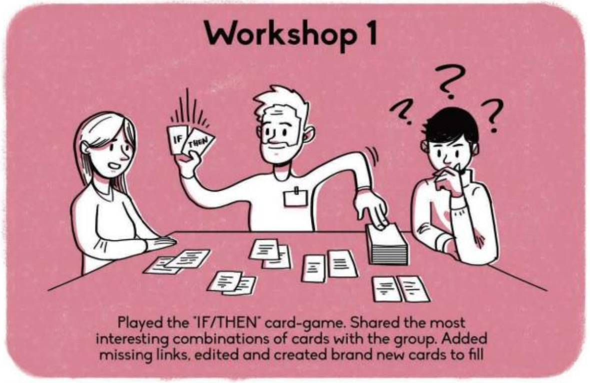

Key Findings:

- Appropriate delivery and accessible language at the first contact is vital.
- Change the cards from 'will' to 'may'. There is no 'one size fits all.'
- Social function can be linked to physical function.

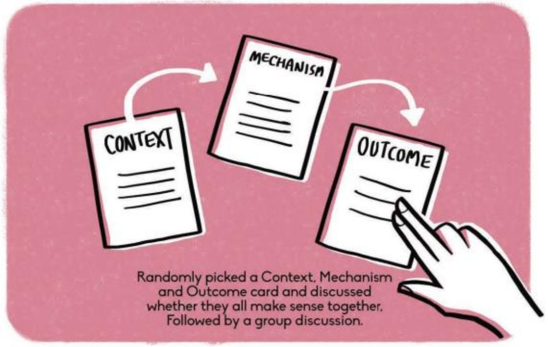

Key Findings:

- Family and personal relationships affect readiness to engage in both positive and negative ways.
- We need to ensure people don't just agree in the GP appointment without taking action afterwards.
- GPs and nurses need to be supported in developing realistic goals shared between both professional and patient.
- Focus on what is possible, rather than focussing on what has been lost.

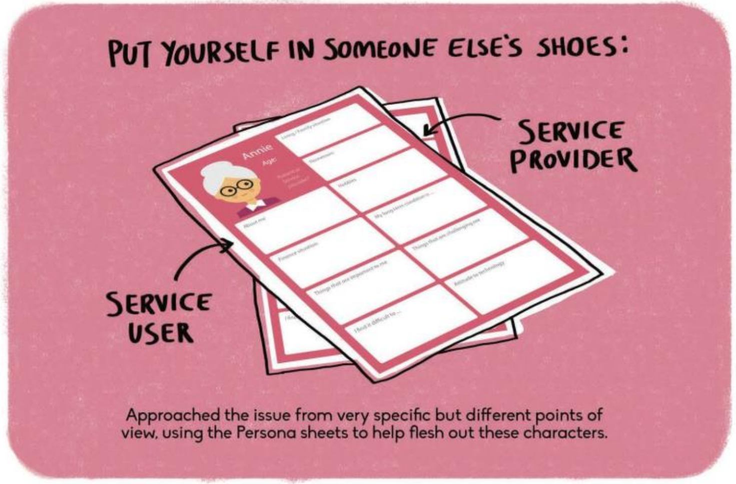

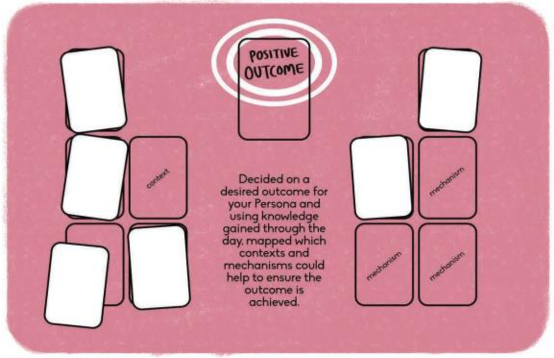

Key Findings:

- People juggle multiple societal roles, and sometimes don't prioritise their own needs.
- Interventions need to take into account personal preferences of the individual and need to be meaningful in a number of different ways for the person to carry on doing it.
- Once there is readiness to change, the need to ask for help is important. Readiness is only the first step in the process.
- Volunteering can be a reciprocal activity. It can address loneliness and provide purpose at the end of your work-life.
- Develop a community/society where anyone can go out (be active) and feel safe.
- What can people offer each other in the community? Do they have access to peer relationships and networks?

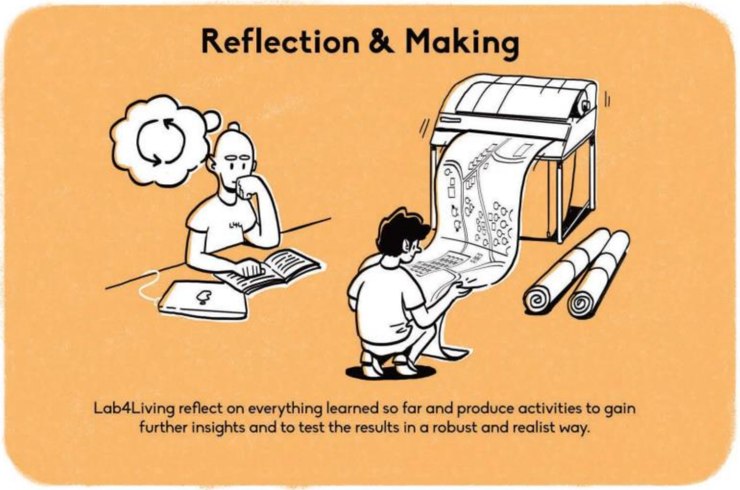

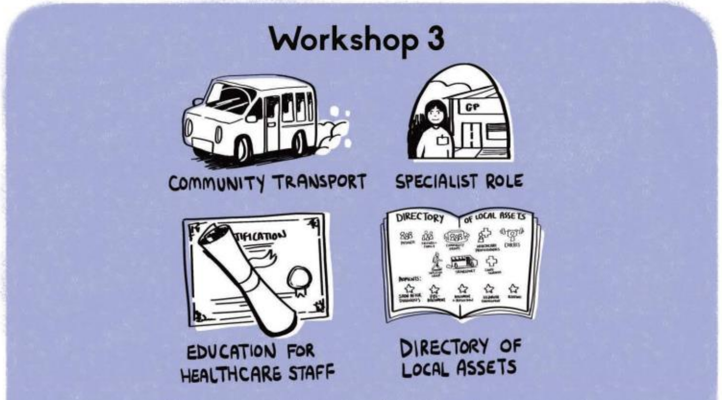

The most commonly mentioned ideas from the previous workshop were reduced into 4 distinct groups.

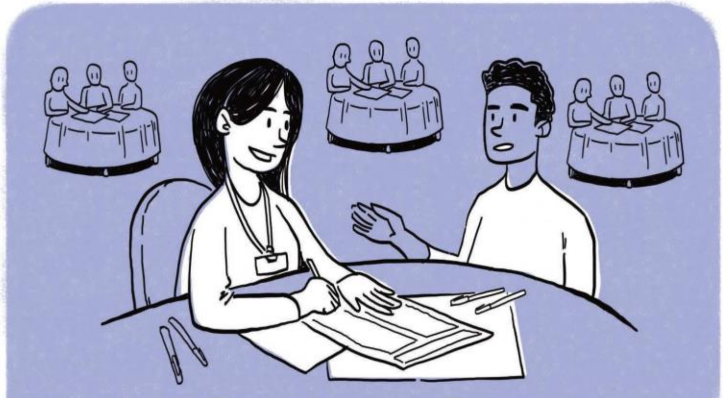

Each group started to flesh out the ideas based on their own specialist knowledge as a patient, community member or healthcare professional.

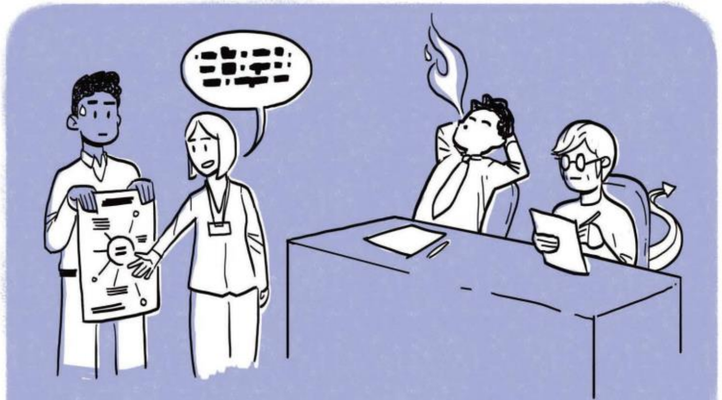

Ideas were presented to a panel of "dragons" made up of GPs, researchers and patient representatives. Ideas were challenged and questioned, exposing further areas of exploration and development.

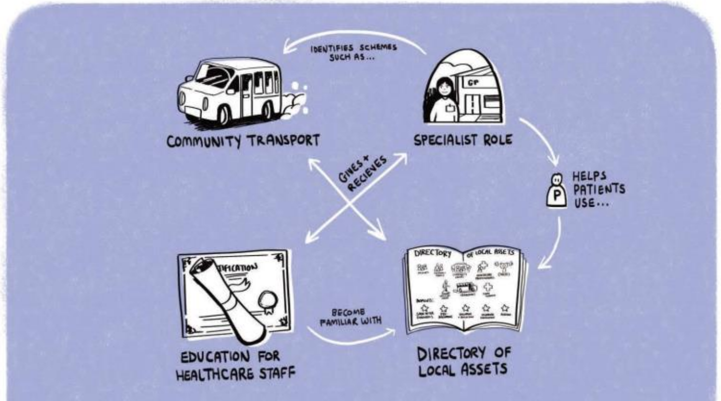

Ideas were further developed based on the dragons recommendations. During the final group discussion the boundaries between ideas began to fall away and connections began to form.

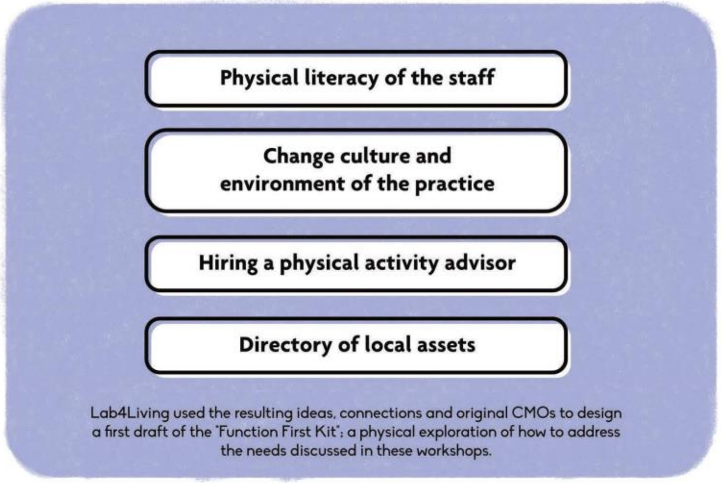
Physical literacy of the staff
Change culture and environment of the practice
Hiring a physical activity advisor
Directory of local assets
Lab4Living used the resulting ideas, connections and original CMOs to design a first draft of the "Function First Kit"; a physical exploration of how to address the needs discussed in these workshops.

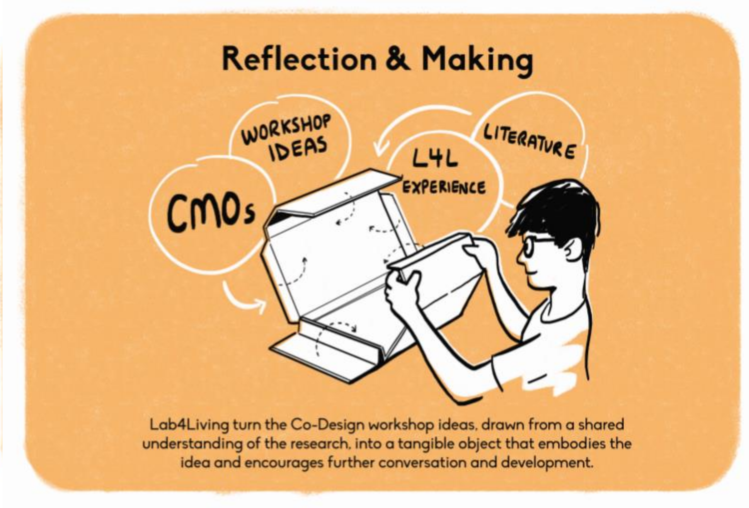
Reflection & Making
WORKSHOP IDEAS
LITERATURE
L4L EXPERIENCE
CMOs
Lab4Living turn the Co-Design workshop ideas, drawn from a shared understanding of the research, into a tangible object that embodies the idea and encourages further conversation and development.

Knowledge Mobilisation Session

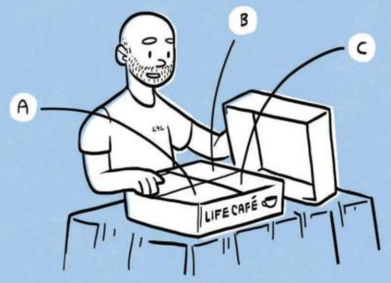

The session started with a walkthrough of previous interventions designed by Lab4Living including Life Cafe to facilitate conversations around end of life and P.E.A.R.L which provides structured reflection which fits into the busy days of healthcare professionals.

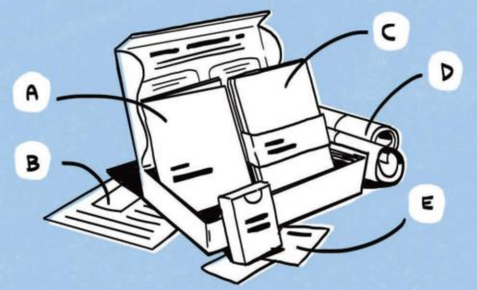

With an understanding of what is possible in terms of physical interventions, Lab4Living presented the Function First box in it's initial form, talking through each part, and how it links back to the co-production which has taken place so far.

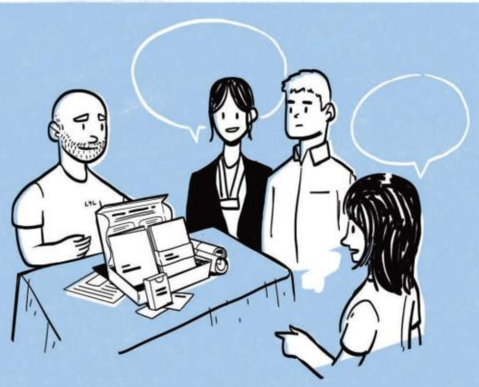

After an introduction to the intervention, a group discussion took place to further analyse, improve and add to the materials designed so far.

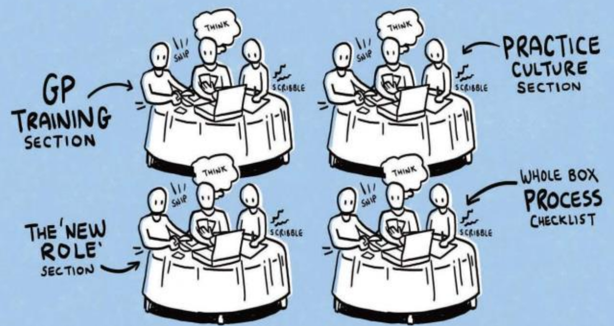

The workshop attendants split into smaller groups and each took a different area of focus. Alongside the designers at Lab4Living they created new content and resources.

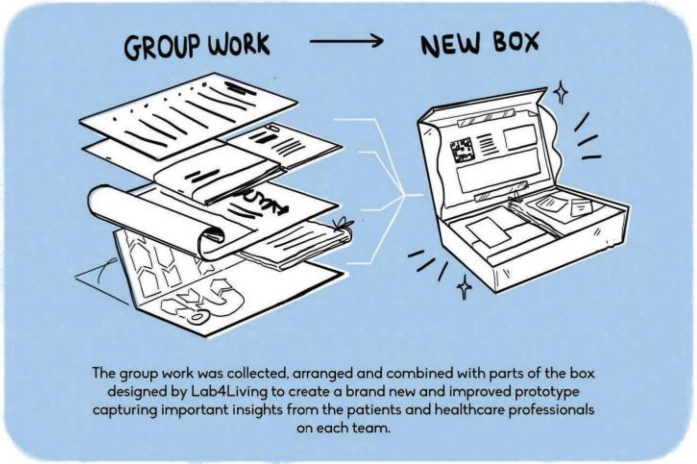
GROUP WORK → NEW BOX
The group work was collected, arranged and combined with parts of the box designed by Lab4Living to create a brand new and improved prototype capturing important insights from the patients and healthcare professionals on each team.

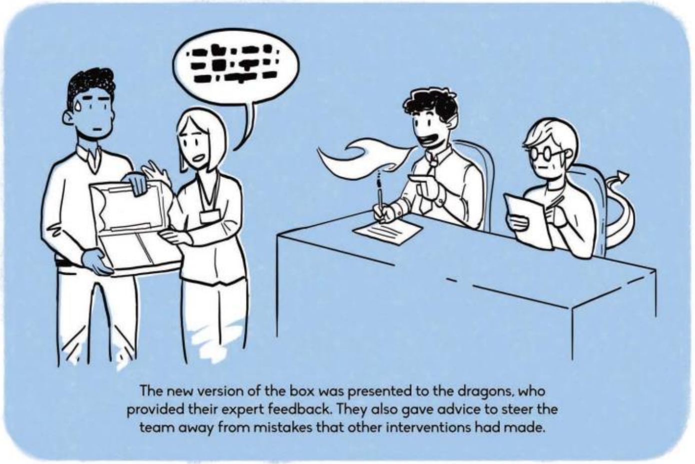
The new version of the box was presented to the dragons, who provided their expert feedback. They also gave advice to steer the team away from mistakes that other interventions had made.

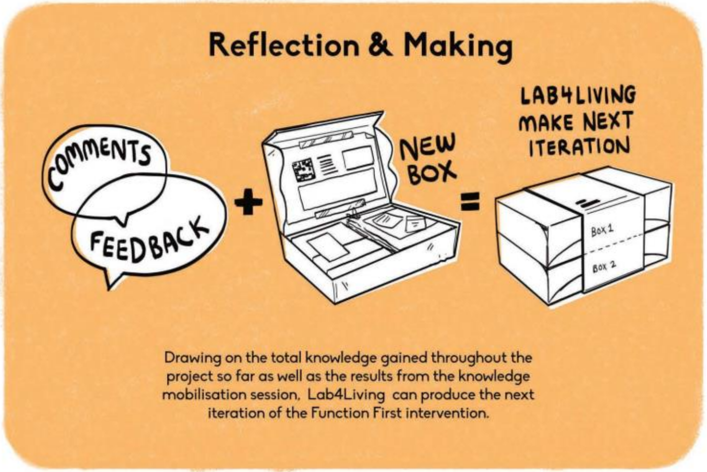
Reflection & Making
COMMENTS
FEEDBACK
+
NEW BOX
=
LAB4LIVING MAKE NEXT ITERATION
Box 1
Box 2
Drawing on the total knowledge gained throughout the project so far as well as the results from the knowledge mobilisation session, Lab4Living can produce the next iteration of the Function First intervention.

For peer review only

Supplementary figure 2: Example models built by participants in the theory-building workshops to reflect on and describe their interpretation of: A) what physical function meant to them, B) how they maintained physical function and C) an example of a 'shared landscape'.

A

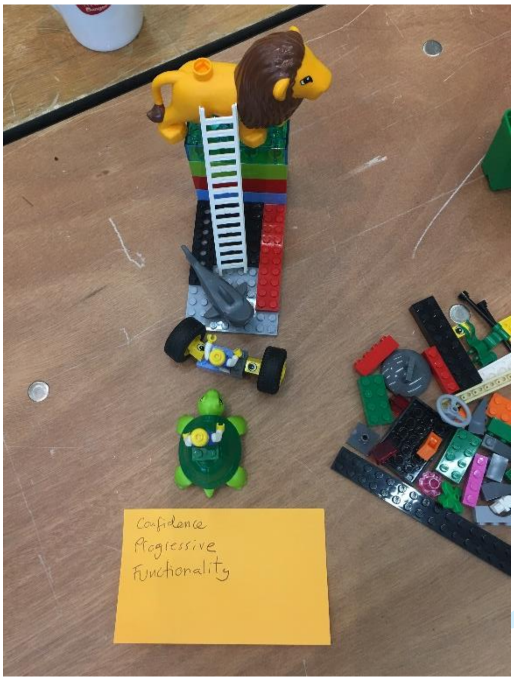

B

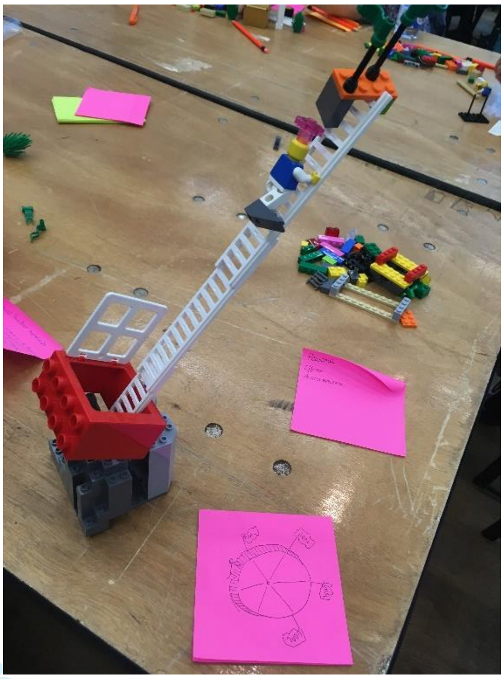

C

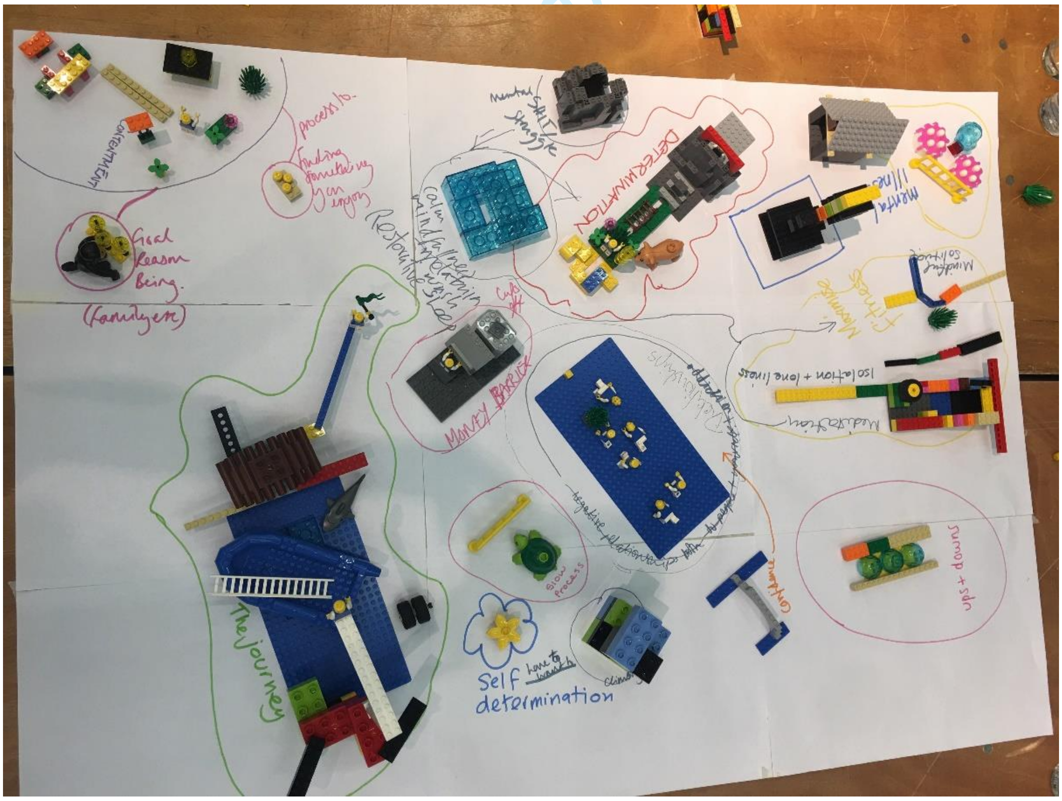

Supplementary table 1: Stakeholder contribution to the theory-building workshops and telephone interviews, and cumulative contribution of individual stakeholders to the co-design and knowledge mobilisation workshops. * = also took part in an individual interview (n = 1 public contributor, n = 1 representative from relevant third sector organisation, who also had a long-term condition, n = 1 GP, n = 1 researcher). ** = also took part in co-design workshops 2 and 3 and the knowledge mobilisation workshop (n = 1). *** = also took part in a co-design workshop 3 and the knowledge mobilisation workshop. # = member of research team. Co-Chief Investigators BL (health services researcher) and NW (academic GP) contributed as stakeholders to all study workshops.

**Theory-building workshops and stakeholder interviews**					**Co-design and knowledge mobilisation**			
**Stakeholder representation**	Theory-building workshop 1 (N = 10)	Theory-building workshop 2 (N = 13)	Telephone interview (N = 10)	**Stakeholder representation**	Co-design workshop 1 (N = 9)	Co-design workshop 2 (N = 14)	Co-design workshop 3 (N = 11)	Knowledge mobilisation workshop (N = 12)
**Public contributor, long-term condition**	n = 5*	n = 6**	n = 3	**Public contributor 1, long-term condition**	✓	✓	✓	✓
**GP**	n = 1	n = 1*	n = 3	**Public contributor 2, long-term condition**	✓	✓	-	✓
**Leisure centre manager**	n = 1	-	-	**Public contributor 3, long-term condition**	✓	-	-	-
**Sport and outdoor recreation division of local council**	n = 1	-	-	**Public contributor 4, long-term condition**	-	✓	✓	✓

**Health and social care public representation group**	n = 1	-	-		**Public contributor 5, long-term condition#**	-	✓	✓	✓
**Practice manager**	-	n = 1	-		**Public contributor 6, long-term condition**	-	✓	-	-
**Researcher, social care**	-	n = 1	-		**Public contributor 7, long-term condition**	-	✓	-	-
**Occupational therapist**	-	n = 1	-		**General Practitioner**	-	✓	✓	✓
**Third sector organisation**	n = 1*	n = 1	-		**Practice nurse**	-	✓	-	-
**Physiotherapist**	-	n = 1	-		**Practice manager**	-	✓	-	-
**Engagement officer**	-	n = 1	-		**Physician associate**	✓	-	✓	✓
**Primary care practice nurse**	-	-	n = 2		**Physiotherapist**	-	✓	-	-
**Primary care healthcare assistant**	-	-	n = 1		**Primary care cluster lead, coordinator of voluntary services**	✓	✓	✓	-
**Researcher, pedagogy**	-	-	n = 1***		**Exercise referral scheme co-ordinator**	✓	✓	✓	-

For peer review only

		**Life coach**	-	✓	-	-
		**Researcher, pedagogy***	-	-	✓	✓
		**Researcher, physical activity promotion**	-	-	-	✓
		**Researcher, clinical exercise physiology#**	-	✓	-	-
		**Researcher, medical sociology#**	-	-	✓	✓
		**Researcher, health psychology#**	✓	-	✓	✓
		**Researcher, nursing, rehabilitation and implementation#**	✓	-	-	-
		**Researcher, sports physiology#**	✓	-	✓	✓
		**Researcher, information science#**	-	-	-	✓
					**5 ‘Dragon’s’/independent advisors included representation from:**	**4 ‘Dragon’s’/independent advisors included representation from**

peer review only

					• **2 public contributors with long-term conditions**<br>• **General practice**<br>• **Public Health**<br>• **Physical activity promotion research**	**organisations and professional bodies relevant to:**<br>• **General practice**<br>• **Public health**<br>• **Nursing**<br>• **Physiotherapy**

For peer review only

Supplementary table 2: If…Then statements

**Overarching theory areas**
**Physical literacy:** the motivation, confidence, physical competence, knowledge and understanding to value and take responsibility for engagement in physical activity for life (Jones et al, 2018)
**International Classification of Functioning:** The International Classification of Functioning, Disability and Health, known more commonly as ICF, is the World Health Organisation's framework for measuring health and disability at both individual and population levels (World Health Organisation, 2019).
**If…then statements**
**Level: Local engagement outside of practice**
If people have access to a variety of physical activity opportunities, then they will be more likely to pursue opportunities to be more physically active.
If there are sufficient places on exercise referral schemes and physiotherapy clinics, then people will receive timely interventions to improve physical function
**Level: Whole practice**
If there is a culture of physical literacy in the practice then patients are more likely to receive interventions that promote physical activity
If a range of referral options are available according to patients' functional status (pyramid model) then more patients will receive appropriate interventions to enhance their physical function
If there are sufficient staff working in the practice who are trained to promote physical literacy then more patients will receive timely physical activity interventions
If staff are trained in a variety of techniques to encourage physical activity (goal-setting, coaching, motivational interviewing) then they are more likely to promote physical literacy
If the practice has good links with outside agencies/social networks providing a wide range of physical activities in the local area then more people will engage with physical activities
If there are relevant features and cues within the built environment, then physical activity and physical function will improve.
**Level: Health professional consultations with people with long-term conditions**
If physical literacy is promoted during routine primary care management of long-term conditions, then physical activity will increase and physical functioning will improve.
If consultations with people with long-term conditions consistently address physical activity and physical function, then people will be more likely to prioritise this element of management
If physical literacy is promoted in each long-term condition consultation then physical literacy will be integrated into routine care

For peer review only

If self-efficacy and goal-setting are promoted as part of routine care then physical activity interventions will be more meaningful to patients and uptake and adherence will increase.
If pacing is used in physical activity promotion, incorporating periods of rest and recuperation, then patients with low energy levels and low fitness levels are more likely to persist with physical activity interventions
**Level: Individual patients**
Physical
If physical activity advice is tailored to patients' own priorities for functioning, then they are more likely to start and continue physical activities
If a functional approach is adoped to the management of long-term conditions, then interventions will be more relevant and tailored towards functional limitations that are meaningful to the individual
Psychological
If being physically active is linked to the freedom, well-being and joy associated with having physical function, then people with long-term conditions will better identify with the reasoning for being physically active and be more likely to continue.
If enjoyment is emphasised as an important element of physical activity, then people will be more motivated to pursue physical activity opportunities and therefore maintain physical function.
If people have built an engrained physical activity 'identity' through previous experiences, then they will be more likely to be motivated to pursue physical activity opportunities
If people with long-term conditions are supported to develop contingency plans for unpredictable circumstances, then they will be more likely to be able to persist with physical activity.
If people have self-efficacy for maintaining and improving physical activity and physical function, then they will be more likely to lead a physically active lifestyle.
If anxiety about exercising is reduced (e.g. fatigue, soreness, shortness of breath, tachycardia) through educational interventions about the normal physiological effect of exercise, then people are more likely to start and continue being physically active.
If people have recently been diagnosed with a long-term condition or experienced a life event, then a window of opportunity where people are most receptive can be utilised to promote physical activity in order to maintain and improve physical function.
If low mood reduces the motivation to exercise then improving mood will increase physical activity participation
If inspiration (indirect and direct) is provded for people with long-term conditions, then this will facilitate the development of self-efficacy for improving physical activity and physical function
If people develop a sense of agency relating to physical activity and physical function, then they will be more likely to pursue physical activity opportunities.
Social

For peer review only

If people feel supported by family or friends then they are more likely to start and continue physical activity
If people perform physical activity or exercise in groups then they are more likely to continue
If people with long-term conditions are labelled/stereotyped less, then perceived and actual restrictions will reduce, encouraging improvements in physical activity and physical function

For peer review only

Supplementary table 3: Initial inclusion/exclusion criteria

**Screening tool: Function First**	
**<u>Primary research question:</u>**<br>**What is the role of primary care in reducing the decline in physical function and physical activity for people with long-term conditions: what works, for whom and in what circumstances?**<br>**<u>Secondary research objectives:</u>**<br>**a)** To identify and produce a taxonomy of physical activity interventions that aim to reduce functional decline in people with long-term conditions managed in primary care.<br>**b)** To uncover the complexity associated with the range of physical activity interventions in primary care, and how they directly or indirectly affect the physical functioning of people with long-term conditions.<br>**c)** To identify the mechanisms through which interventions bring about functional improvements in people with long-term conditions, and the circumstances associated with how the interventions are organised and operate within different primary care contexts.<br>**d)** To understand the potential impacts of these interventions across primary care and other settings, such as secondary healthcare and social care, paying attention to the conditions which influence how they operate.	
**Questions – key elements**	
**Population (P) and Conditions**	**Include:**<br>**People with long-term conditions** or **people who are likely to have a long-term condition** (e.g. frail or 'pre-frail/at risk of frailty', reduced mobility, living in care home).<br>'Long-term conditions or chronic diseases are conditions for which there is currently no cure, and which are managed with drugs and other treatment, for example: diabetes, chronic obstructive pulmonary disease, arthritis, hypertension' (DOH/Kings Fund) – including cancer, addiction/substance abuse, mental health conditions, heart conditions, chronic pain, stroke, obesity, learning disabilities, multiple sclerosis.<br>**<u>OR</u>**<br>**People promoting physical activity to people with long-term conditions**<br>GPs, nurse practitioners, practice nurses, physician associates, physio/occupational therapists, multi-disciplinary teams, other primary care staff, exercise physiologists, exercise professionals, exercise instructors, coaches, people providing exercise opportunities in the community, professionals providing rehabilitation interventions (eg cardiac or pulmonary rehabilitation).
	**Exclude:** Healthy but sedentary people; injured/acutely unwell people (e.g. injury, mechanical problems, fracture or sepsis); pre-conditions (e.g. pre-diabetes, osteopenia); pregnancy; menopause; children and adolescents aged <18 years; animal studies.
**Interventions (I)**	**Include: <u>Physical activity interventions</u>** designed to improve physical function and physical activity.
	**Exclude:** Physiotherapy, physical therapy, mechanical/manual therapy without any physical activity/exercise intervention. Interventions with limited transferability to NHS primary care, pharmacological agents, technical, high-cost equipment, short-term rehabilitation following injury, fracture, sepsis, post-operative; breathing exercises.
**Outcomes (O)**	**Include:** Any outcomes targeting improving physical activity or physical function (including psychological and social outcomes).
	**Exclude:** Physiological measurements (e.g. lung function, cardiovascular function, physiology laboratory), or biochemical markers (e.g. blood measures of disease activity/severity) or therapy-specific (e.g. motor control, range of motion/biomechanical, cognitive outcomes only).
**Settings (S)**	**Include:** Studies in primary care, general medical practice, community settings, care homes, intermediate care including reablement (recovering from hospital stay), community or outpatient cardiac or pulmonary rehabilitation).
	**Exclude:** Physiology laboratory, hospital in-patient (e.g. exercise during dialysis or chemotherapy).
**Study type**	**Include:** Any study design, including reviews, qualitative papers about barriers and facilitators and especially theory-rich papers with potential mechanisms, process evaluations, etc.
	**Exclude:** N/A (to screen later for theoretical richness)

For peer review only - http://bmjopen.bmj.com/site/about/guidelines.xhtml

Supplementary table 4: List of included papers

Author and year	Title
Allen 2012[1]	Patient and provider interventions for managing osteoarthritis in primary care: protocols for two randomized controlled trials
Allen 2017 [2]	Patient, provider, and combined interventions for managing osteoarthritis in primary care: A cluster randomized trial
Andryukhin 2010 [3]	The impact of a nurse-led care programme on events and physical and psychosocial parameters in patients with heart failure with preserved ejection fraction: A randomized clinical trial in primary care in Russia
Arden 2017 [4]	Evaluation of a rolling rehabilitation programme for patients with non-specific low back pain in primary care: an observational cohort study
Åsenlöf 2005 [5]	Individually tailored treatment targeting activity, motor behavior, and cognition reduces pain-related disability: A randomized controlled trial in patients with musculoskeletal pain
Åsenlöf 2009 [6]	Long-term follow-up of tailored behavioural treatment and exercise based physical therapy in persistent musculoskeletal pain: A randomized controlled trial in primary care
Avery 2016 [7]	Systematic development of a theory-informed multifaceted behavioural intervention to increase physical activity of adults with type 2 diabetes in routine primary care: Movement as Medicine for Type 2 Diabetes
Barrett 2017 [8]	Feasibility of a physical activity pathway for Irish primary care physiotherapy services
Bearne 2011 [9]	Feasibility of an exercise-based rehabilitation programme for chronic hip pain
Bierman 2001 [10]	Functional status, the sixth vital sign
Bickerdike 2017 [11]	Social prescribing: less rhetoric and more reality. A systematic review of the evidence
Bird 2019 [12]	General practice referral of 'at risk'populations to community leisure services: applying the RE-AIM framework to evaluate the impact of a community-based physical activity programme for inactive adults with long-term conditions
Bjerk 2017 [13]	A falls prevention programme to improve quality of life, physical function and falls efficacy in older people receiving home help services: Study protocol for a randomised controlled trial
Bjerre 2019 [14]	Community-based football in men with prostate cancer: 1-year follow-up on a pragmatic, multicentre randomised controlled trial
Boehler 2011 [15]	The cost of changing physical activity behaviour: evidence from a" physical activity pathway" in the primary care setting
Bossen 2013 [16]	Effectiveness of a web-based physical activity intervention in patients with knee and/or hip osteoarthritis: randomized controlled trial
Brannan 2019 [17]	Moving healthcare professionals–a whole system approach to embed physical activity in clinical practice
Bull 1995 [18]	Beliefs and behaviour of general practitioners regarding promotion of physical activity

For peer review only

Bull 2008 [19]	Evaluation of the Physical Activity Care Pathway London Feasibility Pilot– Final Technical Report
Bull and Milton 2010 [20]	A process evaluation of a" physical activity pathway" in the primary care setting.
Campbell 2015 [21]	A systematic review and economic evaluation of exercise referral schemes in primary care: a short report
Chaplin 2015 [22]	The evaluation of an interactive web-based Pulmonary Rehabilitation programme: protocol for the WEB SPACE for COPD feasibility study
Chatterjee 2017 [23]	GPs' knowledge, use, and confidence in national physical activity and health guidelines and tools: a questionnaire-based survey of general practice in England
Chong 2014 [24]	Physical activity program preferences and perspectives of older adults with and without cognitive impairment
Comer 2013 [25]	A Home Exercise Programme Is No More Beneficial than Advice and Education for People with Neurogenic Claudication: Results from a Randomised Controlled Trial
Coombes 2015 [26]	"Exercise is medicine": Curbing the burden of chronic disease and physical inactivity
Copeland 2019 [27]	Evaluation of the Public Health England and Sport England Funded Physical Activity Clinical Advice Pad Pilot
Coulter 2016 [28]	Personalised care planning for adults with chronic or long-term health conditions
Craike 2019 [29]	General practitioner referrals to exercise physiologists during routine practice: A prospective study
Croteau 2006 [30]	Physical activity advice in the primary care setting: results of a population study in New Zealand.
Dacey 2014 [31]	Physical activity counseling in medical school education: a systematic review
Daniellson 2016 [32]	Crawling Out of the Cocoon: Patients' Experiences of a Physical Therapy Exercise Intervention in the Treatment of Major Depression.
Dejonghe 2020 [33]	Health coaching for promoting physical activity in low back pain patients: a secondary analysis on the usage and acceptance
Devi 2014 [34]	A web-based program improves physical activity outcomes in a primary care angina population: Randomized controlled trial
Din 2015 [35]	Health professionals' perspectives on exercise referral from a process evaluation of the National Exercise Referral Scheme in Wales
Dunlop and Murray 2013 [36]	Major limitations in knowledge of physical activity guidelines among UK medical students revealed: implications for the undergraduate medical curriculum
Eakin 2008 [37]	The Logan Healthy Living Program: A cluster randomized trial of a telephone-delivered physical activity and dietary behavior intervention for primary care patients with type 2 diabetes or hypertension from a socially disadvantaged community - Rationale, design and recruitment
Eakin 2010a [38]	Living Well with Diabetes: a randomized controlled trial of a telephone-delivered intervention for maintenance of weight loss, physical activity and glycaemic control in adults with type 2 diabetes.

For peer review only

Eakin 2010b [39]	Maintenance of physical activity and dietary change following a telephone-delivered intervention
Ewald 2018 [40]	Physical activity coaching by Australian Exercise Physiologists is cost effective for patients referred from general practice
Fife-Schaw 2014 [41]	Comparing exercise interventions to increase persistence with physical exercise and sporting activity among people with hypertension or high normal blood pressure: Study protocol for a randomised controlled trial
Forsyth 2009 [42]	Dietitians and exercise physiologists in primary care: Lifestyle interventions for patients with depression and/or anxiety
Gamboa Moreno 2013 [43]	Impact of a self-care education programme on patients with type 2 diabetes in primary care in the Basque Country
Gamboa Moreno 2016 [44]	A Pilot Study to Assess the Feasibility of the Spanish Diabetes Self-Management Program in the Basque Country
Goode 2012 [45]	Telephone-delivered interventions for physical activity and dietary behavior change: an updated systematic review
Grant 2014 [46]	Exercise as a vital sign: A quasi-experimental analysis of a health system intervention to collect patient-reported exercise levels
Healey 2018 [47]	The feasibility and acceptability of a physical activity intervention for older people with chronic musculoskeletal pain: The iPOPP pilot trial protocol
Hinrichs 2011a [48]	General practitioner advice on physical activity: analyses in a cohort of older primary health care patients (getABI)
Hinrichs 2011b [49]	Effects of an exercise programme for chronically ill and mobility-restricted elderly with structured support by the general practitioner's practice (HOMEfit) - study protocol of a randomised controlled trial
Hinrichs 2016 [50]	Home-Based Exercise Supported by General Practitioner Practices: Ineffective in a Sample of Chronically Ill, Mobility-Limited Older Adults (the HOMEfit Randomized Controlled Trial)
Holden 2012 [51]	Role of exercise for knee pain: What do older adults in the community think?
Hurley 2018 [52]	Exercise interventions and patient beliefs for people with hip, knee or hip and knee osteoarthritis: A mixed methods review
Husk 2019 [53]	What approaches to social prescribing work, for whom, and in what circumstances? A realist review
James 2017 [54]	Referral for Expert Physical Activity Counseling: A Pragmatic RCT
Jansink 2010 [55]	Primary care nurses struggle with lifestyle counseling in diabetes care: a qualitative analysis
Jones 2018 [56]	Development of a physical literacy model for older adults–a consensus process by the collaborative working group on physical literacy for older Canadians
Jorgensen 2012 [57]	How do general practitioners in Denmark promote physical activity?
Kosteli 2017 [58]	Barriers and enablers of physical activity engagement for patients with COPD in primary care
Lamming 2017 [59]	What do we know about brief interventions for physical activity that could be delivered in primary care consultations? A systematic review of reviews

For peer review only

Leemrijse 2015 [60]	Collaboration of general practitioners and exercise providers in promotion of physical activity a written survey among general practitioners
Leenaars 2016 [61]	The role of the care sport connector in the Netherlands
Leijon 2008 [62]	Physical activity referrals in Swedish primary health care - Prescriber and patient characteristics, reasons for prescriptions, and prescribed activities
Lindeman 2020 [63]	The extent to which family physicians record their patients' exercise in medical records: a scoping review
Lion 2019 [64]	Physical activity promotion in primary care: a Utopian quest?
Lobelo 2009 [65]	Physical activity habits of doctors and medical students influence their counselling practices
Lohmann 2010 [66]	Fitness consultations in routine care of patients with type 2 diabetes in general practice: An 18-month non-randomised intervention study
Loughren 2014 [67]	'Let's Get Moving'Physical Activity Care Pathway (Gloucestershire) Post-Programme Evaluation Report.
Martin-Borras 2018 [68]	A new model of exercise referral scheme in primary care: is the effect on adherence to physical activity sustainable in the long term? A 15-month randomised controlled trial
McDonough 2013 [69]	Pedometer-driven walking for chronic low back pain: A feasibility randomized controlled trial
McKay 2001 [70]	The Diabetes Network Internet-Based Physical Activity Intervention A randomized pilot study.
Melillio 2000 [71]	Perceptions of nurse practitioners regarding their role in physical activity and exercise prescription for older adults
Moore 2013 [72]	Mixed-method process evaluation of the welsh national exercise referral scheme
Morgan 2015 [73]	Physical ACtivity facilitation for Elders (PACE): Study protocol for a randomised controlled trial
Morishita 2014 [74]	Primary care physicians' own exercise habits influence exercise counseling for patients with chronic kidney disease: A cross-sectional study
Faculty of Sport and Exercise Medicine UK 2018 [75]	'Moving Medicine'
Muellmann 2018 [76]	Effectiveness of eHealth interventions for the promotion of physical activity in older adults: A systematic review
Murphy 2012 [77]	An exploratory cluster randomised trial of a university halls of residence based social norms intervention in Wales, UK
NHS leading change [78]	Introducing group consultations for adults with Type 2 diabetes
NICE 2015 [79]	Dementia, disability and frailty in later life – mid-life approaches to delay or prevent onset.
NICE 2013 [80]	Physical activity: brief advice for adults in primary care
NICE 2014 [81]	Behaviour change: individual approaches
NICE 2014 [82]	Physical activity: exercise referral schemes.
NICE 2019 [83]	Making Every Contact Count: How NICE resources can support local priorities

For peer review only

Omura 2018 [84]	Primary care providers' physical activity counseling and referral practices and barriers for cardiovascular disease prevention
Parish 2006 [85]	Examination of the constructs of the Transtheoretical model in patients with heart failure: a focus on physical activity readiness.
Parkrun practice [86]	parkrun UK teams up with RCGP to 'prescribe' active lifestyles to patients and practice staff
Persson 2013 [87]	Physical activity on prescription (PAP) from the general practitioner's perspective–a qualitative study
Pescheny 2018 [88]	Facilitators and barriers of implementing and delivering social prescribing services: a systematic review
Prochaska 2000 [89]	PACE Interactive Communication Technology for Behavior Change in Clinical Settings
Department of Health 2012 [90]	Let's Get Moving commissioning guidance
Quirk and Haarke 2019 [91]	How can we get more people with long-term health conditions involved in parkrun? A qualitative study evaluating parkrun's PROVE project
Rhodes 2020 [92]	Increasing physical activity by four legs rather than two: systematic review of dog-facilitated physical activity interventions
Royal College of General Practitioners [93]	Active Practice' Website
Rushforth 2016 [94]	Barriers to effective management of type 2 diabetes in primary care: qualitative systematic review
Savill 2015 [95]	Is general practice engaged with physical activity promotion?
Schofield 2005 [96]	Trust levels of physical activity information sources: a population study
Shaw 2012 [97]	Exercise for overweight or obesity
Short 2016 [98]	Physical activity recommendations from general practitioners in Australia. Results from a national survey
Smith 2016 [99]	Interventions for improving outcomes in patients with multimorbidity in primary care and community settings
Smith 2019 [100]	Social prescribing programmes to prevent or delay frailty in community-dwelling older adults
Stone 2015 [101]	Painful choices: a qualitative exploration of facilitators and barriers to active lifestyles among adults with osteoarthritis
Sturgiss 2016 [102]	Increasing general practitioners' confidence and self-efficacy in managing obesity: a mixed methods study
UK Chief Medical Officers 2011 [103]	Start Active, Stay Active: A report on physical activity from the four home countries' Chief Medical Officers (now updated, see below)
UK Chief Medical Officers 2019 [104]	Physical activity guidelines: UK Chief Medical Officers' report
Physical Activity Guidelines for Americans [105]	Physical Activity Guidelines for Americans
Van der Wulp 2012 [106]	Effectiveness of peer-led self-management coaching for patients recently diagnosed with Type 2 diabetes mellitus in primary care: A randomized controlled trial

For peer review only

Val Slujis 2005 [107]	Effect of a tailored physical activity intervention delivered in general practice settings: results of a randomized controlled trial
Van Slujis 2005 [108]	The positive effect on determinants of physical activity of a tailored, general practice-based physical activity intervention
Vanroy 2017 [109]	Short- and long-term effects of a need-supportive physical activity intervention among patients with type 2 diabetes mellitus: A randomized controlled pilot trial
Verwey 2014 [110]	A pilot study of a tool to stimulate physical activity in patients with COPD or type 2 diabetes in primary care
Verwey 2014 [111]	A monitoring and feedback tool embedded in a counselling protocol to increase physical activity of patients with COPD or type 2 diabetes in primary care: Study protocol of a three-arm cluster randomised controlled trial
Verwey 2016 [112]	Upgrading physical activity counselling in primary care in the Netherlands, Oxford University Press.
Verwey 2016 [113]	Process evaluation of physical activity counselling with and without the use of mobile technology: A mixed methods study
Walsh 1999 [114]	Exercise Counseling by Primary Care Physicians in the Era of Managed Care.
Ward 2015 [115]	A Survey of Physical Activity in MedicalCurricula: A report of the HEPA in Health Care SettingsHEPA Europe Working Group
Weiler 2012 [116]	Physical activity education in the undergraduate curricula of all UK medical schools. Are tomorrow's doctors equipped to follow clinical guidelines?
Weinstock 2011 [117]	Lessened decline in physical activity and impairment of older adults with diabetes with telemedicine and pedometer use: results from the IDEATel study." Age and Ageing 40(1): 98-105.
Wheeler 2019 [118]	Primary care knowledge and beliefs about physical activity and health: a survey of primary healthcare team members
Wilcox 2010 [119]	Adoption and Implementation of Physical Activity and Dietary Counseling by Community Health Center Providers and Nurses.
Williams 2020 [120]	Translating a walking intervention for health professional delivery within primary care: A mixed-methods treatment fidelity assessment.
Wormald and Ingle 2004 [121]	Hull and East Riding Primary Care Trusts, Hull. 2 Lecturer in Exercise Physiology.

Full reference list

1. Allen, K.D., et al., *Patient, provider, and combined interventions for managing osteoarthritis in primary care: A cluster randomized trial.* Annals of Internal Medicine, 2017. **166**(6): p. 401-411.
2. Allen, K.D., et al., *Patient and provider interventions for managing osteoarthritis in primary care: protocols for two randomized controlled trials*, 2012.
3. Andryukhin, A., et al., *The impact of a nurse-led care programme on events and physical and psychosocial parameters in patients with heart failure with preserved ejection fraction: A randomized clinical trial in primary care in Russia.* European Journal of General Practice, 2010. **16**(4): p. 205-214.

For peer review only

4. Arden, K., F. Fatoye, and G. Yeowell, *Evaluation of a rolling rehabilitation programme for patients with non-specific low back pain in primary care: an observational cohort study.* Journal of Evaluation in Clinical Practice, 2017. **23**(2): p. 272-278.
5. Åsenlöf, P., E. Denison, and P. Lindberg, *Individually tailored treatment targeting activity, motor behavior, and cognition reduces pain-related disability: A randomized controlled trial in patients with musculoskeletal pain.* Journal of Pain, 2005. **6**(9): p. 588-603.
6. Åsenlöf, P., E. Denison, and P. Lindberg, *Long-term follow-up of tailored behavioural treatment and exercise based physical therapy in persistent musculoskeletal pain: A randomized controlled trial in primary care.* European Journal of Pain, 2009. **13**(10): p. 1080-1088.
7. Avery, L., et al., *Systematic development of a theory-informed multifaceted behavioural intervention to increase physical activity of adults with type 2 diabetes in routine primary care: Movement as Medicine for Type 2 Diabetes.* Implement Sci, 2016. **11**(1).
8. Barrett, E.M., J. Hussey, and C.D. Darker, *Feasibility of a physical activity pathway for Irish primary care physiotherapy services.* Physiotherapy, 2017. **103**(1): p. 106-112.
9. Bearne, L.M., et al., *Feasibility of an exercise-based rehabilitation programme for chronic hip pain.* Musculoskeletal Care, 2011. **9**(3): p. 160-168.
10. Bierman, A.S., *Functional status, the sixth vital sign.* Journal of General Internal Medicine, 2001(16): p. 785-786.
11. Bickerdike, L., et al., *Social prescribing: less rhetoric and more reality. A systematic review of the evidence.* BMJ open, 2017. **7**(4): p. e013384.
12. Bird, E.L., M.S.Y. Biddle, and J.E. Powell, *General practice referral of 'at risk'populations to community leisure services: applying the RE-AIM framework to evaluate the impact of a community-based physical activity programme for inactive adults with long-term conditions.* BMC public health, 2019. **19**(1): p. 1308.
13. Bjerk, M., et al., *A falls prevention programme to improve quality of life, physical function and falls efficacy in older people receiving home help services: Study protocol for a randomised controlled trial.* BMC Health Services Research, 2017. **17**(1).
14. Bjerre, E.D., et al., *Community-based football in men with prostate cancer: 1-year follow-up on a pragmatic, multicentre randomised controlled trial.* PLoS medicine, 2019. **16**(10).
15. Boehler, C., et al., *The cost of changing physical activity behaviour: evidence from a "physical activity pathway" in the primary care setting.* BMC Public Health, 2011. **11**(1): p. 370-370.
16. Bossen, D., et al., *Effectiveness of a web-based physical activity intervention in patients with knee and/or hip osteoarthritis: randomized controlled trial.* Journal of medical Internet research, 2013. **15**(11).
17. Brannan, M., et al., *Moving healthcare professionals–a whole system approach to embed physical activity in clinical practice.* BMC Med Educ, 2019. **19**(1): p. 84.
18. Bull, F.C.L., et al., *Beliefs and behaviour of general practitioners regarding promotion of physical activity.* Australian Journal of Public Health, 1995. **19**(3): p. 300-304.
19. Bull, F., K. Milton, and C. Boehler, *Evaluation of the Physical Activity Care Pathway London Feasibility Pilot–Final Technical Report*, 2008: https://assets.publishing.service.gov.uk/government/uploads/system/uploads/attachment_data/file/192041/Evaluation_of_the_Physical_Activity_Care_Pathway_London_Feasibility_Pilot_-_Report.pdf.
20. Bull, F.C. and K.E. Milton, *A process evaluation of a" physical activity pathway" in the primary care setting.* BMC Public Health, 2010. **10**(1): p. 463.
21. Campbell, F., et al., *A systematic review and economic evaluation of exercise referral schemes in primary care: a short report.* Health Technol Assess, 2015. **19**(60).
22. Chaplin, E., et al., *The evaluation of an interactive web-based Pulmonary Rehabilitation programme: protocol for the WEB SPACE for COPD feasibility study.* BMJ open, 2015. **5**(8): p. e008055.

23. Chatterjee, R., et al., *GPs' knowledge, use, and confidence in national physical activity and health guidelines and tools: a questionnaire-based survey of general practice in England.* Br J Gen Pract, 2017. **67**(663): p. e668-e675.
24. Chong, T.W.H., et al., *Physical activity program preferences and perspectives of older adults with and without cognitive impairment.* Asia-Pacific Psychiatry, 2014. **6**(2): p. 179-190.
25. Comer, C., et al., *A Home Exercise Programme Is No More Beneficial than Advice and Education for People with Neurogenic Claudication: Results from a Randomised Controlled Trial.* PLoS ONE, 2013. **8**(9).
26. Coombes, J.S., et al., *"Exercise is medicine": Curbing the burden of chronic disease and physical inactivity.* Asia-Pacific Journal of Public Health, 2015. **27**(2): p. NP600-NP605.
27. Copeland, R., et al., *Evaluation of the Public Health England and Sport England Funded Physical Activity Clinical Advice Pad Pilot*, 2019: Sheffield: National Centre for Sport and Exercise Medicine.
28. Coulter, A., et al., *Personalised care planning for adults with chronic or long-term health conditions.* Cochrane Database Syst Rev, 2015. **3**(3).
29. Craike, M., et al., *General practitioner referrals to exercise physiologists during routine practice: A prospective study.* Journal of science and medicine in sport, 2019. **22**(4): p. 478-483.
30. Croteau, K., G. Schofield, and G. McLean, *Physical activity advice in the primary care setting: results of a population study in New Zealand.* Australian and New Zealand journal of public health, 2006. **30**(3): p. 262-267.
31. Dacey, M.L., et al., *Physical activity counseling in medical school education: a systematic review.* Medical education online, 2014. **19**(1): p. 24325.
32. Danielsson, L., B. Kihlbom, and S. Rosberg, *"Crawling Out of the Cocoon": Patients' Experiences of a Physical Therapy Exercise Intervention in the Treatment of Major Depression*, 2016.
33. Dejonghe, L.A.L., et al., *Health coaching for promoting physical activity in low back pain patients: a secondary analysis on the usage and acceptance.* BMC Sports Science, Medicine and Rehabilitation, 2020. **12**(1): p. 2.
34. Devi, R., J. Powell, and S. Singh, *A web-based program improves physical activity outcomes in a primary care angina population: Randomized controlled trial.* Journal of Medical Internet Research, 2014. **16**(9).
35. Din, N.U., et al., *Health professionals' perspectives on exercise referral from a process evaluation of the National Exercise Referral Scheme in Wales.* Health Education J 2015. **74**(6): p. 743-757.
36. Dunlop, M. and A.D. Murray, *Major limitations in knowledge of physical activity guidelines among UK medical students revealed: implications for the undergraduate medical curriculum.* Br J Sports Med, 2013. **47**(11): p. 718-720.
37. Eakin, E.G., et al., *The Logan Healthy Living Program: A cluster randomized trial of a telephone-delivered physical activity and dietary behavior intervention for primary care patients with type 2 diabetes or hypertension from a socially disadvantaged community - Rationale, design and recruitment.* Contemporary Clinical Trials, 2008. **29**(3): p. 439-454.
38. Eakin, E.G., et al., *Living Well with Diabetes: a randomized controlled trial of a telephone-delivered intervention for maintenance of weight loss, physical activity and glycaemic control in adults with type 2 diabetes*, 2010.
39. Eakin, E., et al., *Maintenance of physical activity and dietary change following a telephone-delivered intervention.* Health Psychology, 2010. **29**(6): p. 566-573.
40. Ewald, B., et al., *Physical activity coaching by Australian Exercise Physiologists is cost effective for patients referred from general practice.* Aust N Z J Public Health, 2018. **42**(1): p. 12-15.

For peer review only

41. Fife-Schaw, C., et al., *Comparing exercise interventions to increase persistence with physical exercise and sporting activity among people with hypertension or high normal blood pressure: Study protocol for a randomised controlled trial.* Trials, 2014. **15**(1).
42. Forsyth, A., F.P. Deane, and P. Williams, *Dietitians and exercise physiologists in primary care: Lifestyle interventions for patients with depression and/or anxiety.* Journal of Allied Health, 2009.
43. Gamboa Moreno, E., et al., *Impact of a self-care education programme on patients with type 2 diabetes in primary care in the Basque Country.* BMC Public Health, 2013. **13**(1).
44. Gamboa Moreno, E., et al., *A Pilot Study to Assess the Feasibility of the Spanish Diabetes Self-Management Program in the Basque Country.* Journal of diabetes research, 2016. **2016**: p. 9145673-9145673.
45. Goode, A.D., M.M. Reeves, and E.G. Eakin, *Telephone-delivered interventions for physical activity and dietary behavior change: an updated systematic review.* Am J Prev Med, 2012. **42**(1): p. 81-88.
46. Grant, R.W., et al., *Exercise as a vital sign: A quasi-experimental analysis of a health system intervention to collect patient-reported exercise levels.* Journal of General Internal Medicine, 2014. **29**(2): p. 341-348.
47. Healey, E.L., et al., *The feasibility and acceptability of a physical activity intervention for older people with chronic musculoskeletal pain: The iPOPP pilot trial protocol.* Musculoskeletal Care, 2018. **16**(1): p. 118-132.
48. Hinrichs, T., et al., *General practitioner advice on physical activity: analyses in a cohort of older primary health care patients (getABI).* BMC family practice, 2011. **12**(1): p. 26.
49. Hinrichs, T., et al., *Effects of an exercise programme for chronically ill and mobility-restricted elderly with structured support by the general practitioner's practice (HOMEfit) - study protocol of a randomised controlled trial.* Trials, 2011. **12**.
50. Hinrichs, T., et al., *Home-Based Exercise Supported by General Practitioner Practices: Ineffective in a Sample of Chronically Ill, Mobility-Limited Older Adults (the HOMEfit Randomized Controlled Trial).* Journal of the American Geriatrics Society, 2016. **64**(11): p. 2270-2279.
51. Holden, M.A., et al., *Role of exercise for knee pain: What do older adults in the community think?* Arthritis Care and Research, 2012. **64**(10): p. 1554-1564.
52. Hurley, M., et al., *Exercise interventions and patient beliefs for people with hip, knee or hip and knee osteoarthritis: A mixed methods review*, 2018, John Wiley and Sons Ltd.
53. Husk, K., et al., *What approaches to social prescribing work, for whom, and in what circumstances? A realist review.* Health & social care in the community., 2019.
54. James, E.L., et al., *Referral for Expert Physical Activity Counseling: A Pragmatic RCT.* American Journal of Preventive Medicine 2017. **53**(4): p. 490–499.
55. Jansink, R., et al., *Primary care nurses struggle with lifestyle counseling in diabetes care: a qualitative analysis.* BMC family practice, 2010. **11**(1): p. 41.
56. Jones, G.R., et al., *Development of a physical literacy model for older adults–a consensus process by the collaborative working group on physical literacy for older Canadians.* 2018.
57. Jorgensen, T.K., M. Nordentoft, and J. Krogh, *How do general practitioners in Denmark promote physical activity?* Scandinavian Journal of Primary Health Care, 2012. **30**(3): p. 141-146.
58. Kosteli, M.C., et al., *Barriers and enablers of physical activity engagement for patients with COPD in primary care.* International Journal of COPD, 2017. **12**: p. 1019-1031.
59. Lamming, L., et al., *What do we know about brief interventions for physical activity that could be delivered in primary care consultations? A systematic review of reviews.* Prev Med, 2017. **99**: p. 152-163.

For peer review only

60. Leemrijse, C.J., et al., *Collaboration of general practitioners and exercise providers in promotion of physical activity a written survey among general practitioners.* BMC Family Practice, 2015. **16**(1): p. 1-9.
61. Leenaars, K.E., et al., *The role of the care sport connector in the Netherlands.* Health Promotion International, 2016.
62. Leijon, M.E., et al., *Physical activity referrals in Swedish primary health care - Prescriber and patient characteristics, reasons for prescriptions, and prescribed activities.* BMC Health Services Research, 2008. **8**.
63. Lindeman, C., et al., *The extent to which family physicians record their patients' exercise in medical records: a scoping review.* BMJ open, 2020. **10**(2).
64. Lion, A., et al., *Physical activity promotion in primary care: a Utopian quest?* Health promotion international, 2019. **34**(4): p. 877-886.
65. Lobelo, F., J. Duperly, and E. Frank, *Physical activity habits of doctors and medical students influence their counselling practices.* Br J Sports Med online, 2009. **43**(2): p. 89-92.
66. Lohmann, H., V. Siersma, and N.F. Olivarius, *Fitness consultations in routine care of patients with type 2 diabetes in general practice: An 18-month non-randomised intervention study.* BMC Family Practice, 2010. **11**.
67. Loughren, E.A., C. Baker, and D. Crone, *'Let's Get Moving'Physical Activity Care Pathway (Gloucestershire) Post-Programme Evaluation Report.*, 2014: http://eprints.glos.ac.uk/2378/.
68. Martín-Borràs, C., et al., *A new model of exercise referral scheme in primary care: is the effect on adherence to physical activity sustainable in the long term? A 15-month randomised controlled trial.* BMJ Open, 2018. **8**(3): p. e017211-e017211.
69. McDonough, S.M., et al., *Pedometer-driven walking for chronic low back pain: A feasibility randomized controlled trial.* Clinical Journal of Pain, 2013. **29**(11): p. 972-981.
70. McKay, H.G., et al., *The Diabetes Network Internet-Based Physical Activity Intervention A randomized pilot study*, 2001.
71. Melillo, K.D., et al., *Perceptions of nurse practitioners regarding their role in physical activity and exercise prescription for older adults.* Clinical excellence for nurse practitioners : the international journal of NPACE, 2000. **4**(2): p. 108-116.
72. Moore, G.F., et al., *Mixed-method process evaluation of the welsh national exercise referral scheme.* Health Education 2013.
73. Morgan, G.S., et al., *Physical ACtivity facilitation for Elders (PACE): Study protocol for a randomised controlled trial.* Trials, 2015. **16**(1).
74. Morishita, Y., et al., *Primary care physicians' own exercise habits influence exercise counseling for patients with chronic kidney disease: A cross-sectional study.* BMC Nephrology, 2014. **15**(1).
75. Faculty of Sport and Exercise Medicine. *Moving Medicine*. 2018.
76. Muellmann, S., et al., *Effectiveness of eHealth interventions for the promotion of physical activity in older adults: A systematic review.* Prev Med, 2018. **108**: p. 93-110.
77. Murphy, S., et al., *An exploratory cluster randomised trial of a university halls of residence based social norms intervention in Wales, UK.* BMC Public Health, 2012. **12**(186): p. 1471-2458.
78. NHS Leading Change Adding Value Team. *Introducing group consultations for adults with Type 2 diabetes*. 2019 2020].
79. National Institute for Clinical Excellence, *Dementia, disability and frailty in later life – mid-life approaches to delay or prevent onset*, 2015.
80. National Institute for Clinical Excellence, *Physical activity: brief advice for adults in primary care.* London: Nice public health guidance 2013: p. 44.
81. National Institute for Clinical Excellence, *Behaviour change: individual approaches*, 2014.
82. National Institute for Clinical Excellence, *Physical activity: exercise referral schemes*, 2014.

For peer review only

83. National Institute for Clinical Excellence. *Making Every Contact Count: How NICE resources can support local priorities*. [cited 2019; Available from: https://stpsupport.nice.org.uk/mecc/index.html.
84. Omura, J.D., et al., *Primary care providers' physical activity counseling and referral practices and barriers for cardiovascular disease prevention.* Preventive Medicine, 2018. **108**: p. 115-122.
85. Rena Parish, T. and B.B.S. Tracie Rena Parish, *Examination of the constructs of the Transtheoretical model in patients with heart failure: a focus on physical activity readiness*, 2006.
86. RCGP and Parkrun. *parkrun UK teams up with RCGP to 'prescribe' active lifestyles to patients and practice staff* 21st June 2018 [cited 2019.
87. Persson, G., et al., *Physical activity on prescription (PAP) from the general practitioner's perspective–a qualitative study.* BMC family practice, 2013. **14**(1): p. 128.
88. Pescheny, J.V., Y. Pappas, and G. Randhawa, *Facilitators and barriers of implementing and delivering social prescribing services: a systematic review.* BMC health services research, 2018. **18**(1): p. 86.
89. Prochaska, J.J., et al., *PACE Interactive Communication Technology for Behavior Change in Clinical Settings*, 2000. p. 127-131.
90. Department of Health. *Resources for commissioning Let's Get Moving interventions* 2012 05/04/2020].
91. Quirk, H. and S. Haake, *How can we get more people with long-term health conditions involved in parkrun? A qualitative study evaluating parkrun's PROVE project.* BMC Sports Sci Med Rehabil, 2019. **11**(1): p. 22.
92. Rhodes, R.E., et al., *Increasing physical activity by four legs rather than two: systematic review of dog-facilitated physical activity interventions.* British Journal of Sports Medicine, 2020.
93. Royal College of General Practitioners. *RCGP Active Practice Charter*. 2019 [cited 2019; Available from: https://r1.dotdigital-pages.com/p/49LX-5IR/active-practice-charter.
94. Rushforth, B., et al., *Barriers to effective management of type 2 diabetes in primary care: qualitative systematic review.* Br J Gen Pract, 2016. **66**(643): p. e114-e127.
95. Savill, B., A. Murray, and R. Weiler, *Is general practice engaged with physical activity promotion?* Br J Gen Pract, 2015. **65**(638): p. 484-485.
96. Schofield, G., K. Croteau, and G. McLean, *Trust levels of physical activity information sources: a population study.* Health Promotion Journal of Australia, 2005. **16**(3): p. 221-224.
97. Shaw, R., et al., *Pre-exercise screening and health coaching in CHD secondary prevention: A qualitative study of the patient experience.* Health Education Research, 2012. **27**(3): p. 424-436.
98. Short, C.E., et al., *Physical activity recommendations from general practitioners in Australia. Results from a national survey.* Australian and New Zealand Journal of Public Health, 2016. **40**(1): p. 83-90.
99. Smith, S.M., et al., *Interventions for improving outcomes in patients with multimorbidity in primary care and community settings.* Cochrane Database of Systematic Reviews, 2016(3).
100. Smith, T.O., et al., *Social prescribing programmes to prevent or delay frailty in community-dwelling older adults.* Geriatrics, 2019. **4**(4): p. 65.
101. Stone, R.C. and J. Baker, *Painful choices: a qualitative exploration of facilitators and barriers to active lifestyles among adults with osteoarthritis.* Journal of Applied Gerontology, 2015. **36**(9): p. 1091-1116.
102. Sturgiss, E., et al., *Increasing general practitioners' confidence and self-efficacy in managing obesity: a mixed methods study* BMJ open 2017. **7**(1): p. e014314.
103. Department of Health, P.A., Health Improvement and Protection, *Start Active, Stay Active: A report on physical activity from the four home countries' Chief Medical Officers (Now*

For peer review only

updated), 2011: https://assets.publishing.service.gov.uk/government/uploads/system/uploads/attachment_data/file/830943/withdrawn_dh_128210.pdf.

104. UK CMOs, *Physical activity guidelines: UK chief medical officers' report*, 2019: https://www.gov.uk/government/publications/physical-activity-guidelines-uk-chief-medical-officers-report.

105. US Department of Health and Human Services, *Physical Activity Guidelines for Americans, 2nd edition*, 2018: Washington, DC.

106. Van der Wulp, I., et al., *Effectiveness of peer-led self-management coaching for patients recently diagnosed with Type 2 diabetes mellitus in primary care: A randomized controlled trial.* Diabetic Medicine, 2012. **29**(10).

107. van Sluijs, E.M.F., et al., *Effect of a tailored physical activity intervention delivered in general practice settings: results of a randomized controlled trial.* American Journal of Public Health, 2005. **95**(10): p. 1825-1831.

108. Van Sluijs, E.M.F., et al., *The positive effect on determinants of physical activity of a tailored, general practice-based physical activity intervention.* Health education research, 2005. **20**(3): p. 345-356.

109. Vanroy, J., et al., *Short- and long-term effects of a need-supportive physical activity intervention among patients with type 2 diabetes mellitus: A randomized controlled pilot trial.* PLoS ONE, 2017. **12**(4).

110. Verwey, R., et al., *A pilot study of a tool to stimulate physical activity in patients with COPD or type 2 diabetes in primary care.* Journal of Telemedicine and Telecare, 2014. **20**(1): p. 29-34.

111. Verwey, R., et al., *A monitoring and feedback tool embedded in a counselling protocol to increase physical activity of patients with COPD or type 2 diabetes in primary care: Study protocol of a three-arm cluster randomised controlled trial.* BMC Family Practice, 2014. **15**(1).

112. Verwey, R.D.S.e., et al. *Upgrading physical activity counselling in primary care in the Netherlands*. Oxford University Press.

113. Verwey, R., et al., *Process evaluation of physical activity counselling with and without the use of mobile technology: A mixed methods study.* International Journal of Nursing Studies, 2016. **53**: p. 3-16.

114. Walsh, J.M.E., et al., *Exercise Counseling by Primary Care Physicians in the Era of Managed Care*, 1999.

115. Ward, M., *A Survey of Physical Activity in MedicalCurricula: A report of the HEPA in Health Care Settings*, 2015: HEPA Europe Working Group.

116. Weiler, R., et al., *Physical activity education in the undergraduate curricula of all UK medical schools. Are tomorrow's doctors equipped to follow clinical guidelines?* British journal of sports medicine, 2012. **46**(14): p. 1024-1026.

117. Weinstock, R.S., et al., *Lessened decline in physical activity and impairment of older adults with diabetes with telemedicine and pedometer use: results from the IDEATel study.* Age and Ageing, 2011. **40**(1): p. 98-105.

118. Wheeler, P.C., et al., *Primary care knowledge and beliefs about physical activity and health: a survey of primary healthcare team members.* BJGP open, 2017. **1**(2).

119. Wilcox, S., et al., *Adoption and Implementation of Physical Activity and Dietary Counseling by Community Health Center Providers and Nurses*, 2004.

120. Williams, S.L., et al., *Translating a walking intervention for health professional delivery within primary care: A mixed-methods treatment fidelity assessment.* Br J Health Psychol, 2019. **25**(1): p. 17-38.

121. Wormald, H. and L. Ingle, *GP exercise referral schemes: Improving the patient's experience.* Education Journal,, 2004. **63**(4): p. 362-373

For peer review only

For peer review only

Supplementary table 5: Taxonomy of primary care physical activity interventions for people with long-term conditions

Type of intervention	Description	Example papers
Brief interventions	Brief verbal advice, discussion, negotiation or encouragement, with or without written or other support or follow-up. It can vary from basic advice to a more extended, individually focused discussion	**Lamming (2017) – systematic review of reviews [1]**<br>Brief Interventions can increase self-reported physical activity in the short term, but there is insufficient evidence about their long-term impact, their impact on objectively measured physical activity, and about the factors that influence their effectiveness, feasibility and acceptability.
Telephone interventions	Physical activity interventions delivered predominantly over the telephone	**Goode (2012) – systematic review [2]**<br>Findings provide continuing strong evidence supporting the efficacy of telephone-delivered interventions to promote physical activity.
Online/'eHealth' interventions	The use of information and communication technologies for health"(WHO, 2015).	**Muellmann (2018) – systematic review [3]**<br>'eHealth' interventions can effectively promote PA in older adults aged 55 years and above in the short-term, while evidence regarding long-term effects and the added benefit of eHealth compared to non-eHealth intervention components is still lacking.
Exercise referral schemes	The practice of referring a person from primary care to a qualified exercise professional who uses relevant medical information about the person to develop a tailored programme of physical activity usually lasting from 10 to 12 weeks.	**Campbell (2015) – systematic review [4]**<br>Compared with usual care, exercise referral schemes had a small effect in increasing the proportion of individuals achieving 90–150 min of at least moderate intensity activity per week.
Community 'navigators'	Appropriately trained individuals who can guide patients towards local physical activity opportunities.	**Leenaars 2017 – Care Sport Connectors (CSCs)[5]**<br>Introduced in 2012 in the Netherlands and seem to hold the promise of improving collaboration between the primary care and the physical activity sector, especially because the roles that CSCs perceive themselves as having seem to be directed at eliminating barriers in this connection.
Referral to exercise specialists (e.g. exercise physiologists)	Referral to a qualified exercise physiologist from primary care (implemented in Australia)	**Ewald 2018 – Randomised controlled trial to establish cost-effectiveness [6]**<br>Referral to an exercise physiologist increased physical activity, which persisted at 9 month follow-up. Coaching achieved a modest increase in activity equivalent to 10 minutes walking per day, at a cost of AUD$245

		(approx. £150) per person. Face-to-face and telephone counselling were both effective. Note: a change in quality of life not observed so could not estimate utility.
Intervention delivery by existing primary care staff	When primary care professionals (e.g. practice nurses, healthcare assistants) deliver physical activity interventions	**Williams et al (2019) – A mixed-methods treatment fidelity assessment [7]**<br>Two practice nurses and six health care assistants delivered a theory-based walking intervention to 63 patients in their own practices. High levels of fidelity of delivery were demonstrated. However, patient-, provider-, and component-level factors impacted on treatment delivery and receipt.
Physical activity 'pathways'	'Let's Get Moving'; a UK-based attempt at embedding physical activity promotion into routine primary care, recommended by Public Health England for commissioning at a local level by Primary Care Trusts within NHS England	**Bull 2008, 2010 – Feasibility pilot study and process evaluation [8, 9]**<br>• Recruitment low, particularly in practices recruiting opportunistically vs. disease register<br>• Multiple promotion strategies needed<br>• Active patients did not move through 'exit' pathway as intended<br>• Professionals modified intervention to meet time available<br>• Needs better integration with existing referral pathways (e.g. ERS)<br>• Inventory of local opportunities would be helpful<br>**Boehler 2011 – 'the costs of changing physical activity behaviour' [10]**<br>Disease register screening is more costly than opportunistic patient recruitment (£53 vs £191). However, additional costs come with a higher completion rate and better outcomes in terms of behavioural change in patients completing the care pathway.<br>**Department of Health commissioning guidance (2012) –** includes resources and protocols for implementation [11]<br>**Loughren, 2014 – 'Let's Get Moving' Physical Activity Care Pathway (Gloucestershire) [12]**<br>Factors determining implementation success:<br>• Time required to deliver lifestyle counselling<br>• Integration/competition with existing ERS<br>• On-going support for deliverers in coordination<br>**Barrett 2017 – Feasibility in primary care physiotherapy [13]**<br>Concluded to be a clinically feasible resource to primary care physiotherapists with some

For peer review only

		modifications and with the support of additional resources (i.e. professional training and integration with existing schemes)
Practice-wide initiatives	Active Practice Charter promoted by the Royal College of General Practitioners	**Active Practice Charter (2019)** [14]<br>The Royal College of GPs and Sport England have launched the Active Practice Charter to inspire and celebrate GP practices that are taking steps to increase activity and reduce sedentary behaviour in their patients and staff.
Community initiatives adopted by primary care	*Parkrun* practice	**Quirk and Haarke (2019) [15]** - *Parkrun* launched a project called PROVE in 2016 to engage people living with long-term health conditions in England. Over the 3 year project, *Parkrun* appointed volunteer outreach ambassadors with a specialist interest in the health condition they represented whose role was to ensure *parkrun* was welcoming, supportive and inclusive. A qualitative evaluation showed that PROVE was regarded by the ambassadors (patients, carers, professionals) as important for ensuring that people with long-term health conditions can engage in physical activity and volunteering in a safe and supportive environment.
A whole system approach to embed physical activity in clinical practice	Moving healthcare professionals programme (MHPP)	**Brannan et al (2019) [16] –** The MHPP model is a partnership between Public Health England and Sport England. It has delivered face-to-face training to 17,105 healthcare professionals, embedded materials in almost three quarters of medical schools in England and overseen > 95,000 e-learning modules over two and half years. The programme aims to bring about improvements in knowledge, skills and practice. Individual elements of the model are being evaluated and further evaluation is planned to assess patient impact.
	Moving Medicine	**'Moving Medicine' [17]** was created to aid healthcare workers in integrating conversations about physical activity during routine clinical care. Moving medicine offers this online support for all patients of all ages and conditions. It also offers advice depending on the amount of time available with a patient and adjusts the conversations to these needs. Moving Medicine has the option of completing an 'online physical activity training course, which is accredited with 16 CPD points from the Faculty of Sport and Exercise Medicine (UK).

For peer review only

Multi-faceted interventions	Interventions involving a combination of approaches	**Avery et al (2016) [18] –** 'Movement as Medicine for Type 2 diabetes' is a multifaceted intervention informed by the theory of planned behaviour and social cognitive theory and consisted of 15 behaviour change techniques. It includes an accredited online training programme for healthcare professionals who deliver a behavioural intervention for adults with type 2 diabetes. This. Intervention intensity and duration were informed by a systematic review and stakeholder work. Usability testing resolved technical problems with using the online training intervention on practice IT systems. An open pilot study of the intervention identified mechanisms to enhance intervention implementation during routine diabetes consultations.
**Campaigns**	Age UK Resources – 'We Are Undefeatable'	The '**We Are Undefeatable' [19]** campaign was developed by 15 leading health and social care charities. Its main aim is to support and encourage ways for individuals with a wide range of health conditions, to remain active even during times where this can be challenging.

Reference list

1. Lamming, L., et al., *What do we know about brief interventions for physical activity that could be delivered in primary care consultations? A systematic review of reviews.* Prev Med, 2017. **99**: p. 152-163.
2. Goode, A.D., M.M. Reeves, and E.G. Eakin, *Telephone-delivered interventions for physical activity and dietary behavior change: an updated systematic review.* Am J Prev Med, 2012. **42**(1): p. 81-88.
3. Muellmann, S., et al., *Effectiveness of eHealth interventions for the promotion of physical activity in older adults: A systematic review.* Prev Med, 2018. **108**: p. 93-110.
4. Campbell, F., et al., *A systematic review and economic evaluation of exercise referral schemes in primary care: a short report.* Health Technol Assess, 2015. **19**(60).
5. Leenaars, K.E., et al., *Exploring the impact of the care sport connector in the Netherlands.* BMC public health, 2017. **17**(1): p. 813.
6. Ewald, B., et al., *Physical activity coaching by Australian Exercise Physiologists is cost effective for patients referred from general practice.* Aust N Z J Public Health, 2018. **42**(1): p. 12-15.
7. Williams, S.L., et al., *Translating a walking intervention for health professional delivery within primary care: A mixed-methods treatment fidelity assessment.* Br J Health Psychol, 2019. **25**(1): p. 17-38.
8. Bull, F., K. Milton, and C. Boehler, *Evaluation of the Physical Activity Care Pathway London Feasibility Pilot–Final Technical Report*, 2008: https://assets.publishing.service.gov.uk/government/uploads/system/uploads/attachment_

data/file/192041/Evaluation_of_the_Physical_Activity_Care_Pathway_London_Feasibility_Pilot_-_Report.pdf.
9. Bull, F.C. and K.E. Milton, *A process evaluation of a" physical activity pathway" in the primary care setting.* BMC Public Health, 2010. **10**(1): p. 463.
10. Boehler, C., et al., *The cost of changing physical activity behaviour: evidence from a "physical activity pathway" in the primary care setting.* BMC Public Health, 2011. **11**(1): p. 370-370.
11. Department of Health. *Resources for commissioning Let's Get Moving interventions* 2012 05/04/2020].
12. Loughren, E.A., C. Baker, and D. Crone, *'Let's Get Moving'Physical Activity Care Pathway (Gloucestershire) Post-Programme Evaluation Report.*, 2014: http://eprints.glos.ac.uk/2378/.
13. Barrett, E.M., J. Hussey, and C.D. Darker, *Feasibility of a physical activity pathway for Irish primary care physiotherapy services.* Physiotherapy, 2017. **103**(1): p. 106-112.
14. Royal College of General Practitioners. *RCGP Active Practice Charter*. 2019 [cited 2019; Available from: https://r1.dotdigital-pages.com/p/49LX-5IR/active-practice-charter.
15. Quirk, H. and S. Haake, *How can we get more people with long-term health conditions involved in parkrun? A qualitative study evaluating parkrun's PROVE project.* BMC Sports Sci Med Rehabil, 2019. **11**(1): p. 22.
16. Brannan, M., et al., *Moving healthcare professionals–a whole system approach to embed physical activity in clinical practice.* BMC Med Educ, 2019. **19**(1): p. 84.
17. Faculty of Sport and Exercise Medicine. *Moving Medicine*. 2018.
18. Avery, L., et al., *Systematic development of a theory-informed multifaceted behavioural intervention to increase physical activity of adults with type 2 diabetes in routine primary care: Movement as Medicine for Type 2 Diabetes.* Implement Sci, 2016. **11**(1).
19. Undefeatable, W.A. *We Are Undefeatable; About the campaign*. 2019 17 July 2020].

For peer review only

Realist And MEta-narrative Evidence Syntheses (RAMESES) Checklist

Section	Item	Page number
**Title**		
1.	In the title, identify the document as a realist synthesis or review	1
**Abstract**		
2.	While acknowledging publication requirements and house style, abstracts should ideally contain brief details of: the study's background, review question or objectives; search strategy; methods of selection, appraisal, analysis and synthesis of sources; main results; and implications for practice.	2
**Introduction**		
3. Rationale for review	Explain why the review is needed and what it is likely to contribute to existing understanding of the topic area.	4
4. Objectives and focus of review	State the objective(s) of the review and/or the review question(s). Define and provide a rationale for the focus of the review.	4-5
**Methods**		
5. Changes in the review process	Any changes made to the review process that was initially planned should be briefly described and justified.	13
6. Rationale for using realist synthesis	Explain why realist synthesis was considered the most appropriate method to use.	4
7. Scoping the literature	Describe and justify the initial process of exploratory scoping of the literature.	6
8. Searching processes	While considering specific requirements of the journal or other publication outlet, state and provide a rationale for	6

For peer review only

	how the iterative searching was done. Provide details on all the sources accessed for information in the review. Where searching in electronic databases has taken place, the details should include, for example, name of database, search terms, dates of coverage and date last searched. If individuals familiar with the	
9. Selection and appraisal of documents	Explain how judgements were made about including and excluding data from documents, and justify these.	6
10. Data extraction	Describe and explain which data or information were extracted from the included documents and justify this selection.	6
11. Analysis and synthesis processes	Describe the analysis and synthesis processes in detail. This section should include information on the constructs analysed and describe the analytic process.	6
**Results**		
12. Document flow diagram	Provide details on the number of documents assessed for eligibility and included in the review with reasons for exclusion at each stage as well as an indication of their source of origin (for example, from searching databases, reference lists and so on). You may consider using the example templates (which are likely to need modification to suit the data) that are provided.	6, Figure 2
13. Document characteristics	Provide information on the characteristics of the documents included in the review.	6, Figure 2

For peer review only

14. Main findings	Present the key findings with a specific focus on theory building and testing.	7-11
**Discussion**		
15. Summary of findings	Summarize the main findings, taking into account the review's objective(s), research question(s), focus and intended audience(s).	12
16. Strengths, limitations and future research directions	Discuss both the strengths of the review and its limitations. These should include (but need not be restricted to) (a) consideration of all the steps in the review process and (b) comment on the overall strength of evidence supporting the explanatory insights which emerged. The limitations identified may point to areas where further work is needed.	13-15
17. Comparison with existing literature	Where applicable, compare and contrast the review's findings with the existing literature (for example, other reviews) on the same topic.	14
18. Conclusion and recommendations	List the main implications of the findings and place these in the context of other relevant literature. If appropriate, offer recommendations for policy and practice.	14-15
19. Funding	Provide details of funding source (if any) for the review, the role played by the funder (if any) and any conflicts of interests of the reviewers.	15

For peer review only
